# Molybdenum Alkylidyne Complexes with Tripodal Silanolate Ligands: The Next Generation of Alkyne Metathesis Catalysts

**DOI:** 10.1002/anie.201908571

**Published:** 2019-09-17

**Authors:** Julius Hillenbrand, Markus Leutzsch, Alois Fürstner

**Affiliations:** ^1^ Max-Planck-Institut für Kohlenforschung 45470 Mülheim/Ruhr Germany

**Keywords:** alkylidyne complexes, alkyne metathesis, molybdenum, podands, silanolate ligands

## Abstract

A new type of molybdenum alkylidyne catalysts for alkyne metathesis is described, which is distinguished by an unconventional podand topology. These structurally well‐defined complexes are easy to make on scale and proved to be tolerant toward numerous functional groups; even certain protic substituents were found to be compatible. The new catalysts were characterized by X‐ray crystallography and by spectroscopic means, including ^95^Mo NMR.

## Graphical abstract

**The canopy**: Molybdenum alkylidyne complexes with triarylsilanolate ligands are privileged catalysts for alkyne metathesis, but tethering of the silyl groups seems to make things even better. Crystallographic data and a rare case study based on ^95^Mo NMR spectroscopy provide insights into why that is so.

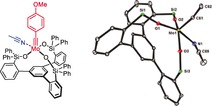

The discovery that triarylsilanolate ligands synergize exceedingly well with molybdenum alkylidynes marks a milestone in the development of alkyne metathesis.^1^, ^2^, ^3^ Catalysts such as **1** or the derived bench‐stable adduct **2** combine excellent activity with user‐friendliness; most importantly, they are distinguished by a remarkable and so far unrivaled functional group tolerance.^4^, ^5^, ^6^ This auspicious profile is manifest, inter alia, in the high‐yielding formation of numerous poly‐functionalized targets; the examples shown in Figure 1 are representative.^7^, ^8^, ^9^, ^10^, ^11^, ^12^, ^13^, ^14^ Even protic groups were found to be compatible in favorable cases, although important limitations still remain:^15^ the polarization of the operative [Mo≡CR] unit, which is inherently nucleophilic and basic at carbon, constitutes a potential Achilles heel of any Schrock alkylidyne in a protic environment.^16^, ^17^ Moreover, substrates able to entail ligand exchange will eventually bring productive alkyne metathesis to a hold since catalyst **1** and congeners draw their excellent performance from a (largely) intact siloxide ligand sphere. In consideration thereof, it may not come as a surprise that the attempted metathesis reaction of a substrate as simple as **8** containing an unhindered primary alcohol basically met with failure (Scheme 1).

**Figure 1:**
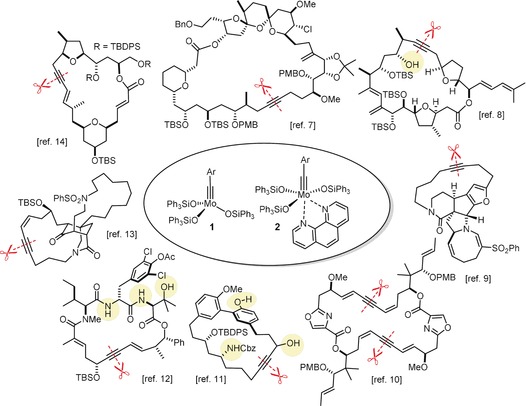
Selected examples from the cornucopia of products formed by alkyne metathesis at the indicated site (scissors) with the aid of molybdenum alkylidynes such as **1** or the derived adduct **2**; tolerated protic groups are marked in yellow.

**Scheme 1:**
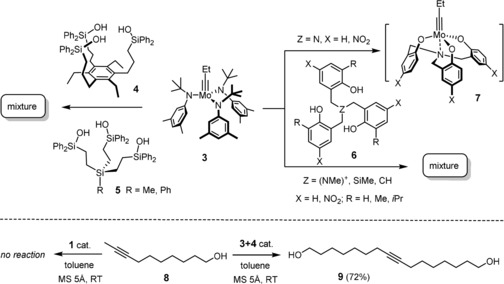
Previous experimentation with potential podand ligands.

We conceived that the use of chelating ligands might allow this issue to be addressed, at least in part, since the concomitant cleavage of up to three Mo−O−Si bonds is statistically less likely. In a first foray, the tripodal ligand scaffolds **4** and **5** were prepared (Scheme 1).^18^ Upon mixing with precatalyst **3**, however, they fail to afford well‐defined alkylidyne complexes; rather, the available data suggest that a mixture is generated as a result of partial cross linking. This aspect notwithstanding, these two‐component catalysts show high activity and, indeed, a gradually improved tolerance toward functional groups which the parent complex **1** does not allow to handle; the structurally simple but chemically challenging primary alcohol **8** referred to above is one of them, which afforded the corresponding metathesis product **9** in up to 72 % yield.^18^, ^19^

Other potentially tripodal ligands were explored by Zhang and co‐workers: originally, their design relied on the use of amine‐tethered (nitro)phenol groups **6** (Z=N; X=H, NO_2_). Although derived podand complexes were isolated and characterized, only the in situ generated mixture showed appreciable catalytic activity.^20^, ^21^ This system suffers from the internal coordination of the basic N‐atom onto the molybdenum center, which compromises the Lewis acidity of the alkylidyne unit.^20^, ^22^ To remedy the issue, the ammonium‐, silicon‐ and carbon‐tethered variants of **6** (Z=(NMe)^+^, SiMe, CH) were prepared, which do indeed entail higher activities.^23^ For the lack of the anchoring *N*‐atom, however, more than one active species is generated on reaction with **3**; rigorous proof of formation of well‐defined catalysts of podand topology is missing for any of these systems.^24^, ^25^, ^26^

It is against this backdrop that the current study must be seen. The next‐generation catalyst design should allow the following aspects to be addressed: (i) a tridentate arylsilanolate ligand scaffold continues to be most desirable given the well‐proven synergy with the catalytically active [Mo≡CR] unit; (ii) in order to reach a well‐defined single‐component catalyst, the ligand has to be more preorganized than **4** or **5** to minimize the entropic penalty upon formation of the targeted podand topology and hence avoid competing oligomerization from occurring; (iii) importantly, the necessary level of preorganization must not entail an overly rigidified backbone, because any active catalyst has to support different geometries while passing through the catalytic cycle;^1^, ^2^, ^3^ specifically, the ligand needs to accommodate the tetrahedral geometry of the resting state as well as the trigonal bipyramidal extreme to be adopted during substrate binding and metallacycle formation/processing; (iv) this essential compromise between preorganization and flexibility has to be reached without recourse to a Lewis‐basic heteroelement in the tether; (v) ideally, the ligand synthesis is straightforward, scalable and modular, as deemed necessary for future fine‐tuning of the ligand properties in steric and/or electronic terms; (vi) an additional desideratum is the use of a molybdenum precatalyst other than **3**: although this complex can be prepared on scale, its synthesis mandates particularly rigorous organometallic techniques and access to an argon (not nitrogen!) Schlenk‐line/glove‐box.^27^

We thought to meet these stringent criteria by the design shown in Scheme 2. A literature‐known cyclocondensation reaction converts 2‐bromoacetophenone (**10**) into **11** on scale.^28^, ^29^ Exhaustive metal/halogen exchange followed by quenching of the lithiated intermediate with Ph_2_Si(OMe)_2_ furnishes **12 a**,^30^ from which the desired trisilanol **13 a** is released on treatment with aqueous HCl. This simple three‐step sequence gave >2 g of **13 a** (single largest batch); we have no reason to believe that it would not work similarly well on even larger scale. Moreover, the preparation of the analogous silanol **13 b** bearing lateral MeO‐ groups suggests that the route will qualify for more systematic ligand variation once the project enters the next stage of development.

**Scheme 2:**
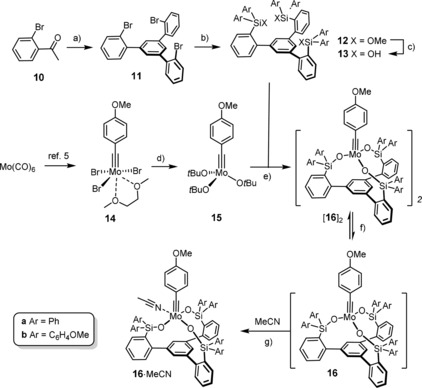
a) TfOH (neat), 130 °C, 59 %; b) *t*BuLi, Et_2_O, −125 °C→RT, then Ar_2_Si(OMe)_2_, 74 % (**12 a**), 12 % (**12 b**, unoptimized); c) aq. HCl (12 m), THF, quant. (**13 a**), quant. (**13 b**); d) NaO*t*Bu, THF, 83 %; e) **13**, toluene, 95 % ([**16 a**]_2_), 76 % ([**16 b**]_2_); f) toluene, 60 °C, see text; g) MeCN, see text.

The structure of **13 a** in the solid state (Figure 2) shows that all three Si−OH groups are “upward/inward”‐oriented; NMR data prove that the approximate *C*_3_ symmetry is maintained in solution at ambient temperature. Although this conformation is arguably the consequence of a hydrogen‐bonding array with a co‐crystallized water molecule, it indicates that the *C*_3_‐symmetric orientation, as necessary for the formation of a podand complex, is geometrically and entropically viable.

**Figure 2:**
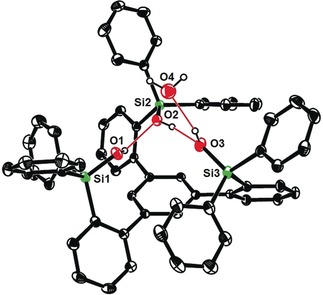
Structure of **13 a**⋅H_2_O in the solid state; only the H‐atoms of the Si‐OH groups involved in hydrogen bonding with the co‐crystallized water molecule (disordered over two positions) are shown for clarity.

Next, we addressed the problem of rendering the actual complex formation more practical by avoiding the use of **3** as the molybdenum source. The readily accessible tribromide [Br_3_Mo≡CAr(dme)] (**14**) might be conceived as the starting point of choice, because its treatment with Ph_3_SiOM (M=Na, K) is known to afford **1** as the parent catalyst of this family in high yield.^4^, ^5^ Adaptation of this procedure to the formation of the targeted podand complexes, however, is arguably unrewarding: (i) dipole repulsion in the triple sodium (lithium, potassium) salts derived from **13** almost certainly enforces a conformational change away from the “all‐inward” orientation of the Si‐O vectors;^30^ (ii) such triple salts are poorly soluble in toluene and even THF; (iii) salt metathesis with the Mo−Br of **14** bonds is irreversible; successful formation of a podand structure, however, seems more likely if the reaction has the chance to correct eventual “mistakes” such as undesirable cross‐linking.

Gratifyingly, interposition of a ligand exchange step with *t*BuONa provides a very convenient solution (Scheme 2). Complex **15** thus formed is catalytically inactive^31^ but the *tert*‐butoxy groups are more basic than a triarylsilanol; therefore, addition of **13** to a solution of **15** in toluene at ambient temperature results in quantitative ligand exchange. As an additional advantage we note that the released *t*BuOH can be conveniently removed in vacuo to give a new complex in the form of a bright yellow, gently air‐sensitive powder.^32^ This material is only sparingly soluble in [D_8_]toluene: although the recorded spectra speak for the presence of a well‐defined entity rather than an oligomer, the signal pattern is too complicated for an approximately *C*_3_‐symmetric target complex of type **16**. Importantly, however, significant spectral changes are observed upon stirring of the mixture at elevated temperatures: a new species is quantitatively formed after 60 min at 60 °C, which seems to correspond to the expected complex. In consideration of the fact that even the parent catalyst **1** is a non‐covalent dimer (at least) in the solid state held together by multiple π/π‐interactions between the lateral phenyl rings,^5^ the observed temperature dependence suggested that the yellow crude product might also be a self‐assembled dimeric or oligomeric entity that converts into the monomeric complex upon gentle heating. Once formed, the monomer is stable in solution for days; however, it is the aggregate which precipitates upon increasing the concentration and/or cooling of the mixture.

Although an X‐ray structure of this yellow compound has not yet been obtained, the notion of a pre‐equilibrium is supported by DOSY spectra recorded at the point where a ≈1:1 mixture of two compounds was present in solution. The observed diffusion constants *D*=5.56×10^−10^ m^2^ s^−1^ and *D*=3.30×10^−10^ m^2^ s^−1^, respectively, suggest that the two species have considerably different molecular weights/sizes; actually, the predicted *D*‐values for the monomer **16 a** and a likely dimeric (or potentially even trimeric) species—tentatively assigned as [**16 a**]_2_—nicely match the recorded data.^32^ From a solution in benzene/MeCN we were able to isolate dark blue crystals of adduct **16 a**⋅MeCN suitable for X‐ray analysis. Figure 3 shows the expected monomeric podand‐like molybdenum alkylidyne complex bearing an “end‐on” bound MeCN ligand;^33^ with 163.2(2)°, however, the Mo1‐N1‐C69 angle notably deviates from linearity. Taking acetonitrile as a dummy for an incoming alkyne substrate, this structure allows the binding site of the catalyst to be assessed: though tripodal in nature, the ancillary ligand scaffold is obviously flexible enough to open a sizeable bay area that is likely able to accommodate even reasonably bulky compounds. The first coordination sphere about the metal center is approximately tetrahedral, reflecting the fact that the binding site *trans* to the alkylidyne is unoccupied yet efficiently blocked by the central phenyl ring forming the platform of the tripodal ligand scaffold. Although the Lewis‐acidic Mo^VI^ center will sense this π‐cloud in vicinity, the observed distances (3.5–4.0 Å) rule any direct bonding interactions out. With 1.742(2) Å, the length of the Mo≡C alkylidyne is in the normal range; likewise, the kink in the Mo≡C‐Ar axis (161.4(2)°) has correspondence in the structures of **1** and analogues.^4^, ^5^

**Figure 3:**
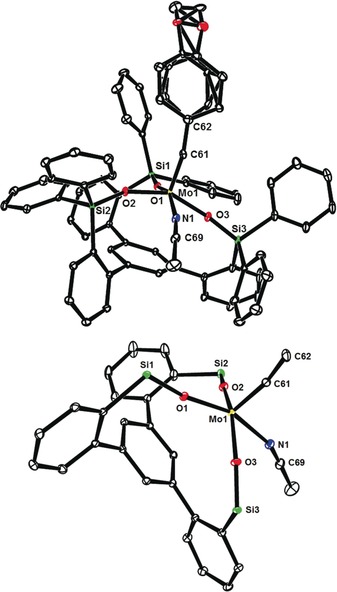
Top: structure of complex **16 a**⋅MeCN in the solid state with the alkylidyne unit disordered over two positions; bottom: truncated view of the podand core structure (all lateral aryl groups were removed; hydrogen atoms and one molecule of co‐crystallized but unbound MeCN are not shown for clarity).

Of particular interest are the remarkably obtuse Si‐O‐Mo bond angles (155.8(8), 163.2(2), 169.0(1)°), enforced by the ligand architecture; for comparison, we refer to the corresponding angles in **1** (141.3(1), 147.7(1), 159.5(1)°). The change in hybridization of a silanol O‐atom—on going from a tetrahedral (sp^3^) to a linear (sp) geometry—alters the degree of back‐donation of the lone pairs into the empty orbitals of the Mo atom and hence affects the Lewis acidity of the catalytically active alkylidyne unit.^5^ It has previously been pointed out that the eminent suppleness of an unrestrained Si‐O‐Mo linkage translates into an advantageous electronic adaptiveness: as a result, silanolate ligands bolster the different electronic optima of an alkyne metathesis catalyst when passing through the catalytic cycle.^5^ Under this premise, it is likely that the new podand complex **16** featuring uniformly stretched Mo‐O‐Si hinges is not just a tethered variant of the parent complex **1**; rather, its electronic character and, as a consequence thereof, the catalytic performance should differ from that of **1**.

^95^Mo NMR spectroscopy provides qualitative support for this notion. As a spin 5/2 nucleus with low natural abundance (≈15.9 %), low gyromagnetic ratio and a quadrupole moment, the ^95^Mo isotope is rarely used for analytical purposes in organometallic chemistry.^34^ Actually, the only previous investigation into molybdenum alkylidynes failed to detect the ^95^Mo NMR signal of [(*t*BuO)_3_Mo≡CPh].^35^ Gratifyingly though, we were able to record good spectra of complexes **1** and **16 a,b** in [D_8_]toluene at 60 °C (Figure 4). Although the largely missing precedent makes it currently impossible to interpret these data by comparison with known compounds, the distinct shift difference between **16 a** (*δ*=421 ppm) and **1** (*δ*=397 ppm) is taken as an indication for a subtle but noticeable difference in their electronic character.^36^ The fact that ‐OMe substituents on the peripheral phenyl rings of **16 b** cause a gentle up‐field shift (*δ*=414 ppm) speaks for a qualitative correlation between the chemical shift and the electron density at the Mo center: if this is the case, the podand complexes are slightly more Lewis acidic that the unrestrained parent complex **1**.^37^ Although this conclusion needs further scrutiny, we note that the alkylidyne C‐atoms ([D_8_]toluene) of monomeric **16 a** (*δ*_C_=310 ppm) and **16 b** (*δ*_C_=309 ppm) are also deshielded relative to that of **1** (*δ*_C_=301 ppm); the dimeric complex [**16 a**]_2_, in contrast, resonates at almost the same shift (*δ*_C_=298 ppm).

**Figure 4:**
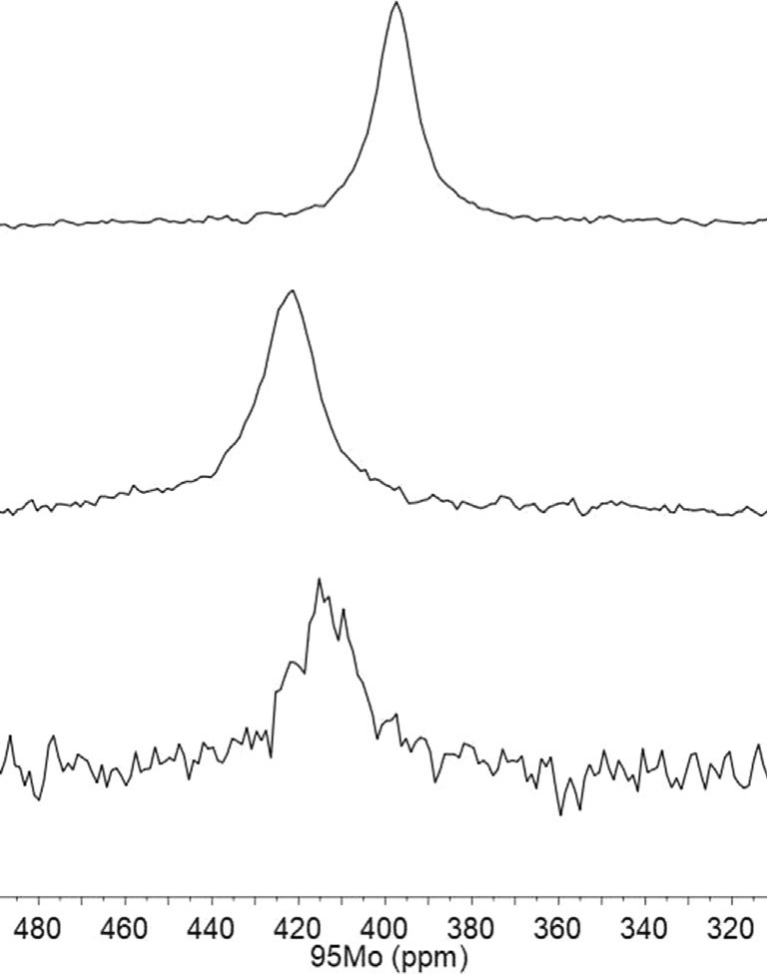
^95^Mo NMR spectra ([D_8_]toluene, 60 °C) of the parent complex **1** (top) and the podand complexes **16 a** (middle) and **16 b** (bottom).

The key measure for success, however, is the catalytic performance of the new podand complexes (Table 1). It is perhaps unsurprising that a rigidified backbone with reduced geometrical and electronic adaptability result in diminished reactivity: **16 a,b** (5 mol %) convert 1‐phenyl‐1‐propyne into tolane within ≤3 h at ambient temperature in the presence of molecular sieves (5 Å) as butyne‐sequestering agent,^4^ whereas complex **1** requires <10 min (for further direct comparison, see the SI or the footnotes to entries 1 and 10 in Table 1).^41^, ^42^ A normalized time scale analysis^43^ of different conversion/time diagrams suggests that the reaction is first order in catalyst **16 a**.^44^ From the application point of view it is gratifying that the dimer [**16 a**]_2_ and the monomeric podand **16 a** are equally effective.^32^ As expected, electron poor substrates are less reactive and mandate higher temperatures; the corresponding nitro derivative failed to react at 60 °C (entry 5).^15^, ^45^

**Table 1:** Alkyne metathesis reactions catalyzed by the alkylidyne complexes of podand topology.^[a]^

Entry	Catalyst	*T* (°C)	Product	Yield (%)^[b]^
1 2 3 4 5	**16 a** **16 a** **16 b** **16 b** **16 a**	RT 60 60 60 60	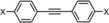	92 (X=OMe)^[c]^ 90 (X=CF_3_) 77 (X=CN)^[d]^ 93 (X=OH)^[e]^ NR (X=NO_2_)
				
6	**16 a**	RT	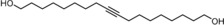	91
7	**16 a**	RT	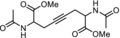	67
8	**16 a**	RT	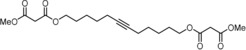	91
9	**16 a**	RT	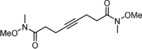	52
10	**16 a**	RT	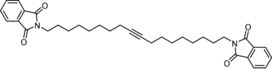	89^[f]^
11	**16 a**	60	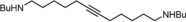	71
12	**16 a**	RT	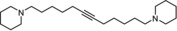	88
13	**16 a**	RT	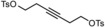	83
14 15	**16 a** **16 a**	60 RT	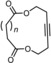	95 (*n*=1) 85 (*n*=3)
				
16	**16 a**	RT	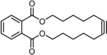	98
17	**16 a**	RT	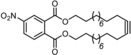	98
18	**16 a**	RT	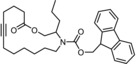	99^38^
19	**16 a**	RT	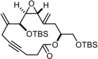	85^39^
20	**16 a**	60	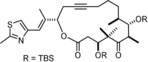	75^40^
21	**16 a**	110	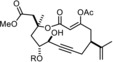	68^[g, 19]^

[a] Unless stated otherwise, all reactions were carried out using 5 mol % of catalyst in toluene at RT (ca. 23 °C) in the presence of powdered 5 Å MS; all substrates were internal alkynes carrying a methyl cap, which release 2‐butyne as by‐product of the metathesis reaction. [b] Isolated yield of analytically pure material. [c] The required reaction time was 9 h; for comparison, complex **1** (5 mol %) delivers the product in 82 % yield after 2 h. [d] **16 a** at 60 °C gave 78 % yield. [e] **16 a** at RT afforded only 48 % of the product together with recovered starting material. [f] The required reaction time was 6 h; for comparison, complex **1** (5 mol %) delivers the product in 73 % yield after 30 min. [g] Yield over two steps: in the actual metathesis product, *R*=2,2,6,6‐tetramethylpiperidin‐1‐yl; for the ease of isolation, the crude product was subjected to N−O bond cleavage with zinc dust in aq. THF/HOAc to give the diol product (R=H), see Ref. 19 and the SI; NR=no reaction.

Since a truly comprehensive study into functional group compatibility is beyond the scope of this initial foray, we focused on potentially problematic substituents. The high yielding homo‐metathesis of a primary alcohol is significant (entry 6); the obtained yield is actually higher than that of homologous **9** formed with the two‐component catalyst system [**3**+**4**] (Scheme 1).^18^ Additional substrates containing different protic sites (*sec*‐propargylic alcohol, phenol, amide, malonate, fluorenyl group) also gave appreciable results. Likewise, the compatibility with a secondary as well as tertiary amine (entries 11, 12) is a striking asset for a catalyst containing a high‐valent Mo‐center, as is the fact that a potentially chelating Weinreb amide group did not bring the reaction to a halt (entry 9); moreover, the stability of the elimination‐prone primary tosylate is noteworthy (entry 13). The different examples of ring closing alkyne metathesis reactions under high dilution conditions prove that the new catalysts qualify for this important transformation, even in challenging cases in which the products carry sensitive functionality, potential donor sites, or feature strained backbones (entries 14–21).

Overall, we conclude that an important step toward a new generation of structurally well‐defined alkyne metathesis catalysts has been made. The new design largely preserves the virtues of the parent tris(triarylsilanolate)molybdenum alkylidyne complex **1** but holds the promise of an even better functional group tolerance. Although it entails slower rates, we hope that full reactivity be restituted upon systematic variation of the ligand structure by harnessing the modularity of the developed synthesis route. Investigations along these lines are currently in progress in this laboratory.

## Conflict of interest

The authors declare no conflict of interest.

## Supporting information

As a service to our authors and readers, this journal provides supporting information supplied by the authors. Such materials are peer reviewed and may be re‐organized for online delivery, but are not copy‐edited or typeset. Technical support issues arising from supporting information (other than missing files) should be addressed to the authors.

Supplementary
