# Supplementary material for: Molybdenum Alkylidyne Complexes with Tripodal Silanolate Ligands: The Next Generation of Alkyne Metathesis Catalysts
Source: Angew Chem Int Ed Engl. 2019 Sep 17;58(44):15690–6. doi: 10.1002/anie.201908571 (PMC6856820; doi:10.1002/anie.201908571)

Angewandte Chemie
Eine Zeitschrift der Gesellschaft Deutscher Chemiker

Supporting Information

Molybdenum Alkylidyne Complexes with Tripodal Silanolate Ligands: The Next Generation of Alkyne Metathesis Catalysts

*Julius Hillenbrand, Markus Leutzsch, and Alois Fürstner**

anie_201908571_sm_miscellaneous_information.pdf

SUPPORTING CRYSTALLOGRAPHIC INFORMATION

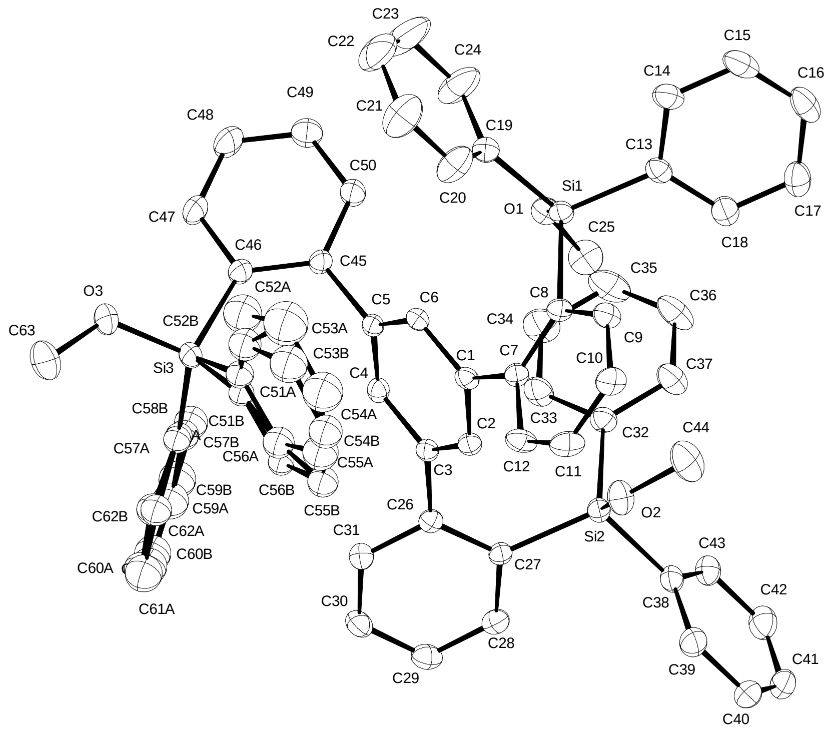

Figure S-1. Structure of compound **12a** in the solid state showing the disorder of one of the Ph_2(MeO)Si- groups over two positions

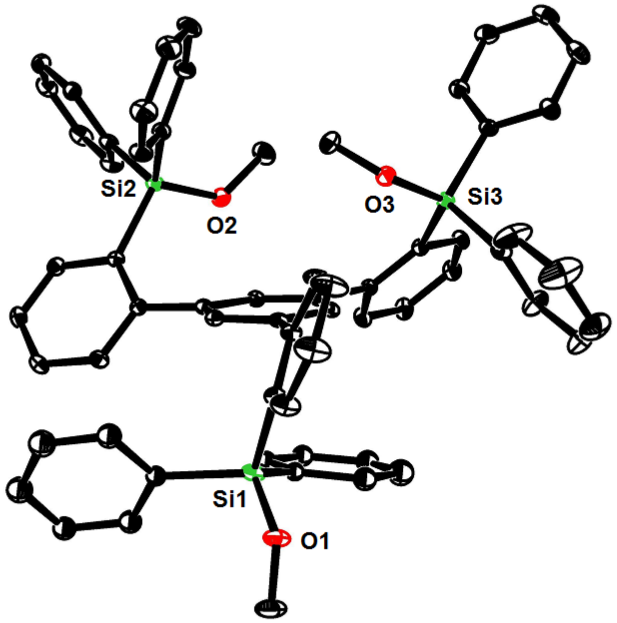

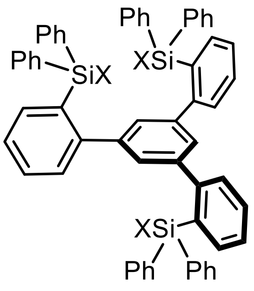

12a (X = OMe)

Figure S-2. Projection of compound **12a** in the solid state (disorder not shown) revealing the different orientation of its silylether groups

X-ray Crystal Structure Analysis of Compound 12a: $C_{63}H_{54}O_3Si_3$, Mr = 943.33 g · mol^{-1}, colorless prism, crystal size 0.124 x 0.095 x 0.041 mm^3, triclinic, space group $P\bar{1}$, a = 9.2600(5) Å, b = 11.8325(7) Å, c = 25.7130(14) Å, α = 82.152(3)°, β = 80.757(2)°, γ = 85.908(3)°, V = 2751.2(3) Å^3, T = 150(2) K, Z = 2, D_{calc} = 1.139 g · cm^3, λ = 0.71073 Å, μ(*Mo-K*α) = 0.130 mm^{-1}, Gaussian absorption correction (T_{min} = 0.99, T_{max} = 1.00), Bruker-AXS Kappa Mach3 APEX-II diffractometer, 0.809 < Θ < 31.351°, 92135 measured reflections, 17920 independent reflections, 12594 reflections with $I > 2\sigma(I)$, R_{int} = 0.0559.

The structure was solved by direct methods and refined by full-matrix least-squares against F^2 to R_1 = 0.077 [$I > 2\sigma(I)$], wR_2 = 0.210, 613 parameters. The H atoms were refined using a riding model, S = 1.081, residual electron density 1.2 (0.10 Å from H34)/ –0.6 (0.26 Å from H24) e · Å^{-3}. **CCDC- 1938115**.

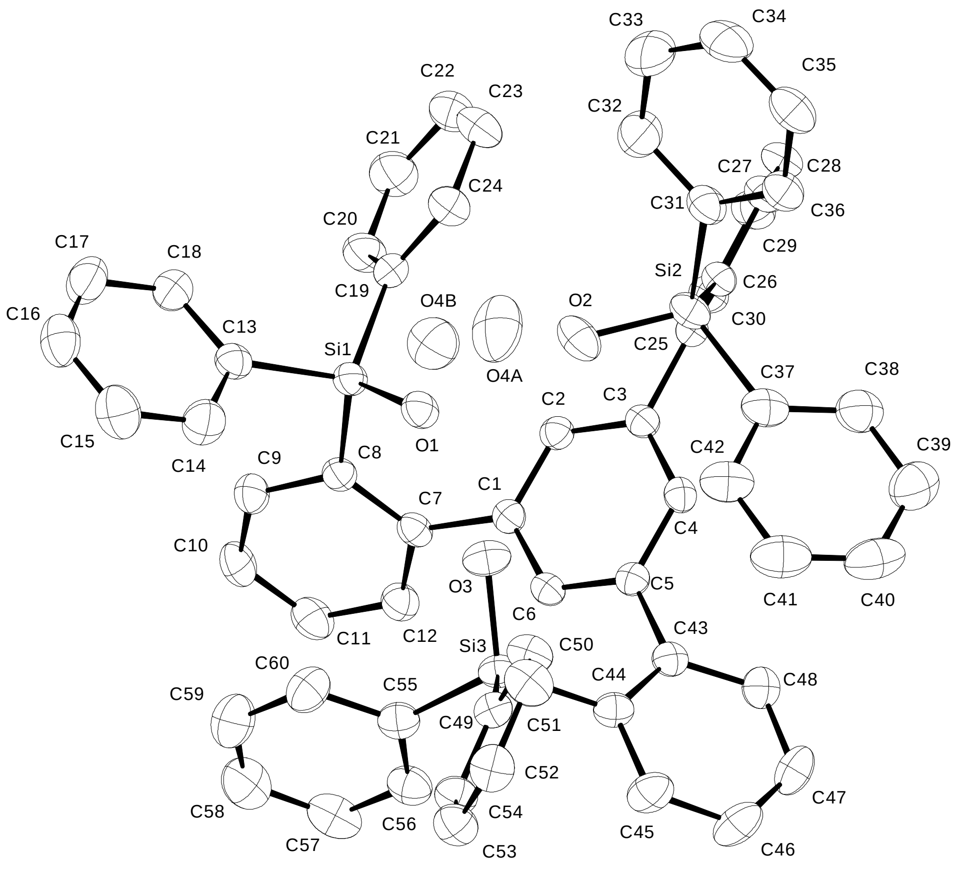

Figure S-3. Structure of the tripodal silanol ligand **13a**·H_2O in the solid state, showing the disorder of the co-crystallized water molecule over two positions

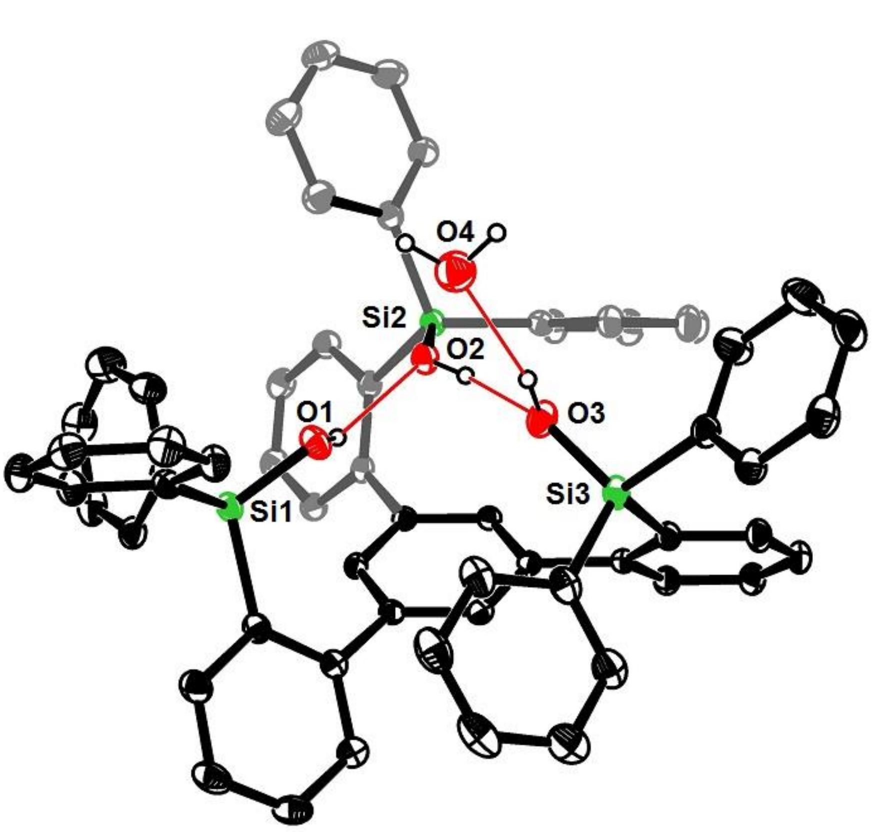

Figure S-4. Structure of the tripodal silanol ligand **13a**·H_2O in the solid state, showing the hydrogen bonding network of the Si–OH groups and the co-crystallized water molecule

X-ray Crystal Structure Analysis of Compound 13a·H_2O: $C_{60}H_{50}O_4Si_3$, Mr = 919.27 g · mol^{-1}, colorless plate, crystal size 0.371 x 0.205 x 0.100 mm^3, triclinic, space group $P\bar{1}$, a = 13.2599(5) Å, b = 13.9312(5) Å, c = 15.4657(5) Å, α = 95.490(2)°, β = 100.576(2)°, γ = 116.665(2)°, V = 2457.62(16) Å^3, T = 150(2) K, Z = 2, D_{calc} = 1.242 g · cm^3, λ = 1.54178 Å, $\mu(Cu\text{-}K\alpha)$ = 1.266 mm^{-1}, Gaussian absorption correction (T_{min} = 0.78, T_{max} = 0.91), Bruker AXS X8 Proteum diffractometer, 2.968 < Θ < 72.366°, 91024 measured reflections, 9135 independent reflections, 7824 reflections with $I > 2\sigma(I)$, R_{int} = 0.0409.

The structure was solved by direct methods and refined by full-matrix least-squares against F^2 to R_1 = 0.041 [$I > 2\sigma(I)$], wR_2 = 0.106, 623 parameters. The H atoms were refined using a riding model, S = 1.092, residual electron density 0.4 (0.92 Å from C37)/–0.4 (0.69 Å from Si2) e · Å^{-3}. **CCDC- 1938114**.

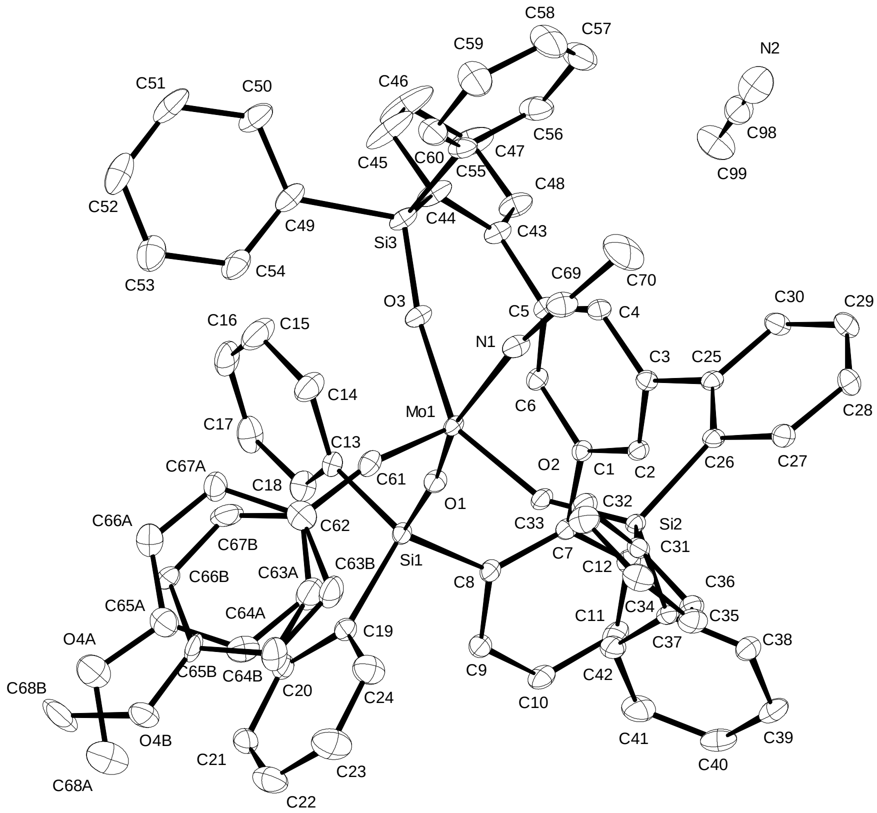

Figure S-5. Structure of complex **16a·MeCN** in the solid state

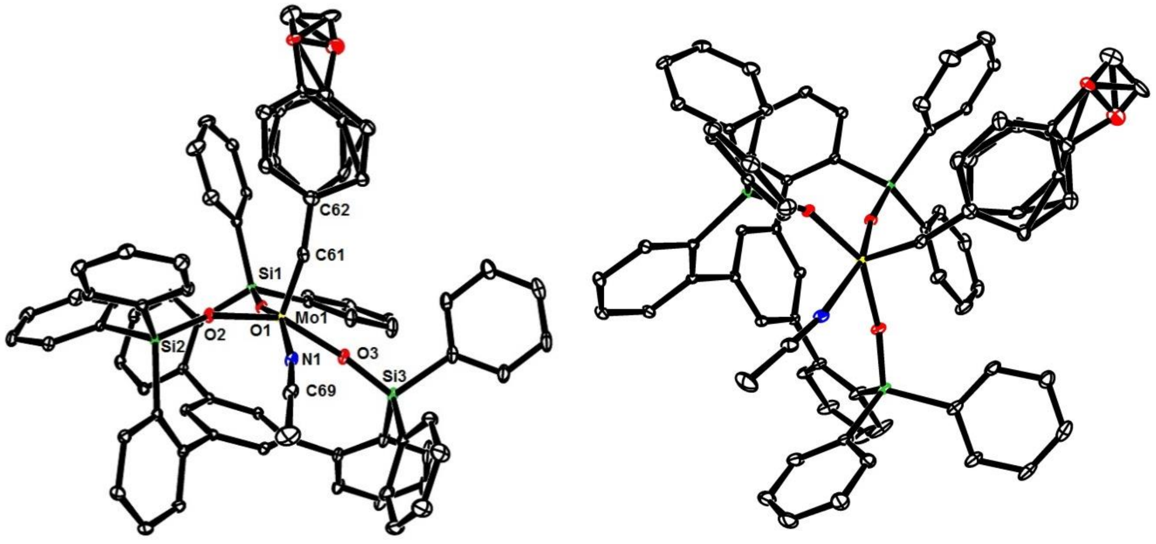

Figure S-6. Structure of complex **16a·MeCN** in two different orientations; color code: Mo = yellow, Si = green, O = red

X-ray Crystal Structure Analysis of Complex 16a·MeCN: $C_{72}H_{58}MoN_2O_4Si_3$, *Mr* = 1195.41 g · mol^{-1}, blue needle, crystal size 0.130 x 0.049 x 0.031 mm^3, monoclinic, space group $P2_1/c$, *a* = 17.6003(8) Å, *b* = 13.1720(5) Å, *c* = 25.0014(11) Å, β = 95.230(2)°, *V* = 5772.0(4) Å^3, *T* = 100(2) K, *Z* = 4, D_{calc} = 1.376 g · cm^3, λ = 0.71073 Å, μ(*Mo-K*α) = 0.344 mm^{-1}, Gaussian absorption correction (T_{min} = 0.97, T_{max} = 0.99), Bruker-AXS Kappa Mach3 APEX-II diffractometer, 1.162 < Θ < 33.381°, 227517 measured reflections, 22353 independent reflections, 15858 reflections with $I > 2\sigma(I)$, R_{int} = 0.0885.

The structure was solved by direct methods and refined by full-matrix least-squares against F^2 to R_1 = 0.045 [$I > 2\sigma(I)$], wR_2 = 0.109, 806 parameters. The H atoms were refined using a riding model, *S* = 1.034, residual electron density 1.2 (0.88 Å from Mo1)/–1.1 (0.63 Å from Mo1) e · Å^{-3}. **CCDC- 1938116**.

General. Unless stated otherwise, all reactions were carried out under Ar in flame-dried glassware. The solvents used were purified by distillation over the drying agents indicated and were transferred under Ar: THF, Et_2O, *n*-pentane, toluene (Na/K); CH_2Cl_2, MeCN (CaH_2).

The molecular sieves used in this investigation were dried for 24 h at 150°C (sand bath) under vacuum prior to use and were stored and transferred under argon atmosphere.

IR: Spectrum One (Perkin-Elmer) spectrometer, wavenumbers ($\tilde{\nu}$) in cm^{-1}. MS (EI): Finnigan MAT 8200 (70 eV), ESI-MS: ESQ 3000 (Bruker), accurate mass determinations: Bruker APEX III FT-MS (7 T magnet) or Mat 95 (Finnigan). Melting points: Buchi melting point apparatus B-540 (corrected). All commercially available compounds (Fluka, Lancaster, Aldrich) were used as received, unless stated otherwise.

Flash chromatography on silica gel (FC): Merck silica gel 60 (230–400 mesh).

Elemental Analyses: Microanalytic Laboratory Kolbe, Mülheim/Ruhr.

NMR: Spectra were recorded on Bruker AVIIIHD3 00, AVIII 400 or AVIII 600 spectrometer in the solvents indicated; chemical shifts (δ) are given in ppm relative to TMS, coupling constants (*J*) in Hz. The solvent signals were used as references and the chemical shifts converted to the TMS scale ($CDCl_3$: $\delta_C \equiv 77.0$ ppm; residual $CHCl_3$ in $CDCl_3$: $\delta_H \equiv 7.26$ ppm; CD_2Cl_2: $\delta_C \equiv 53.8$ ppm; residual $CDHCl_2$: $\delta_H \equiv 5.32$ ppm; [D_8]-toluene: $\delta_C \equiv 20.7$ ppm; residual $D_5C_6CD_2H$: $\delta_H = 2.09$ ppm); unless stated otherwise, all spectra were recorded at 25°C.

^{95}Mo NMR spectra were acquired by the aring pulse sequence to minimize acoustic ringing from the NMR probe. The $\pi/2$ pulse was calibrated against Na_2MoO_4 (2 M in D_2O) and had a typical length of 22.5 µs. Chemical shifts were referenced indirectly to the ^{1}H chemical shift of the solvent. (Ref: R. K. Harris, E. D. Becker, S. M. Cabral de Menezes, P. Granger, R. E. Hoffman, K. W. Zilm, *Pure Appl. Chem.* **2008**, *80*, 59–84).

Diffusion coefficients were obtained from a double stimulated echo sequence with bipolar gradient pulses, convection compensation, longitudinal eddy current delay (LED) and three spoiler gradients (Bruker sequence: dstebpgp3s). The gradient pulse strength G was increased in increments from 2% to 98% of the maximum G_{max} with a squared gradient ramp in 60 steps. The diffusion time (Δ) used was 71 ms and the length of a gradient pulse gradient pulse (δ/2) of the encoding gradient was 1.3 ms. The maximum gradient strength G_{max} of the NMR probe (PA BBO 400S1 BBF-H-D-05 Z PLUS) was 53.5 G·cm^{-1}. Diffusion coefficients were obtained by averaging three diffusion coefficients obtained from fitting the signal decay of three different resonance integrals to the Stejskal-Tanner equation (I) in the Bruker TOPSPIN T1T2 relaxation module.

$$I(G) = I_0 e^{-D(\gamma G \delta)^2(\Delta - \delta/3)} \quad \text{(I)}$$

Diffusion values were predicted using an EXCEL spreadsheet Stokes−Einstein Gierer-Wirtz Estimation (SEGWE) method (Ref: R. Evans, G. Dal Poggetto, M. Nilsson, G. A. Morris, *Anal. Chem*. **2018**, *90*, 3987–3994).

Preparation of the Ligands

1,3,4-Tris-2'-bromophenylbenzene (11).[1] A two-necked, round-bottomed flask was equipped with a

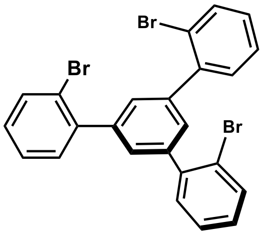

magnetic stir bar, a reflux condenser and a gas inlet connected to an argon/vacuum manifold. The flame-dried flask was filled with argon and charged with 2-bromoacetophenone (**10**) (5.00 g, 25.1 mmol). Trifluoromethanesulfonic acid (0.22 mL, 2.51 mmol) was added dropwise to the stirred neat substrate at 23°C. Once the addition was complete, the mixture was stirred at 130°C for 12 h. The resulting black mixture was allowed to cool to ambient temperature, the reaction was quenched by adding water (20 mL) and the solution was extracted with CH_2Cl_2 (3 x 20 mL). The combined organic layers were washed with water, dried over $MgSO_4$, filtered and concentrated *in vacuo*. The residue was purified by flash chromatography on silica gel (hexanes/CH_2Cl_2 6:1) to give the title compound as a yellow solid (2.69 g, 59%). ^{1}H NMR (400 MHz, $CDCl_3$): δ = 7.69 (dd, *J* = 8.0, 1.2 Hz, 3H), 7.50 (s, 3H), 7.46 (dd, *J* = 7.6, 1.7 Hz, 3H), 7.38 (td, *J* = 7.5, 1.2 Hz, 3H), 7.22 (ddd, *J* = 8.0, 7.4, 1.8 Hz, 3H). ^{13}C NMR (100 MHz, $CDCl_3$): δ = 142.1, 140.6, 133.4, 131.7, 129.8, 129.1, 127.6, 122.8. The analytical and spectroscopic data are in agreement with those reported in the literature.[1]

(5'-(2-(Methoxydiphenylsilyl)phenyl)-[1,1':3',1''-terphenyl]-2,2''-diyl)bis(methoxydiphenylsilane) (12a). A

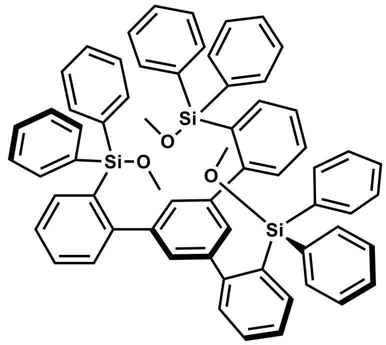

three-necked, round-bottomed flask was equipped with a magnetic stir bar and a gas inlet connected to an argon/vacuum manifold. The flame-dried flask was filled with argon and charged with 1,3,4-tris-2'-bromophenylbenzene (**11**) (2.02 g, 3.72 mmol, 1.00 equiv). Diethyl ether (80 mL) was added and the stirred suspension was cooled to −125°C (bath temperature, pentane/liquid nitrogen). A solution of *tert*-butyllithium (13.5 mL, 22.9 mmol, 1.7 M in pentane, 6.15 equiv) was added over 10 min while the suspension was stirring. The reaction mixture was allowed to warm to ambient temperature and stirring was continued for 1.5 h. The obtained brown suspension was again cooled to −125°C before a solution of diphenyldimethoxysilan (1.94 mL, 8.29 mmol) in diethyl ether (30 mL) was added dropwise over 15 min. The cooling bath was removed and the mixture stirred for 12 h at ambient temperature. The reaction was carefully quenched by adding water and the resulting mixture was transferred into a separation funnel. The organic phase was separated and the aqueous solution was extracted with CH_2Cl_2 (3 x 50 mL). The combined organic layers were dried over $MgSO_4$, filtered and concentrated *in vacuo*. Hexanes (50 mL) was added to precipitate the title compound as a white solid (2.60 g, 74%). Colorless crystals suitable for single-crystal X-ray diffraction were grown by storing a concentrated toluene solution at 5°C for three days. M. p. = 170–173°C. ^{1}H NMR (500 MHz, $CDCl_3$): δ = 7.62 (dd, *J* = 7.4, 1.4 Hz, 3H), 7.38 (td, *J* = 7.5, 1.5 Hz, 3H), 7.35 (dd, *J* = 7.6, 1.3 Hz, 12H), 7.30 (td, *J* = 7.4, 1.3 Hz, 3H), 7.24 (tt, *J* = 7.4, 1.4 Hz, 6H), 7.14 (t, *J* = 7.5, 7.1 Hz, 12H), 6.98 (dd, *J* = 7.6, 1.3 Hz, 3H),

6.95 (s, 3H), 3.01 (s, 9H). ^{13}C NMR (126 MHz, $CDCl_3$): δ = 149.6, 141.5, 137.3, 135.2, 135.1, 131.6, 130.9, 129.6, 129.4, 129.2, 127.5, 125.7, 50.9. ^{29}Si NMR (99 MHz, $CDCl_3$): δ = −11.6. IR (film): $\tilde{\nu}$ 3045, 2930, 2830, 1582, 1557, 1471, 1427, 1409, 1263, 1183, 1108, 1082, 998, 877, 830, 760, 735, 697, 621, 499, 477, 411 cm^{-1}. HRMS-ESI (*m/z*): calcd. for $C_{36}H_{54}O_3Si_3Na^+$ $[M+Na]^+$, 965.3273; found, 965.3286.

Ligand 13a. A two-necked, round-bottomed flask was equipped with a magnetic stir bar and a dropping funnel. The flask was charged with silane **12a** (2.09 g, 2.22 mmol, 1.00 equiv) and THF (50 mL) and the resulting solution was cooled to 0°C. Concentrated aqueous HCl (ca. 12 M, 11.9 mL, 143 mmol, 90 equiv.) was added dropwise to the stirred solution. The mixtures was allowed to warm to ambient temperature and stirring was continued for 1 h. The mixture was cooled to 0°C and carefully neutralized by dropwise addition of saturated aqueous $NaHCO_3$ solution. The organic phase was separated and the aqueous solution was extracted with CH_2Cl_2 (3 x 50 mL). The combined organic layers were washed with water (200 mL), dried over $MgSO_4$, filtered and concentrated *in vacuo* to give the title compound as a white solid (1.98 g, 99%). Colorless crystals suitable for single-crystal X-ray diffraction were grown by storing a concentrated toluene solution at 5°C for three days. M. p. = 225–228°C. ^{1}H NMR (400 MHz, $CDCl_3$): δ = 7.55 – 7.51 (m, 12H), 7.48 – 7.43 (m, 3H), 7.30 – 7.22 (m, 12H), 7.21 – 7.13 (m, 12H), 6.92 – 6.87 (m, 6H), 4.36 (s, 3H). ^{13}C NMR (126 MHz, $CDCl_3$): δ = 149.0, 143.8, 137.5, 137.0, 134.6, 133.9, 130.1, 129.7, 129.4, 128.6, 127.9, 126.0. IR (film): $\tilde{\nu}$ 3046, 1582, 1471, 1427, 1263, 1204, 1112, 1087, 997, 900, 824, 762, 735, 698, 622, 471, 411 cm^{-1}. HRMS-ESI (*m/z*): calcd. for $C_{60}H_{48}O_3Si_3Na^+$ $[M+Na]^+$, 923.2804; found, 923.2816.

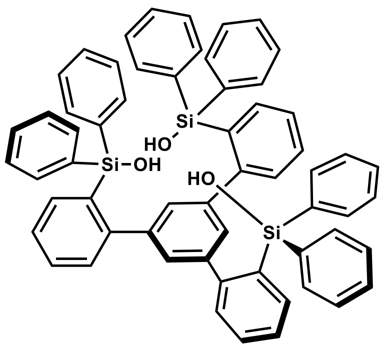

Dimethoxybis(4-methoxyphenyl)silane (S1). A two-necked, round-bottomed flask was equipped with a magnetic stir bar, a reflux condenser and a gas inlet connected to an argon/vacuum manifold. The flame-dried flask was charged with magnesium turnings (851 mg, 35.0 mmol, 5.00 equiv) and THF (6 mL) under argon atmosphere. 1,2-Dibromoethane (3 drops) was added dropwise to the stirred mixture, which was quickly heated up to 60°C for 5 min to activate the magnesium. After reaching ambient temperature, 4-bromoansiole (2.19 mL, 17.5 mmol, 2.5 equiv.) was added dropwise and the resulting green-grey suspension was stirred for 1h at 23°C until full conversion was indicated by GC-MS analysis.

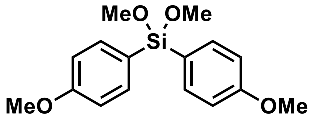

A three-necked, round-bottomed flask was equipped with a magnetic stir bar, a dropping funnel and a gas inlet connected to an argon/vacuum manifold. The flame-dried flask was filled with argon and charged with tetramethylorthosilicate (1.03 mL, 7.00 mmol, 1.00 equiv). THF (23 mL) was added and the colorless solution was cooled to 0°C. The solution of the freshly prepared Grignard reagent was diluted with THF (8 mL) and

transferred into the dropping funnel, using additional THF (8 mL) to rinse the flask. This solution was added dropwise to the cooled solution of the orthosilicate. Once the addition was complete, the mixture was allowed to warm to ambient temperature and stirring was continued for 4 h. The mixture was poured into a separation funnel charged with *n*-pentane (50 mL) and was washed with water (3 x 50 mL).The organic layer was dried over $MgSO_4$, filtered and concentrated *in vacuo*. The residue was purified by flash chromatography on silica gel (hexanes/EtOAc 98:2) to give the title compound as a colorless oil (1.05 g, 49%). ^{1}H NMR (400 MHz, C_6D_6): δ = 7.85 – 7.74 (m, 4H), 6.91 – 6.82 (m, 4H), 3.54 (s, 6H), 3.27 (d, *J* = 0.9 Hz, 6H). ^{13}C NMR (100 MHz, C_6D_6): δ = 162.1, 137.1, 124.3, 114.1, 54.6, 50.6. IR (film): $\tilde{\nu}$ 2937, 2836, 1593, 1565, 1503, 1461, 1399, 1309, 1279, 1245, 1179, 1123, 1069, 1028, 737, 719, 658, 631, 601, 529, 508, 444 cm^{-1}. HRMS-ESI (*m/z*): calcd. for $C_{16}H_{20}O_4SiNa^+$ [M+Na]$^+$, 327.1023; found, 327.1023.

(5'-(2-(Methoxybis(4-methoxyphenyl)silyl)phenyl)-[1,1':3',1''-terphenyl]-2,2''-diyl)bis(methoxybis(4-methoxyphenyl)silane) (12b). A three-necked, round-bottomed flask was equipped with a magnetic stir bar and a gas inlet connected to an argon/vacuum manifold. The flame-dried flask was filled with argon and charged with compound **11** (1.63 g, 3.00 mmol, 1.00 equiv). Diethyl ether (60 mL) was added and the stirred suspension was cooled to −125°C (bath temperature, pentane/liquid nitrogen). A solution of *tert*-butyllithium (10.9 mL, 18.5 mmol, 1.7 M in pentane, 6.15 equiv) was added over 10 min while the suspension was stirring. The mixture was allowed to warm to ambient temperature and stirring was continued for 1.5 h. The obtained brown suspension was again cooled to −125°C before a solution of silane **S1** (4.30 g, 14.1 mmol, 4.7 equiv.) in diethyl ether (5 mL) was added dropwise over 15 min. The mixture was then stirred at ambient temperature for 8 h. The reaction was carefully quenched by adding water and the resulting mixture was transferred into a separation funnel. The organic phase was separated and the aqueous solution was extracted with CH_2Cl_2 (3 x 50 mL). The combined organic layers were dried over $MgSO_4$, filtered and concentrated *in vacuo*. The residue was purified by preparatory high-performance liquid chromatography with MeCN as the eluent to afford the title compound as a white solid (400 mg, 12%). M. p. = 97–100°C. ^{1}H NMR (400 MHz, $CDCl_3$): δ = 7.70 – 7.62 (m, 3H), 7.37 (td, *J* = 7.5, 1.6 Hz, 3H), 7.31 (dd, *J* = 7.4, 1.3 Hz, 3H), 7.27 – 7.23 (m, 12H), 6.99 – 6.95 (m, 3H), 6.92 (s, 3H), 6.70 – 6.64 (m, 12H), 3.71 (s, 18H), 3.00 (s, 9H). ^{13}C NMR (126 MHz, $CDCl_3$): δ = 160.7, 149.8, 141.6, 137.3, 136.9, 132.6, 131.0, 129.6, 129.3, 126.4, 125.7, 113.3, 55.0, 50.8. ^{29}Si NMR (99 MHz, $CDCl_3$): δ = −11.3. IR (film): $\tilde{\nu}$ 2932, 2832, 1592, 1562, 1501, 1461, 1440, 1398, 1276, 1244, 1178, 1027, 815, 797, 719, 689, 649, 629, 609, 531, 463 cm^{-1}. HRMS-ESI (*m/z*): calcd. for

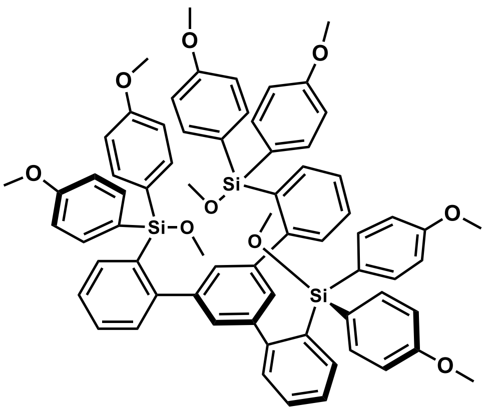

$C_{69}H_{66}O_9Si_3Na^+$ [M+Na]$^+$, 1145.3907; found, 1145.3909.

Ligand 13b. A two-necked, round-bottomed flask was equipped with a magnetic stir bar and a dropping funnel. The flask was charged with silyl ether **12b** (400 mg, 2.22 mmol, 1.00 equiv) and THF (7.6 mL). The resulting solution was cooled to 0°C before concentrated aqueous HCl (ca. 12 M, 2.60 mL, 31.2 mmol, 90 equiv.) was added dropwise. The mixture was allowed to warm to ambient temperature and stirring was continued for 1 h. The reaction mixture was cooled to 0°C before it was carefully neutralized by adding saturated aqueous $NaHCO_3$ solution dropwise to the mixture. The organic phase was separated and the aqueous solution was extracted with CH_2Cl_2 (3 x 10 mL). The combined organic layers were washed with water (50 mL), dried over $MgSO_4$, filtered and concentrated *in vacuo* to give the title compound as a white solid (380 mg, 99%). M. p. = 250–253°C. ^{1}H NMR (500 MHz, $CDCl_3$): δ = 7.49 – 7.45 (m, 3H), 7.45 – 7.42 (m, 12H), 7.28 – 7.25 (m, 6H), 6.92 (d, *J* = 7.0 Hz, 6H), 6.72 – 6.68 (m, 12H), 3.74 (s, 18H). ^{13}C NMR (126 MHz, $CDCl_3$): δ = 160.7, 148.9, 143.5, 137.3, 136.0, 134.7, 129.7, 129.3, 128.5, 128.3, 125.7, 113.4, 54.9. ^{29}Si NMR (99 MHz, $CDCl_3$): δ = −11.6. IR (film): $\tilde{\nu}$ 1592, 1562, 1501, 1437, 1276, 1244, 1180, 1111, 1030, 897, 811, 795, 764, 722, 690, 650, 628, 612, 529, 464, 428, 410 cm^{-1}. HRMS-ESI (*m/z*): calcd. for $C_{66}H_{60}O_9Si_3Na^+$ [M+Na]$^+$, 1103.3437; found, 1103.3435.

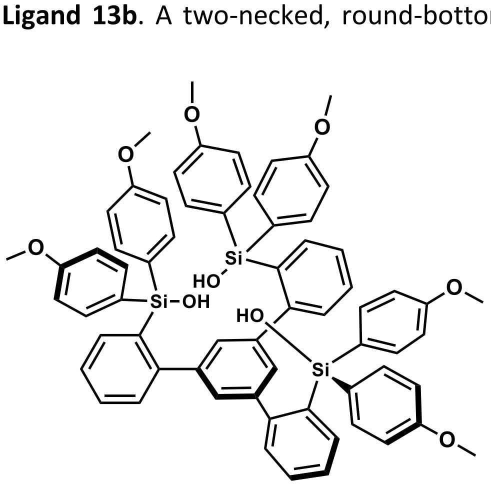

Preparation of the Complexes

Complex 1: Optimized Procedure.[3] A 100 mL, three neck round-bottom flask was equipped with a magnetic stirring bar, a pressure-equalizing dropping funnel fitted with a rubber stopper and a gas inlet connected to an argon/vacuum manifold. The flask was loaded with [Mo(≡CAr)Br_3(dme)] (Ar = 4-Methoxyphenyl, **14**) (570 mg, 1,05 mmol) and THF (15 mL). The stirring brown solution was cooled to 0 °C before a solution of sodium triphenylsilanolate (936 mg, 3,14 mmol) in THF (25 mL) was added via the addition funnel over 45 min. When the addition was complete, the cooling bath was removed and stirring continued for an additional 1 h. *n*-Pentane (40 mL) was added and the mixture filtered through a pad of compressed Celite (2 cm), rinsing with THF/*n*-pentane (1:1 *v/v*, 2 x 10 mL). The brown filtrate was concentrated to dryness and the residue dried under high vacuum (10^{-3} mbar) for 1 h. The resulting yellow-brown foam was triturated with *n*-pentane (25 mL), resulting in the formation of a brown tarry semisolid. This mixture was sonicated for 30 min and then vigorously stirred for 30 min, during which time much of the brown material turned into a yellow powder. Diethyl ether (5 mL) was added and the mixture was again sonicated

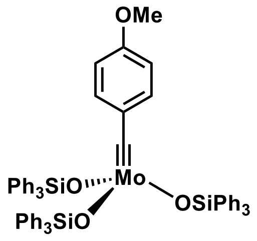

until no brown solid remained. The yellow solid material was collected by filtration, washing with *n*-pentane/diethyl ether (10 mL, 5:1) and finally diethyl ether (5 mL). The resulting material was dried under high vacuum to give the title complex as a yellow powder (860 mg, 79 %). ^{1}H NMR (400 MHz, $C_6D_5CD_3$): δ = 7.79 – 7.72 (m, 18H), 7.22 – 7.15 (m, 9H), 7.10 (t, *J* = 7.4 Hz, 18H), 6.18 – 6.13 (m, 2H), 5.57 – 5.49 (m, 2H), 3.19 (s, 3H). ^{13}C NMR (101 MHz, $C_6D_5CD_3$): δ = 300.5, 158.7, 140.3, 136.4, 135.8, 131.1, 130.0, 128.1, 112.0, 54.4. ^{29}Si NMR (79 MHz, C_6D_6): δ = −8.0. ^{95}Mo NMR (26 MHz, 23°C, $C_6D_5CD_3$): δ = 385.8. ^{95}Mo NMR (26 MHz, 60°C, $C_6D_5CD_3$): δ = 397.6.

Complex 15. A 250 mL Schlenk flask was equipped with a magnetic stir bar and was flame dried under vaccum.

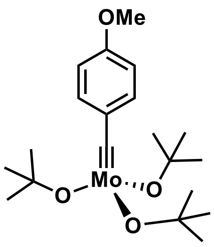

The flask was filled with argon and charged with Mo(≡CAr)Br_3(dme) (Ar = 4-Methoxyphenyl, **14**) (3.02 g, 5.55 mmol, 1.00 equiv.) and THF (62 mL). A solution of NaO*t*Bu (1.62 g, 16.5 mmol, 2.98. equiv) in THF (15 mL) was added dropwise at 23°C to the stirred brown solution. Stirring was continued for 14 h at ambient temperature before the solvent was removed *in vacuo* to obtain a dark brown solid. A second, flame dried 250 mL Schlenk flask was equipped with a magnetic stir bar and a Celite® (2 cm) packed argon frit. The dark brown solid was suspended in *n*-pentane (4 x 20 mL) and was filtered over the Celite pad. The resulting filtrate was concentrated and the residue was dried under vacuum (10^{-3} mbar) to give the title complex as a brown solid (2.00 g, 83%) free of any residual THF. ^{1}H NMR (400 MHz, C_6D_6): δ = 7.49 – 7.43 (m, 2H), 6.70 – 6.59 (m, 2H), 3.24 (s, 3H), 1.54 (s, 27H). ^{13}C NMR (101 MHz, C_6D_6): δ = 276.5, 158.7, 140.9, 131.3, 113.7, 80.0, 54.8, 32.9. The spectroscopic data are in close correspondence to those reported for Mo(≡CPh)(O*t*Bu)$_3$.[2]

Complex [16a]$_2$. A 250 mL Schlenk flask was equipped with a magnetic stir bar and was flame dried under

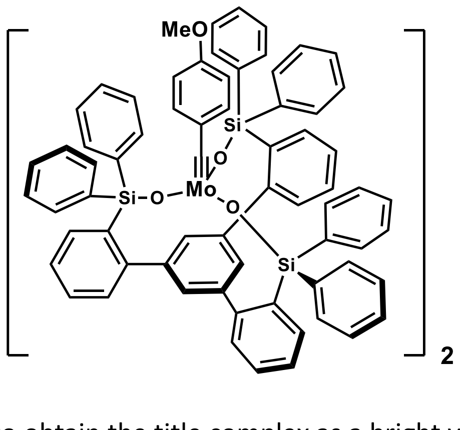

vacuum. The flask was filled with argon, charged with ligand **13a** (968 mg, 1.07 mmol, 1.00 equiv), which was azeotropically dried with benzene (3 x 5 mL) to remove residual water. Toluene (81 mL) was added and the mixture was vigorously stirred for 10 min to obtain a clear solution. A solution of complex **15** (467 mg, 1.07 mmol, 1.00 equiv.) in toluene (16 mL) was added dropwise to the vigorously stirred solution at 23°C. After stirring at ambient temperature for an additional 1 h, the solvent was removed *in vacuo* to obtain the title complex as a bright yellow powder (1.14 g, 95%). ^{1}H NMR (400 MHz, C_6D_6): δ = 8.56 – 8.46 (m, 2H), 8.34 (d, *J* = 7.3 Hz, 1H), 7.99 – 7.87 (m, 2H), 7.86 – 7.72 (m, 3H), 7.66 (dt, *J* = 13.7, 6.5 Hz, 3H), 7.60 – 7.48 (m, 3H), 7.42 (s, 1H), 7.05 (d, *J* = 6.0 Hz, 1H), 7.00 (d, *J* = 6.1 Hz, 1H), 6.96 (s, 1H), 6.87 (s, 3H), 6.71 (d, *J* = 7.5 Hz, 1H), 6.61 (s, 1H), 6.38 (s, 2H), 6.07 (s, 2H), 6.00 – 5.91 (m, 2H), 5.06 – 4.93 (m, 2H), 3.08 (s, 3H). ^{13}C NMR (101 MHz, C_6D_6): δ = 297.8, 158.0, 150.4, 149.9, 149.3, 143.7, 143.5, 142.6, 139.3, 139.2, 138.4, 130.3,

111.6, 54.1; ^{1}H-^{13}C HMBC NMR ($C_6D_5CD_3$): δ = 297.6 (alkylidyne). ^{29}Si NMR (99 MHz, C_6D_6): δ = −6.8. ^{1}H-DOSY NMR ($C_6D_5CD_3$): $D_{exp.}$= 3.32·10^{-10} m^2/s^{-1}. Anal. (%) calcd for $C_{136}H_{104}Mo_2O_8Si_6$: C 73.36, H 4.71, Mo 8.62, Si 7.57; found: C 71.10, H 4.92, Mo 8.31, Si 7.51.

In solid form, the complex is only moderately air-sensitive, showing a half-lifetime at ambient temperature of approximately 8 h (Figure S-7).

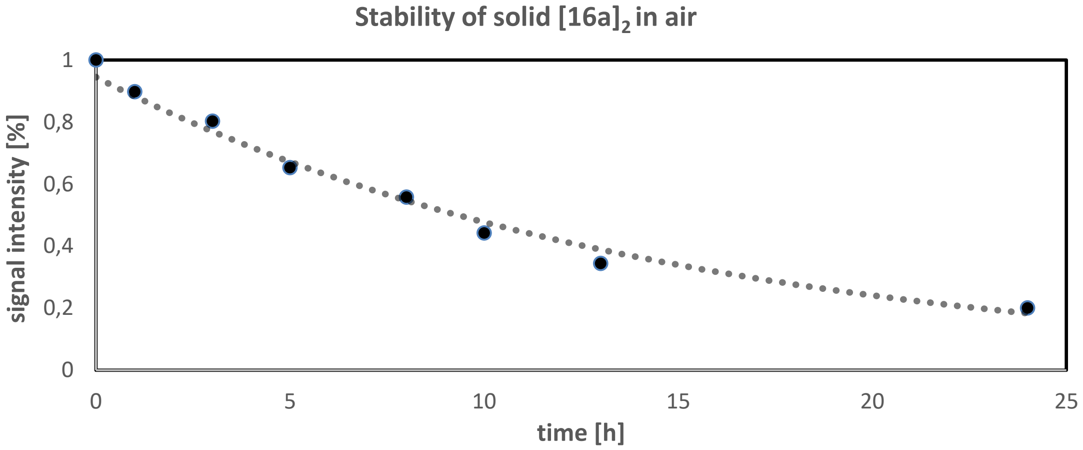

Figure S-7. Stability of solid [**16a**]$_2$ in air at ambient temperature as determined by ^{1}H NMR in C_6D_6; the signals of the *ortho*-protons of the *p*-methoxybenzylidyne group were integrated against the signal of the free ligand. The humidity of the air was ≈ 60%.

Complex 16a. A 10 mL Schlenk flask was equipped with a magnetic stir bar and was flame dried under vacuum.

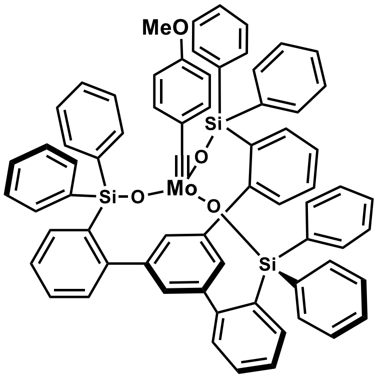

The flask was filled with argon and then charged with complex [**16a**]$_2$ (94.0 mg, 42.2 µmol) and $C_6D_5CD_3$ (2 mL). The resulting yellow suspension was vigorously stirred at 60°C for 1 h to give complex **16a** as an orange solution (quant.). ^{1}H NMR (400 MHz, $C_6D_5CD_3$): δ = 7.86 – 7.77 (m, 12H), 7.78 – 7.69 (m, 3H), 7.25 (s, 3H), 7.13 – 7.00 (m, 24H), 6.87 (dd, *J* = 6.3, 2.5 Hz, 3H), 6.21 (d, *J* = 8.8 Hz, 2H), 6.13 (d, *J* = 8.6 Hz, 2H), 3.05 (s, 3H). ^{13}C NMR (101 MHz, $C_6D_5CD_3$): δ = 310.4, 158.7, 149.1, 143.6, 140.6, 137.7, 137.0, 137.0, 135.0, 134.6, 130.1, 130.0, 129.6, 129.3, 128.9, 128.8, 128.0, 127.7, 125.8, 112.1, 53.9. ^{29}Si NMR (79 MHz, C_6D_6): δ = −9.9. ^{95}Mo NMR (26 MHz, 23°C, $C_6D_5CD_3$): δ = 419.0. ^{95}Mo NMR (26 MHz, 60°C, $C_6D_5CD_3$): δ = 421.8. ^{1}H-DOSY NMR ($C_6D_5CD_3$): $D_{exp.}$ = 5.557·10^{-10} m^2/s^{-2}. HRMS-ESI (*m/z*): calcd. for $C_{68}H_{53}MoO_4Si_3^+$ [M+H]$^+$, 1115.2300; found, 1115.2317; Anal. (%) calcd for $C_{68}H_{52}MoO_4Si_3$: C 73.36, H 4.71; C 71.10, H 4.92.

Complex 16a·MeCN. A 10 mL Schlenk flask was equipped with a magnetic stir bar and was flame dried under vacuum. The flask was filled with argon, charged with complex [**16a**]$_2$ (12.0 mg, 10.8 µmol, 1.00 equiv) and $C_6D_5CD_3$ (1.2 mL) to give a yellow suspension. Acetonitrile (2.8 µL, 53.9 µmol, 5 equiv.) was added to give a slight touch of blue color. The mixture was vigorously stirred at 60°C for 1 h to give the title complex as an orange-blueish solution (quant.). Blue crystals suitable for single-crystal X-ray diffraction were grown by dissolving the solid material (40.0 mg, 35.9 µmol) in benzene (1 mL) and acetonitrile (2 mL); this mixture was layered with *n*-pentane (5 mL) and kept at ambient temperature. ^{1}H NMR (400 MHz, $C_6D_5CD_3$): δ = 8.00 – 7.92 (m, 12H), 7.82 (dd, *J* = 5.7, 3.1 Hz, 3H), 7.43 (s, 3H), 7.03 (ddd, *J* = 14.0, 7.9, 4.8 Hz, 18H), 6.85 – 6.79 (m, 3H), 6.41 – 6.33 (m, 3H), 3.13 (s, 3H), 0.56 (s, 3H). ^{1}H-DOSY NMR ($C_6D_5CD_3$): $D_{exp.} = 5.46 \cdot 10^{-10}$ m^2/s^{-1}. HRMS-ESI (*m/z*): calcd. for $C_{68}H_{53}MoO_4Si_3^+$ [M+H]$^+$, 1115.2300; found, 1115.2317.

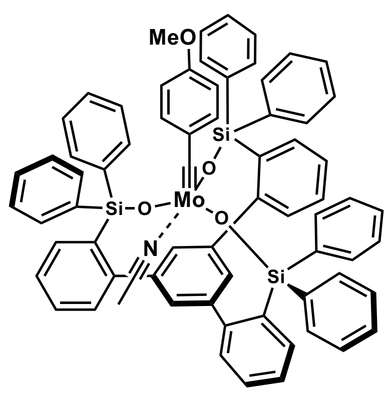

Complex 16b. A 50 mL Schlenk flask was equipped with a magnetic stir bar and was flame dried under vaccum. The flask was filled with argon, charged with ligand **13b** (329 mg, 0.304 mmol, 1.00 equiv) and azeotropically dried with benzene (3 x 5 mL) to remove residual water. Toluene (23 mL) was added and the resulting mixture was vigorously stirred for 10 min to obtain a clear solution. A solution of complex **15** (132 mg, 0.304 mmol, 1.00 equiv.) in toluene (5 mL) was added dropwise and stirring was continued for 1 h at ambient temperature. The solvent was removed *in vacuo* to give a bright yellow powder (299 mg, 76%, mixture of monomer **16b** and dimer [**16b**]$_2$), which was used in the next step.

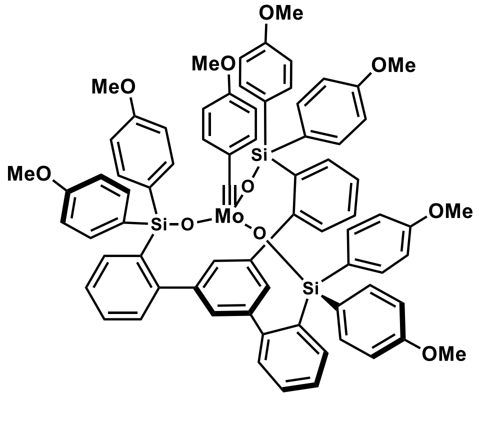

A 10 mL Schlenk flask was equipped with a magnetic stir bar and was flame dried under vacuum. The flask was filled with argon, charged with the crude mixture of [**16b**]$_2$/**16b** (60.0 mg, 46.4 µmol) and $C_6D_5CD_3$ (1 mL). The resulting yellow suspension was vigorously stirred at 60°C for 1 h to give an orange solution containing only complex **16b**. ^{1}H NMR (400 MHz, $C_6D_5CD_3$): δ = 7.85 (dd, *J* = 6.9, 1.9 Hz, 3H), 7.81 – 7.73 (m, 9H), 7.36 (s, 3H), 7.15 (dd, *J* = 7.1, 1.8 Hz, 3H), 7.13 – 7.08 (m, 3H), 6.97 – 6.93 (m, 3H), 6.73 – 6.58 (m, 12H), 6.28 – 6.23 (m, 3H), 6.20 – 6.10 (m, 3H), 3.31 (s, 18H), 3.06 (s, 3H). ^{13}C NMR (101 MHz, $C_6D_5CD_3$): δ = 309.3, 161.0, 158.5, 149.3, 143.6, 140.7, 136.2, 136.1, 130.2, 130.0, 129.4, 129.1, 128.9, 125.6, 113.6, 112.0, 53.9, 53.9. ^{29}Si NMR (79 MHz, C_6D_6): δ = −9.1. ^{95}Mo NMR (26 MHz, 23°C, $C_6D_5CD_3$): δ = 405.2. ^{95}Mo NMR (26 MHz, 60°C, $C_6D_5CD_3$): δ = 414.3. ^{1}H-DOSY NMR

($C_6D_5CD_3$): $D_{exp.}$ = 5.557·10^{-10} m^2/s^{-2}. HRMS-APPI (*m/z*): calcd. for $C_{74}H_{64}MoO_{10}Si_3^+$ [M+H]$^+$, 1294.2856; found, 1294.2862.

DOSY Experiments

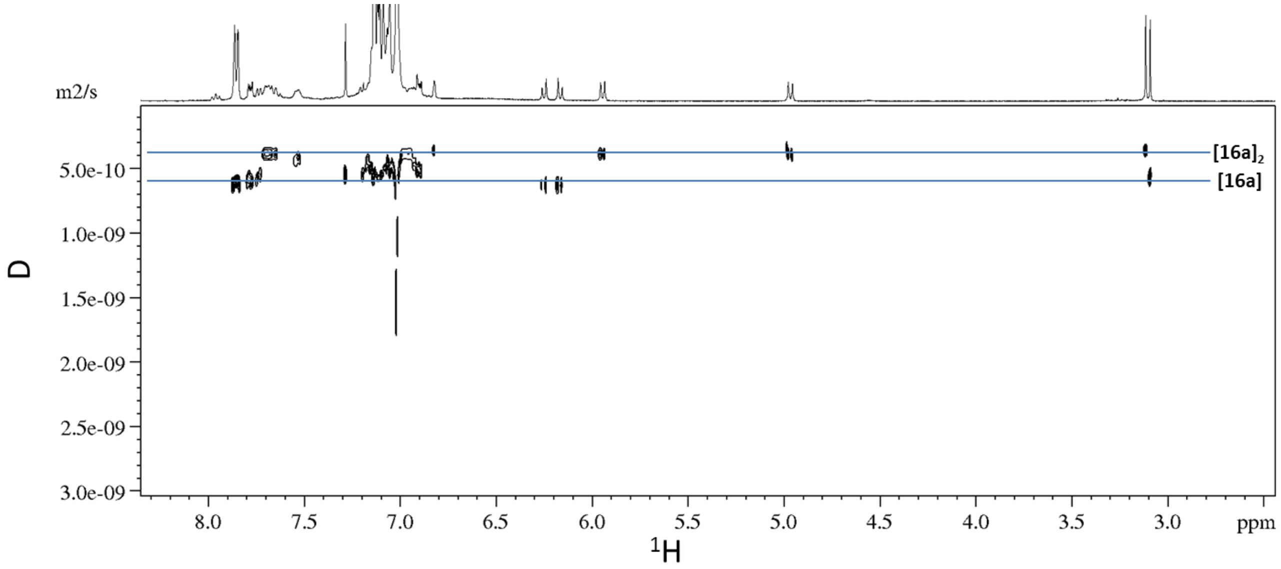

Figure S-8. 2D DOSY plot of a ≈1:1 mixture of [**16a**] and [**16a**]$_2$ showing the different diffusion coefficients.

Table S-1. Measured and predicted diffusion coefficients (D) of the different complexes.

Complex	MW (g·mol^{-1})	$D_{predicted}$ [m^2·s^{-1}]	$D_{exp.}$ [m^2·s^{-1}] [a]	Δ
**16a**	1113.33	5.55·10^{-10} ± 1.6·10^{-10}	5.557·10^{-10}	0.04%
[**16a**]$_2$	2226.66	3.47·10^{-10} ± 1.0·10^{-10}	3.302·10^{-10}	−4.93%
**16a**·MeCN	1154.38	5.43·10^{-10} ± 1.6·10^{-10}	5.458·10^{-10}	0.50%
**16b**	1293.48	5.20·10^{-10} ± 1.5·10^{-10}	4.758·10^{-10}	−8.47%
[**16b**]$_2$	2586.97	3.86·10^{-10} ± 1.1·10^{-10}	2.959·10^{-10}	−23.36%

[a] average of three experiments as described in the section "General"

Alkyne Metathesis Reactions

Table S-2. Comparison of the reactivity of the parent complex **1** and the new podand complex [**16a**]$_2$; the reactions were performed at ambient temperature

Entry	Catalyst (5 mol%)	t (h)	Product	Yield (%)
1	**1**	2	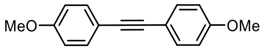	82
2	[**16a**]$_2$	9		92
3	**1**	0.5	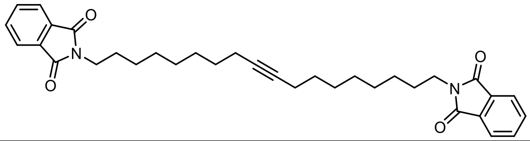	73
4	[**16a**]$_2$	6		89

Representative Procedure for Alkyne Homo-Metathesis. Octadec-9-yne-1,18-diol (S2). Undec-9-yn-1-ol

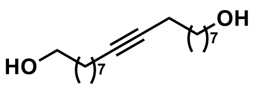

(43.5 mg, 25.0 µmol, 1.00 equiv) was added to a stirred suspension of complex **16a** (13.9 mg, 1.25 µmol, 5 mol%) and powdered molecular sieves 5Å (250 mg) in toluene (1.25 mL) at 23°C under argon atmosphere. The mixture was stirred for 12 h at this temperature before it was filtered through a short pad of Celite® which was carefully rinsed with ethyl acetate (20 mL). The combined filtrates were evaporated and the residue was purified by flash chromatography on silica gel (hexanes/*t*-butyl methyl ether, 6:4 to 1:4) to give the title compound as a white solid (32.2 mg, 91%). ^{1}H NMR (400 MHz, CDCl$_3$): δ = 3.63 (t, *J* = 6.6 Hz, 4H), 2.19 – 2.06 (m, 4H), 1.61 – 1.42 (m, 8H), 1.37 – 1.27 (m, 16H). ^{13}C NMR (100 MHz, CDCl$_3$): δ = 80.4, 63.2, 32.9, 29.5, 29.3, 29.2, 28.9, 25.8, 18.9. IR (film): $\tilde{\nu}$ 3260, 2930, 2851, 1460, 1414, 1052, 1023, 995, 725, 420 cm^{-1}. HRMS-ESI (*m/z*): calcd. for $C_{18}H_{34}O_{32}^{+}$ [M]$^+$, 283.2632; found, 283.2633.

1,2-Bis(4-methoxyphenyl)ethyne (S3). Prepared analogously as a white solid (27.3 mg, 92%). ^{1}H NMR (400

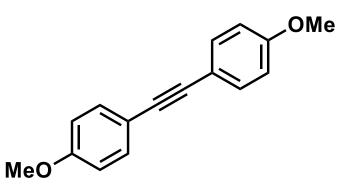

MHz, CDCl$_3$): δ = 7.45 (d, *J* = 8.8 Hz, 3H), 6.87 (d, *J* = 8.8 Hz, 4H), 3.82 (s, 6H). ^{13}C NMR (100 MHz, CDCl$_3$): δ = 159.5, 133.0, 115.9, 114.1, 88.1, 55.4. The analytical and spectroscopic data are in agreement with those reported in the literature.[5]

1,2-Bis(4-(trifluoromethyl)phenyl)ethyne (S4). Prepared analogously, but the reaction was carried out at 60°C; white solid (35.5 mg, 90%). ^{1}H NMR (400 MHz, CDCl$_3$): δ = 7.68 – 7.59 (m,

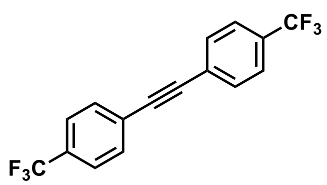

8H). ^{13}C NMR (100 MHz, CDCl$_3$): δ = 132.1, 131.1, 130.8, 130.5, 130.2, 128.0, 126.5, 125.6, 125.5, 125.5, 125.3, 122.6, 119.9, 90.3. ^{19}F NMR (282 MHz, CDCl$_3$): δ = −62.9. The analytical and spectroscopic data are in agreement with those

reported in the literature.[6]

4,4'-(Ethyne-1,2-diyl)dibenzonitrile (S5). Prepared analogously, but the reaction was carried out using complex **16b** at 60°C; white solid (22.0 mg, 77%). ^{1}H NMR (400 MHz, $CDCl_3$): δ = 7.67 (d, *J* = 8.6 Hz, 2H), 7.63 (d, *J* = 8.8 Hz, 2H). ^{13}C NMR (100 MHz, $CDCl_3$): δ = 132.7, 132.6, 127.5, 118.7, 112.9, 92.0. The analytical and spectroscopic data are in agreement with those reported in the literature.[3]

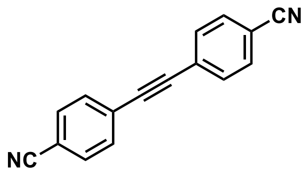

4,4'-(Ethyne-1,2-diyl)diphenol (S6). Prepared analogously, but the reaction was carried out using complex **16b** at 60°C; white solid (24.3 mg, 93%). ^{1}H NMR (400 MHz, CD_3CN): δ = 7.39 – 7.31 (m, 4H), 7.27 (s, 2H), 6.85 – 6.77 (m, 4H). ^{13}C NMR (100 MHz, CD_3CN): δ = 158.1, 133.8, 116.5, 115.6, 88.4. The analytical and spectroscopic data are in agreement with those reported in the literature.[4]

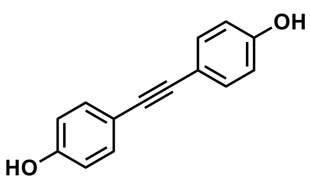

Dimethyl 2,7-diacetamidooct-4-ynedioate (S7). Prepared analogously as a white solid (26.2 mg, 67%). ^{1}H NMR (400 MHz, $CDCl_3$): δ = 7.16 (d, *J* = 9.1 Hz, 2H), 6.57 (d, *J* = 8.5 Hz, 2H), 4.88 (dt, *J* = 9.1, 3.7 Hz, 2H), 4.79 (dt, *J* = 7.8, 3.9 Hz, 2H), 3.80 – 3.76 (m, 13H), 2.64 (dtd, *J* = 17.4, 4.5, 2.8 Hz, 8H), 2.12 – 2.11 (m, 13H). ^{13}C NMR (100 MHz, $CDCl_3$): δ = 172.6, 171.5, 170.4, 170.4, 78.6, 78.0, 52.9, 50.7, 50.4, 23.5, 23.2, 23.2, 22.9. The analytical and spectroscopic data are in agreement with those reported in the literature.[4]

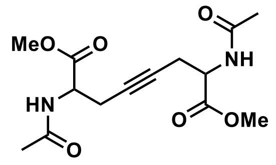

***O,O'*-(Dodec-6-yne-1,12-diyl) dimethyl dimalonate (S8)**. Prepared analogously as a white solid (45.3 mg, 91%). ^{1}H NMR (400 MHz, $CDCl_3$): δ = 4.11 (t, *J* = 6.7 Hz, 4H), 3.71 (s, 6H), 3.35 (s, 4H), 2.19 – 2.05 (m, 4H), 1.72 – 1.59 (m, 4H), 1.44 (ddt, *J* = 15.0, 8.0, 4.3 Hz, 8H). ^{13}C NMR (100 MHz, $CDCl_3$): δ = 167.1, 166.6, 80.1, 65.6, 52.5, 41.4, 28.7, 28.0, 25.1, 18.7. The analytical and spectroscopic data are in agreement with those reported in the literature.[4]

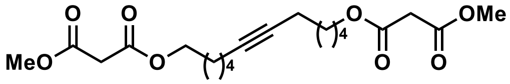

***N*^{1},*N*^{8}-Dimethoxy-*N*^{1},*N*^{8}-dimethyloct-4-ynediamide (S9)**. Prepared analogously as a white solid (16.7 mg, 52%). ^{1}H NMR (400 MHz, $CDCl_3$): δ = 3.69 (s, 6H), 3.18 (s, 6H), 2.63 (t, *J* = 7.3 Hz, 4H), 2.47 (dd, *J* = 8.7, 6.7 Hz, 4H). ^{13}C NMR (100 MHz, $CDCl_3$): δ = 172.9, 79.6, 61.4, 32.3, 31.7, 14.4. The analytical and spectroscopic data are in agreement with those reported in the literature.[4]

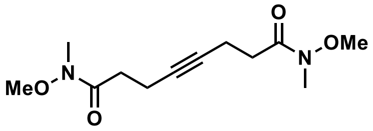

2,2'-(Octadec-9-yne-1,18-diyl)bis(isoindoline-1,3-dione) (S10). Prepared analogously as a white solid (60.0 mg, 89%). ^{1}H NMR (400 MHz, $CDCl_3$): δ = 7.81 (dt, *J* = 7.2, 3.6 Hz, 4H), 7.68 (dd, *J* = 5.4, 3.0 Hz, 4H), 3.65 (t, *J* = 7.3 Hz, 4H), 2.14 – 2.05 (m, 4H), 1.64 (t, *J* = 7.2 Hz, 4H), 1.43 (t, *J* = 7.3 Hz, 4H), 1.34 – 1.25 (m, 16H). ^{13}C NMR (100 MHz, $CDCl_3$): δ = 168.5, 133.9, 132.3, 123.2, 80.3, 38.1, 29.2, 29.1, 28.9,

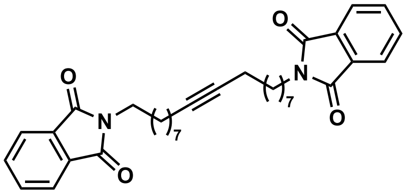

28.7, 26.9, 18.8. The analytical and spectroscopic data are in agreement with those reported in the literature.[4]

N

N^1,N^{12}-Dibutyldodec-6-yne-1,12-diamine (S11). *N*-Butyloct-6-yn-1-amine (45.3 mg, 25.0 µmol, 1.00 equiv)

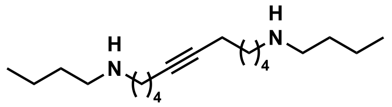

was added to a stirred suspension of complex **16a** (13.9 mg, 12.5 µmol, 5 mol%) and powdered molecular sieves 5Å (250 mg) in toluene (1.25 mL) at 60°C under argon atmosphere. After stirring for 12 h, the mixture was filtered through a short pad of Celite®, which was rinsed with MeOH (20 mL). The combined filtrates were evaporated and the residue was dissolved in $CHCl_3$ (1 mL). EtOAc (20 mL) was added to precipitate the product. The supernatant solution was decanted off and the remaining solid was dissolved in CH_2Cl_2 (10 mL). The organic layer was washed with sat. aq. $NaHCO_3$ (10 mL), dried over $MgSO_4$, filtered and concentrated *in vacuo* to give the title compound as a beige solid (38.6 mg, 71%). ^{1}H NMR (400 MHz, $CDCl_3$): δ = 2.59 (td, *J* = 7.3, 1.9 Hz, 8H), 2.18 – 2.09 (m, 4H), 1.55 – 1.29 (m, 20H), 0.90 (t, *J* = 7.3 Hz, 6H). ^{13}C NMR (100 MHz, $CDCl_3$): δ = 80.3, 50.1, 49.9, 32.4, 29.8, 29.2, 26.8, 20.7, 18.9, 14.2.. IR (film): ṽ 3260, 2930, 2851, 1460, 1414, 1052, 1023, 995, 725, 420 cm^{-1}. HRMS-ESI (m/z): calc'd. for $C_{18}H_{34}O_{32}^+$ [M]$^+$, 283.2632; found, 283.2633. The analytical and spectroscopic data are in agreement with those reported in the literature.[4]

1,12-Di(piperidin-1-yl)dodec-6-yne (S11). Prepared analogously as a white solid (36.7 mg, 88%). ^{1}H NMR (400 MHz, $CDCl_3$): δ =2.42 – 2.31 (m, 8H), 2.32 – 2.24 (m, 4H), 2.16 – 2.07 (m, 4H), 1.57 (p, *J* = 5.6 Hz, 8H), 1.49 – 1.33 (m, 16H). ^{13}C NMR (100 MHz, $CDCl_3$): δ = 80.3, 59.5, 54.7, 29.2, 27.1, 26.5, 26.0, 24.6, 18.8. The analytical and spectroscopic data are in agreement with those reported in the literature.[4]

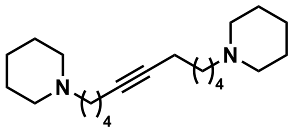

Hex-3-yne-1,6-diyl bis(4-methylbenzenesulfonate) (S12). Prepared analogously as a white solid (43.8 mg, 83%). ^{1}H NMR (400 MHz, $CDCl_3$): δ = 7.82 – 7.75 (m, 4H), 7.35 (d, *J* = 8.0 Hz, 4H), 4.04 – 3.97 (m, 4H), 2.45 (m, 10H). ^{13}C NMR (100 MHz, $CDCl_3$): δ = 145.1, 133.0, 130.0, 128.1, 76.9, 67.9, 21.8, 19.8. The analytical and spectroscopic data are in agreement with those reported in the literature.[3]

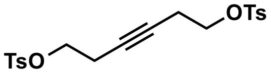

Representative Procedure for Ring Closing Alkyne Metathesis. 1,8-Dioxacyclotetradec-11-yne-2,7-dione (S13). Di(pent-3-yn-1-yl) adipate (27.8 mg, 10.0 µmol, 1.00 equiv) was added to a stirred suspension of complex **16a** (5.57 mg, 0.5 µmol, 5 mol%) and powdered molecular sieves 5Å (200 mg) in toluene (50 mL) at 23°C under argon atmosphere. The mixture was stirred for 24 h at this temperature before it was filtered through a short pad of Celite® which was carefully rinsed with ethyl acetate (20 mL). The combined filtrates were evaporated and the residue was purified by flash chromatography on silica gel (hexanes/*t*-butyl methyl ether 6:4 to 1:4) to give the title compound as a white solid (19.1 mg, 85%). ^{1}H NMR (400 MHz, $CDCl_3$): δ = 4.18 – 4.10 (m, 4H), 2.56 – 2.49 (m, 4H), 2.39 (ddt,

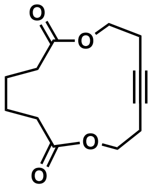

J = 6.0, 3.8, 1.8 Hz, 4H), 1.79 – 1.69 (m, 4H). ^{13}C NMR (100 MHz, $CDCl_3$): δ = 173.3, 78.1, 62.7, 35.1, 25.2, 19.3. The analytical and spectroscopic data are in agreement with those reported in the literature.[3]

1,6-Dioxacyclododec-9-yne-2,5-dione (S14). Prepared analogously, but the reaction was carried out at 60°C; white solid (18.6 mg, 95%). ^{1}H NMR (400 MHz, $CDCl_3$): δ = 4.34 – 4.16 (m, 4H), 2.69 (s, 4H), 2.58 – 2.34 (m, 4H). ^{13}C NMR (100 MHz, $CDCl_3$): δ = 171.8, 78.9, 61.5, 30.1, 19.8. The analytical and spectroscopic data are in agreement with those reported in the literature.[3]

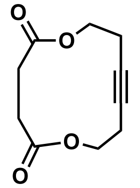

Compound S15. Prepared analogously as a white solid (32.1 mg, 98%). ^{1}H NMR (400 MHz, $CDCl_3$): δ =7.77 – 7.69 (m, 2H), 7.58 – 7.49 (m, 2H), 4.35 (t, J = 6.1 Hz, 4H), 2.24 – 2.15 (m, 4H), 1.79 (dq, J = 9.0, 5.8 Hz, 4H), 1.64 – 1.45 (m, 8H). ^{13}C NMR (100 MHz, $CDCl_3$): δ = 167.9, 132.4, 131.1, 129.0, 80.8, 66.5, 28.5, 28.2, 26.0, 18.8. The analytical and spectroscopic data are in agreement with those reported in the literature.[3]

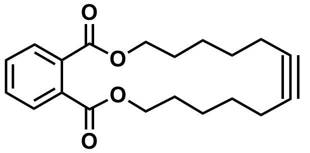

Compound S16. Prepared analogously as a white solid (51.4 mg, 98%). ^{1}H NMR (400 MHz, $CDCl_3$): δ = 8.61 (d, J = 2.3 Hz, 1H), 8.38 (dd, J = 8.5, 2.3 Hz, 1H), 7.85 (d, J = 8.5 Hz, 1H), 4.33 (td, J = 7.0, 4.7 Hz, 4H), 2.17 (h, J = 2.1 Hz, 4H), 1.75 (h, J = 7.2 Hz, 4H), 1.48 – 1.28 (m, 28H). ^{13}C NMR (100 MHz, $CDCl_3$): δ = 166.5, 165.4, 148.9, 138.4, 133.4, 130.3, 126.0, 124.6, 80.7, 66.8, 29.8, 29.8, 29.7, 29.6, 29.5, 29.3, 29.3, 26.0, 25.9, 18.7. The analytical and spectroscopic data are in agreement with those reported in the literature.[3]

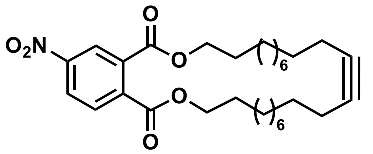

Compound S17. Prepared analogously as a white solid (26.8 mg, 99%). ^{1}H NMR (rotamers, 400 MHz, $CDCl_3$): δ = 7.76 (dd, J = 7.7, 2.2 Hz, 2H), 7.63 – 7.53 (m, 2H), 7.45 – 7.27 (m, 4H), 4.73 – 4.49 (m, 2H), 4.22 (q, J = 5.2 Hz, 2H), 3.92 – 3.66 (m, 1H), 3.42 (s, 0H), 2.99 (s, 1H), 2.70 – 2.35 (m, 1H), 2.32 – 2.13 (m, 3H), 2.13 – 2.01 (m, 1H), 1.86 – 1.69 (m, 2H), 1.61 – 0.77 (m, 14H), 0.70 (s, 2H). ^{13}C NMR (rotamers, 100 MHz, $CDCl_3$): δ = 173.5, 173.4, 144.3, 144.2, 144.1, 141.6, 141.6, 127.8, 127.8, 127.7, 127.3, 127.2, 127.2, 127.1, 124.8, 124.7, 120.1, 120.1, 120.0, 120.0, 79.8, 79.6, 77.4, 66.6, 66.4, 63.6, 47.8, 47.5, 35.1, 34.8, 34.7, 32.6, 32.5, 28.6, 28.4, 26.9, 26.8, 23.7, 23.6, 19.7, 18.4, 17.9, 14.2, 14.0. The analytical and spectroscopic data are in agreement with those reported in the literature.[3, 7]

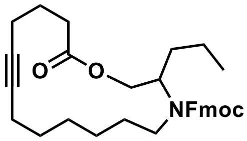

Compound S18. Prepared analogously at 23°C; white solid (4.42 mg, 85%). ^{1}H NMR (400 MHz, $CDCl_3$): δ 5.19 (p, J = 1.1 Hz, 1H), 5.14 (d, J = 1.2 Hz, 1H), 5.12 – 5.06 (m, 1H), 5.05 (t, J = 1.5 Hz, 1H), 5.01 (p, J = 1.2 Hz, 1H), 4.12 (d, J = 4.8 Hz, 1H), 3.71 (dd, J = 10.4, 5.0 Hz, 1H), 3.60 (dd, J = 10.4, 5.7 Hz, 1H), 3.31 (dd, J = 2.1, 0.8 Hz, 1H), 3.19 (d, J = 17.6 Hz, 1H), 3.14 (dd, J = 4.8, 2.1 Hz, 1H), 2.93 (d, J = 17.6 Hz, 1H), 2.55 – 2.32 (m, 6H), 0.91 (s, 9H), 0.89 (s, 9H), 0.14 (s, 3H), 0.05 (d, J = 0.8 Hz, 9H). ^{13}C NMR (100 MHz, $CDCl_3$): δ = 171.7, 143.9, 141.2, 114.2,

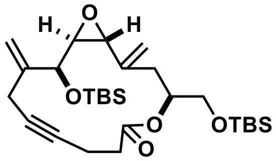

114.1, 80.4, 78.8, 74.9, 72.6, 64.5, 62.7, 57.0, 34.3, 34.1, 26.0, 25.9, 22.7, 18.4, 18.4, 15.3, –4.7, –4.9, –5.3, –5.3. The analytical and spectroscopic data are in agreement with those reported in the literature.[9]

Compound S19. Prepared analogously at 60°C; white solid (5.30 mg, 75%). ^{1}H NMR (400 MHz, $CDCl_3$): δ = 6.96 (s, 1H), 6.55 (s, 1H), 5.34 (dd, *J* = 7.3, 3.4 Hz, 1H), 4.70 (dd, *J* = 6.4, 4.8 Hz, 1H), 3.94 (dd, *J* = 6.6, 1.9 Hz, 1H),

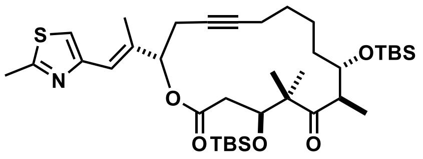

3.31 – 3.12 (m, 1H), 2.83 – 2.74 (m, 1H), 2.71 (s, 3H), 2.71 – 2.53 (m, 3H), 2.18 (d, *J* = 1.3 Hz, 3H), 2.39 – 1.94 (m, 3H), 1.81 – 1.35 (m, 1H), 1.17 (s, 3H), 1.15 (s, 3H), 1.11 (s, 3H), 0.95 – 0.94 (m, 3H), 0.92 (s, 9H), 0.87 (s, 9H), 0.10 (s, 6H), 0.09 (s, 3H), 0.07 (d, *J* = 2.2 Hz, 6H). ^{13}C NMR (100 MHz, $CDCl_3$): δ = 216.7, 170.2, 164.9, 152.6, 137.0, 120.7, 116.9, 82.3, 78.2, 77.4, 76.5, 72.8, 54.7, 53.5, 44.6, 41.8, 39.1, 29.9, 26.4, 26.2, 26.1, 24.3, 21.2, 20.7, 19.4, 18.8, 18.7, 18.5, 17.1, 15.2, −3.1, −3.6, −3.9, −3.9. The analytical and spectroscopic data are in agreement with those reported in the literature.[8]

Compound S20. (3*S*,5*R*,6*S*)-6-hydroxy-1-methoxy-3-methyl-1-oxo-5-((2,2,6,6-tetramethylpiperidin-1-yl)oxy)non-7-yn-3-yl (*S*,*Z*)-3-acetoxy-5-(prop-1-en-2-yl)non-2-en-7-ynoate (50.0 mg, 83.1 µmol, 1.00 equiv)

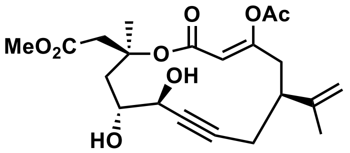

was added to a stirred suspension of complex **16a** (27.8 mg, 24.9 µmol, 30 mol%) and powdered molecular sieves 5Å (20 mg) in toluene (42 mL) at 110°C under Ar. The mixture was stirred for 45 min at this temperature before it was filtered through a short pad of Celite®, which was carefully rinsed with ethyl acetate (100 mL). The combined filtrates were evaporated and the residue was purified by flash chromatography on silica gel (hexanes/EA 3:1). Zinc dust (217 mg, 332 µmol) was added to a solution of this compound in HOAc/THF/H_2O (0.3 mL, 3:1:1). The suspension was vigorously stirred for 3 h at room temperature before all insoluble materials were filtered off through a pad of Celite. The filtrate was diluted with sat. aq. $NaHCO_3$ (10 mL), the aqueous phase was extracted with EtOAc (3 x 20 mL), the combined organic layers were washed with brine, dried over $MgSO_4$, filtered and concentrated. The residue was purified by flash chromatography on silica (hexanes/EtOAc, 2:1 to 1:1) to afford the title compound as a colorless oil (23.0 mg, 68% over two steps). ^{1}H NMR (400 MHz, $CDCl_3$): δ = 5.61 (s, 1H), 4.82 (p, *J* = 1.6 Hz, 1H), 4.78 – 4.73 (m, 1H), 4.58 (q, *J* = 2.3 Hz, 1H), 4.18 – 4.11 (m, 1H), 3.84 – 3.77 (m, 1H), 3.75 (d, *J* = 1.7 Hz, 1H), 3.72 (s, 3H), 3.42 (dd, *J* = 14.4, 5.0 Hz, 1H), 3.06 (d, *J* = 14.6 Hz, 1H), 2.74 – 2.69 (m, 1H), 2.67 (d, *J* = 1.9 Hz, 1H), 2.63 (d, *J* = 1.4 Hz, 1H), 2.48 (tt, *J* = 9.0, 4.6 Hz, 1H), 2.39 (dd, *J* = 4.1, 1.7 Hz, 1H), 2.34 (dd, *J* = 4.1, 1.7 Hz, 1H), 2.30 (dd, *J* = 8.8, 3.0 Hz, 1H), 2.11 (s, 3H), 2.05 (dd, *J* = 16.2, 8.4 Hz, 1H), 1.71 (s, 3H), 1.69 (t, *J* = 1.0 Hz, 3H). ^{13}C NMR (100 MHz, $CDCl_3$): δ = 172.9, 168.1, 165.1, 163.2, 144.9, 114.2, 112.9, 85.7, 83.2, 81.4, 71.6, 67.3, 52.4, 44.7, 42.6, 40.1, 33.6, 25.8, 24.0, 21.2, 20.1. The analytical and spectroscopic data are in agreement with those reported in the literature.[10]

KINETIC STUDIES

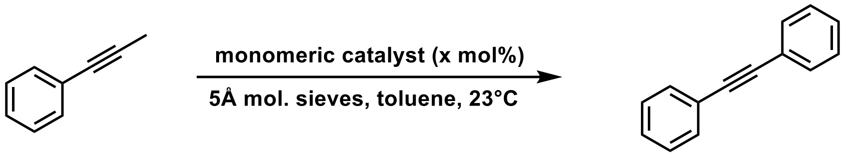

Conversion-time Diagrams. A 10 mL Schlenk flask was equipped with a magnetic stir bar and was flame dried under vacuum. The flask was filled with argon, charged with 1-phenyl-1-propyne (11.6 mg, 100 µmol, 1.00 equiv), powdered molecular sieves 5Å (100 mg) and toluene (500 µL). Biphenyl (15.4 mg, 100 µmol, 1.00 equiv) was added from a stock solution as an internal standard. The mixture was vigorously stirred (1000 rpm) at ambient temperature and the monomeric catalyst 16a or 16b (x mol%) was added. At the specified reaction times, an aliquot was taken, diluted with *t*-butyl methyl ether (1 mL), filtered and analyzed *via* GC-MS analysis.

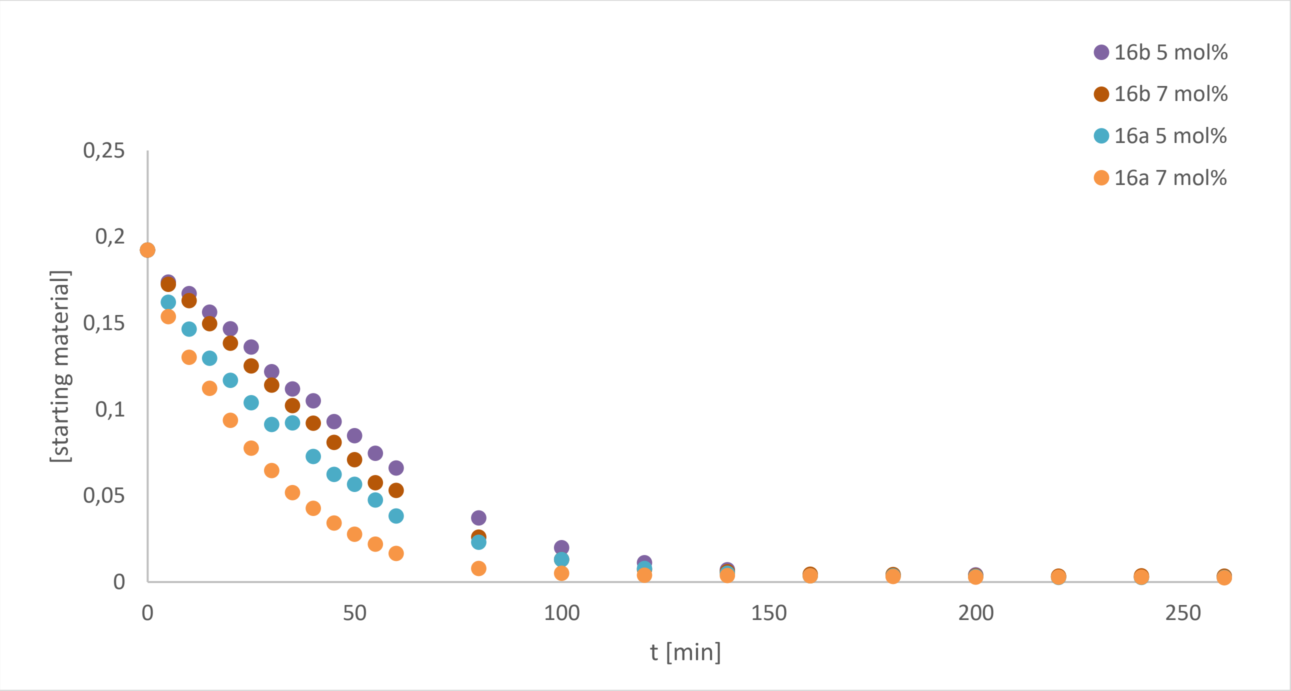

Figure S-9. Conversion-time diagram: comparison of the monomeric catalysts at different loadings.

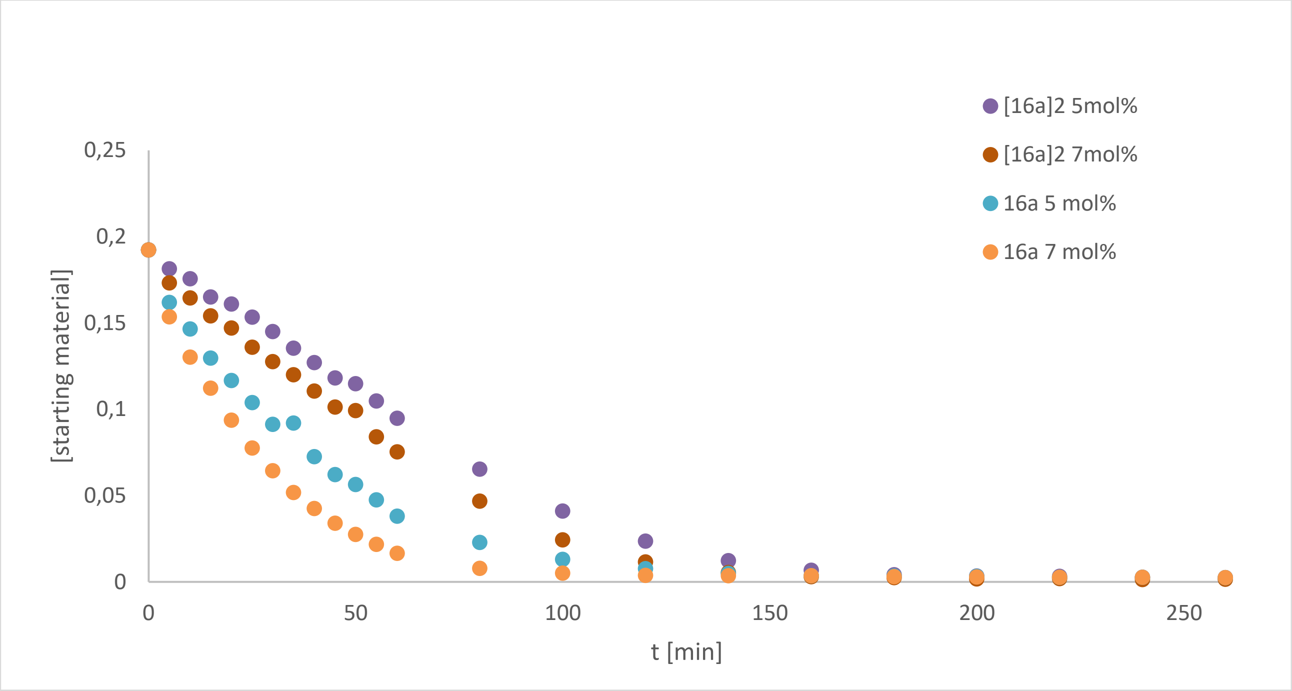

Figure S-10. Conversion-time diagram: comparison of the monomeric and dimeric complexes [**16a**] and [**16a**]$_2$ at different loadings.

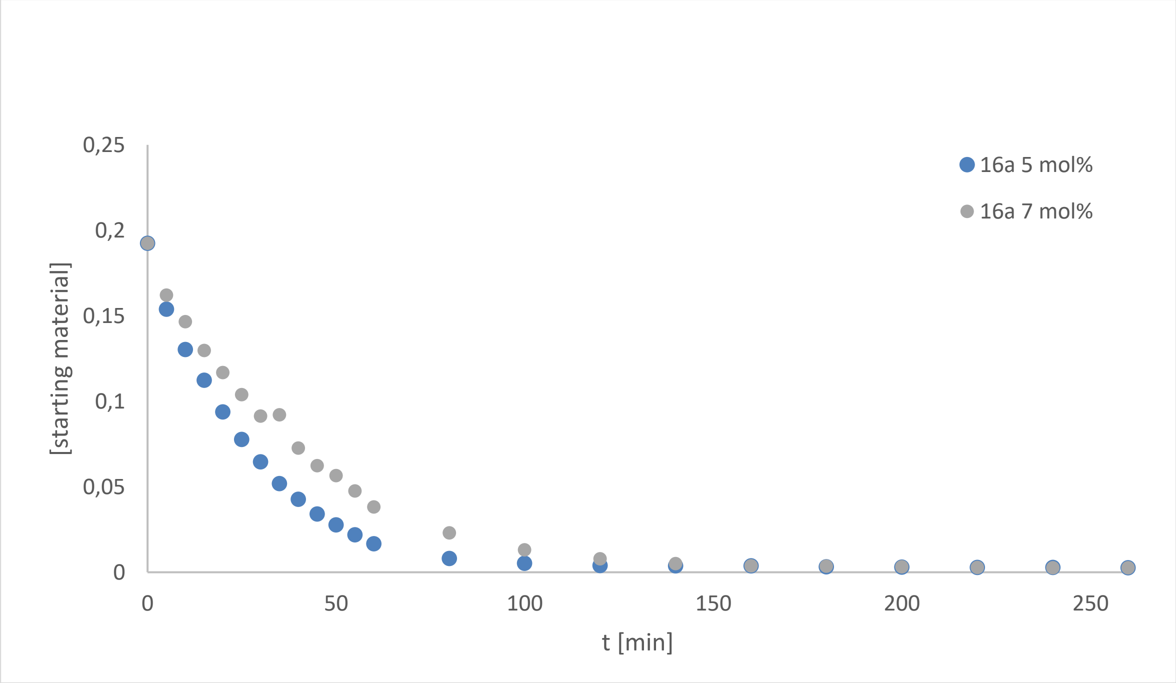

Figure S-11. Conversion-time diagram for monomeric **16a** at different catalyst loadings.

Normalized time scale analysis. The reaction order for both monomeric catalysts was determined by normalized time scale analysis.[11]

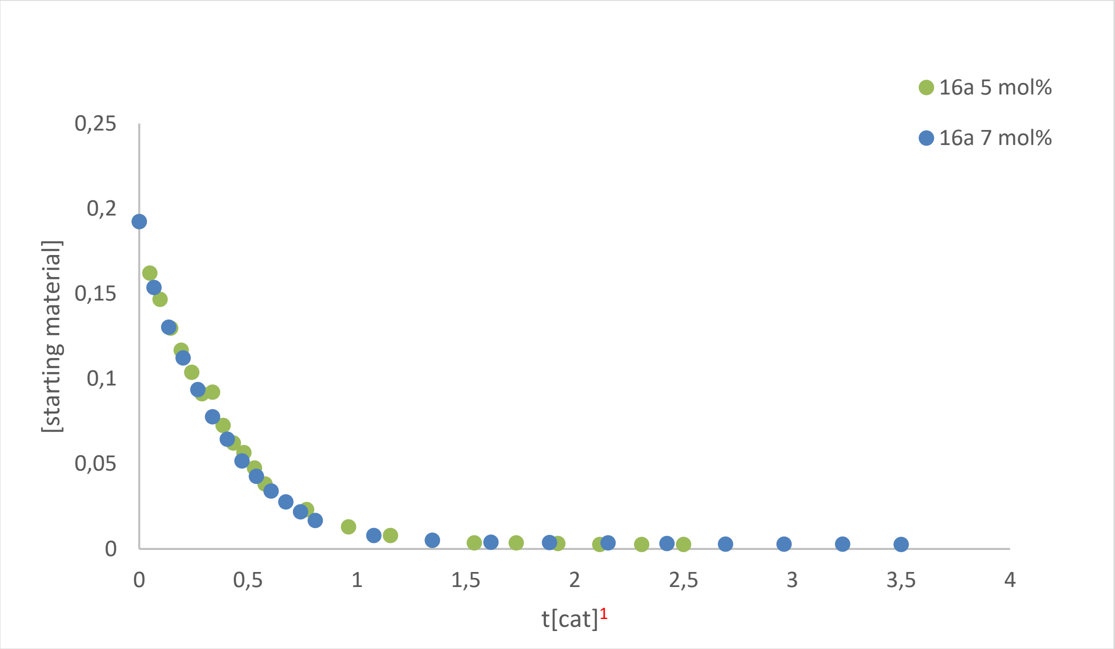

Figure S-12. Normalized time scale analysis assuming the reaction be first order in catalyst **16a**

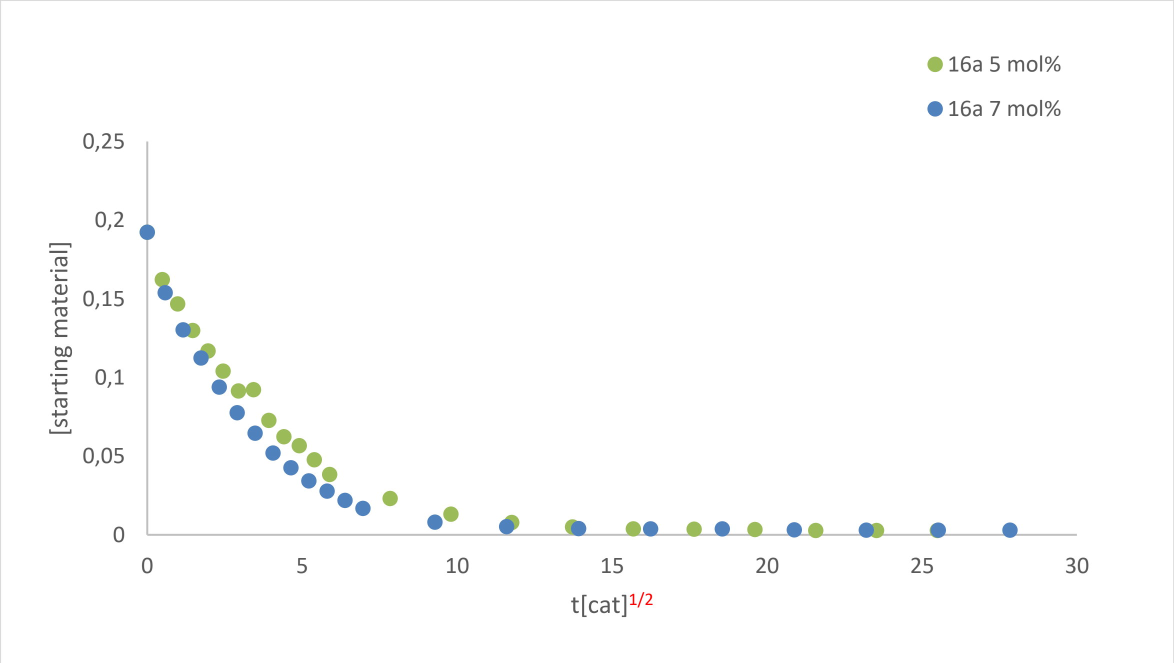

Figure S-13. Normalized time scale analysis assuming the reaction be order 1/2 in catalyst **16a**

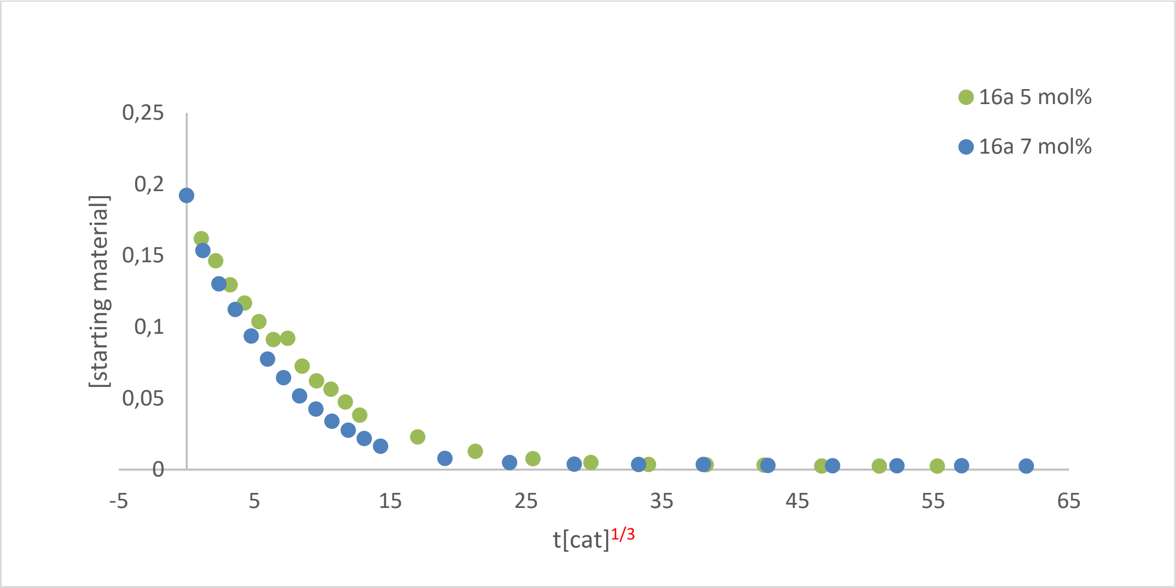

Figure S-14. Normalized time scale analysis assuming the reaction be order 1/3 in catalyst **16a**.

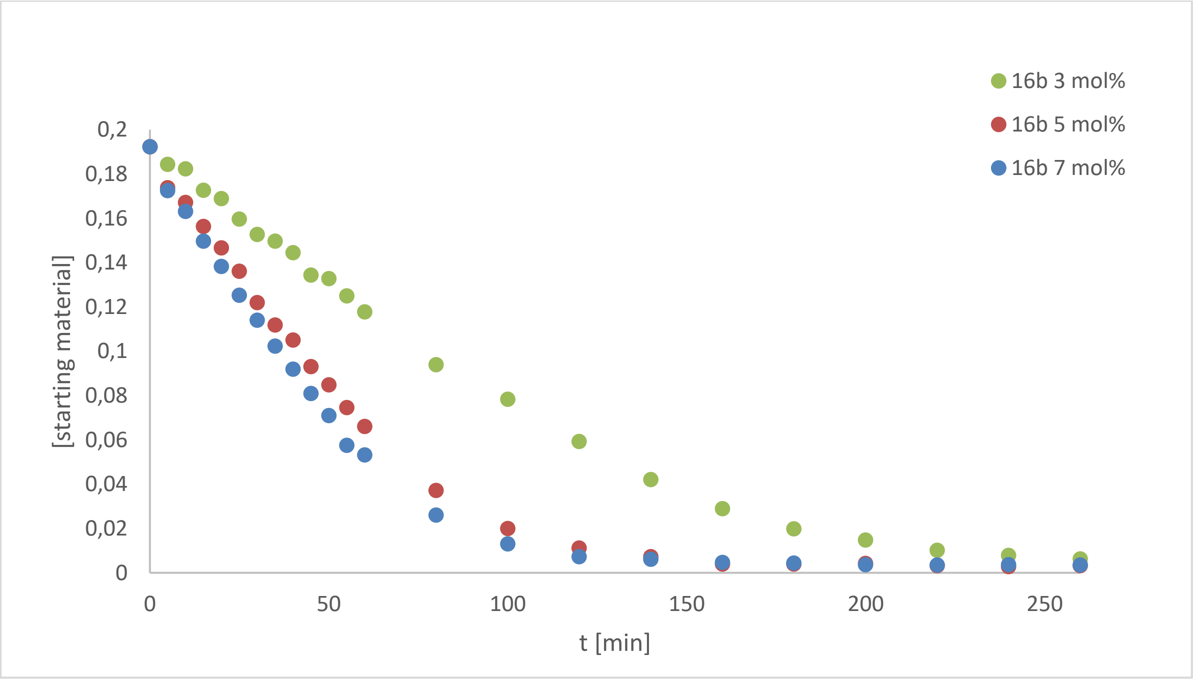

Figure S-15. Conversion-time diagram for monomeric catalyst **16b** at different catalyst loadings.

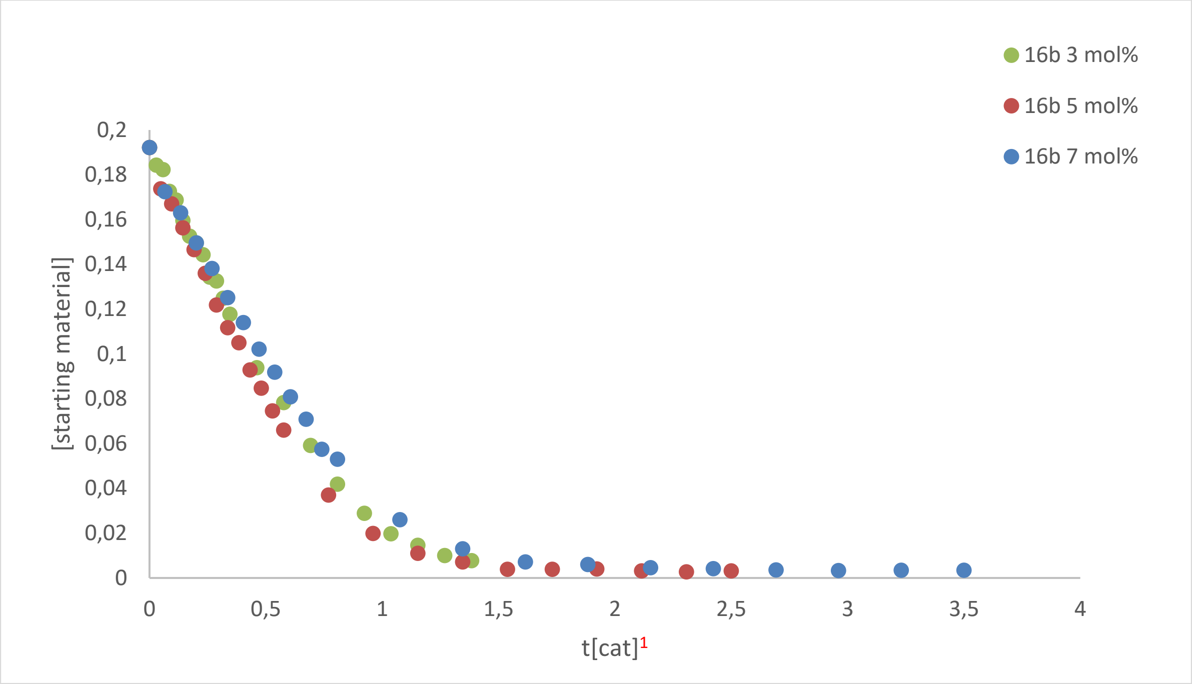

Figure S-16. Normalized time scale analysis assuming the reaction be first order in catalyst **16b**.

REFERENCES

1. Feng, X.; Wu, J.; Enkelmann, V.; Müllen, K., Hexa-peri-hexabenzocoronenes by Efficient Oxidative Cyclodehydrogenation: The Role of the Oligophenylene Precursors. *Organic Letters* **2006,** *8* (6), 1145-1148.
2. McCullough, L. G.; Schrock, R. R.; Dewan, J. C.; Murdzek, J. C., Multiple metal-carbon bonds. 38. Preparation of trialkoxymolybdenum(VI) alkylidyne complexes, their reactions with acetylenes, and the x-ray structure of Mo[C3(CMe3)2][OCH(CF3)2](C5H5N)2. *Journal of the American Chemical Society* **1985,** *107* (21), 5987-5998.
3. Heppekausen, J.; Stade, R.; Goddard, R.; Fürstner, A., Practical New Silyloxy-Based Alkyne Metathesis Catalysts with Optimized Activity and Selectivity Profiles. *Journal of the American Chemical Society* **2010,** *132* (32), 11045-11057.
4. Schaubach, S.; Gebauer, K.; Ungeheuer, F.; Hoffmeister, L.; Ilg, M. K.; Wirtz, C.; Fürstner, A., A Two-Component Alkyne Metathesis Catalyst System with an Improved Substrate Scope and Functional Group Tolerance: Development and Applications to Natural Product Synthesis. **2016,** *22* (25), 8494-8507.
5. Jin, G.; Zhang, X.; Cao, S., Transition-Metal-Free Sonogashira-Type Cross-Coupling of Alkynes with Fluoroarenes. *Organic Letters* **2013,** *15* (12), 3114-3117.
6. Qiu, S.; Zhang, C.; Qiu, R.; Yin, G.; Huang, J., One-Pot Domino Synthesis of Diarylalkynes/1,4-Diaryl-1,3-diynes by [9,9-Dimethyl-4,5-bis(diphenylphosphino)xanthene] (Xantphos)–Copper(I) Iodide–Palladium(II) Acetate-Catalyzed Double Sonogashira-Type Reaction. *Advanced Synthesis & Catalysis* **2018,** *360* (2), 313-321.
7. Fürstner, A.; Guth, O.; Rumbo, A.; Seidel, G., Ring Closing Alkyne Metathesis. Comparative Investigation of Two Different Catalyst Systems and Application to the Stereoselective Synthesis of Olfactory Lactones, Azamacrolides, and the Macrocyclic Perimeter of the Marine Alkaloid Nakadomarin A. *Journal of the American Chemical Society* **1999,** *121* (48), 11108-11113.
8. Fürstner, A.; Mathes, C.; Lehmann, C. W., Alkyne Metathesis: Development of a Novel Molybdenum-Based Catalyst System and Its Application to the Total Synthesis of Epothilone A and C. **2001,** *7* (24), 5299-5317.
9. Fürstner, A.; Flügge, S.; Larionov, O.; Takahashi, Y.; Kubota, T.; Kobayashi, J. i., Total Synthesis and Biological Evaluation of Amphidinolide V and Analogues. **2009,** *15* (16), 4011-4029.
10. Meng, Z.; Fürstner, A., Total Synthesis of (−)-Sinulariadiolide. A Transannular Approach. *Journal of the American Chemical Society* **2019,** *141* (2), 805-809.
11. Burés, J., A Simple Graphical Method to Determine the Order in Catalyst. *Angew. Chem. Int. Ed.* **2016,** *55* (6), 2028-2031.

^{1}H NMR of 1,3,4-tris-2’-bromophenylbenzene (11)[1], CDCl$_3$, 23 ºC

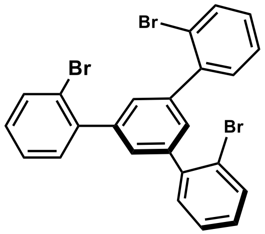

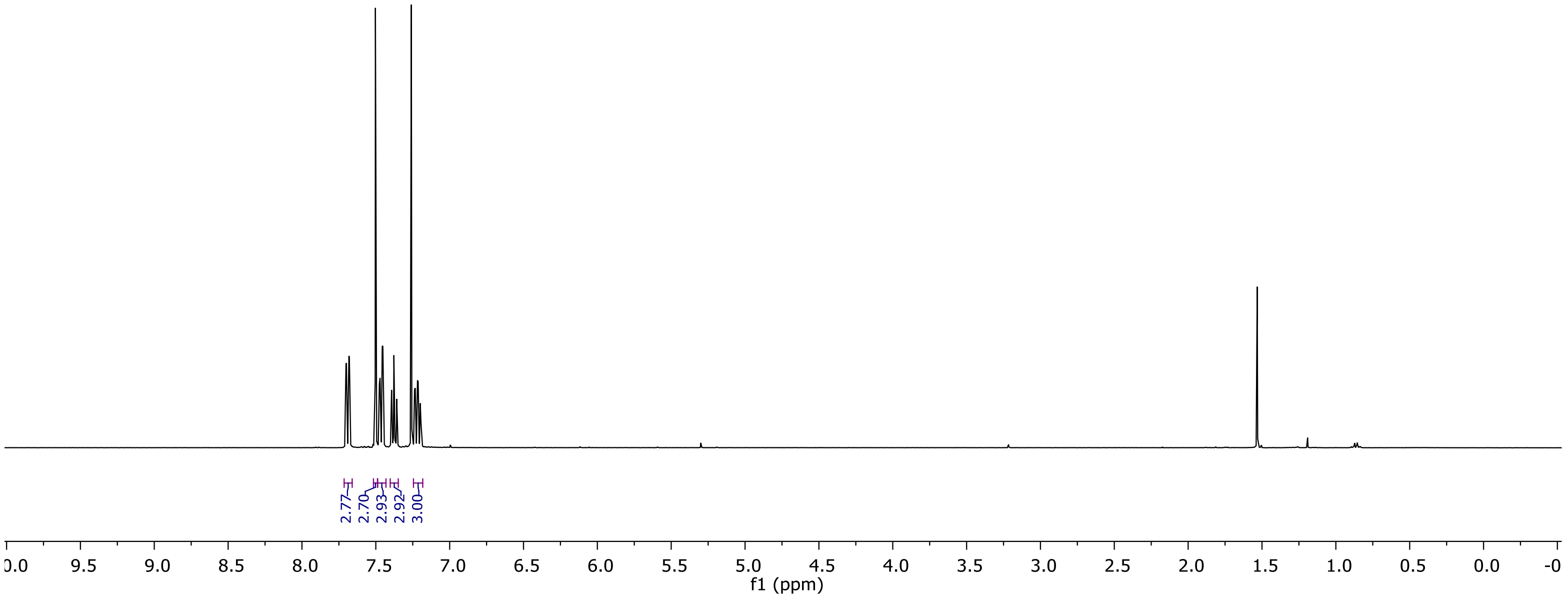

^{13}C NMR of 1,3,4-tris-2'-bromophenylbenzene (11)[1], $CDCl_3$, 23 ºC

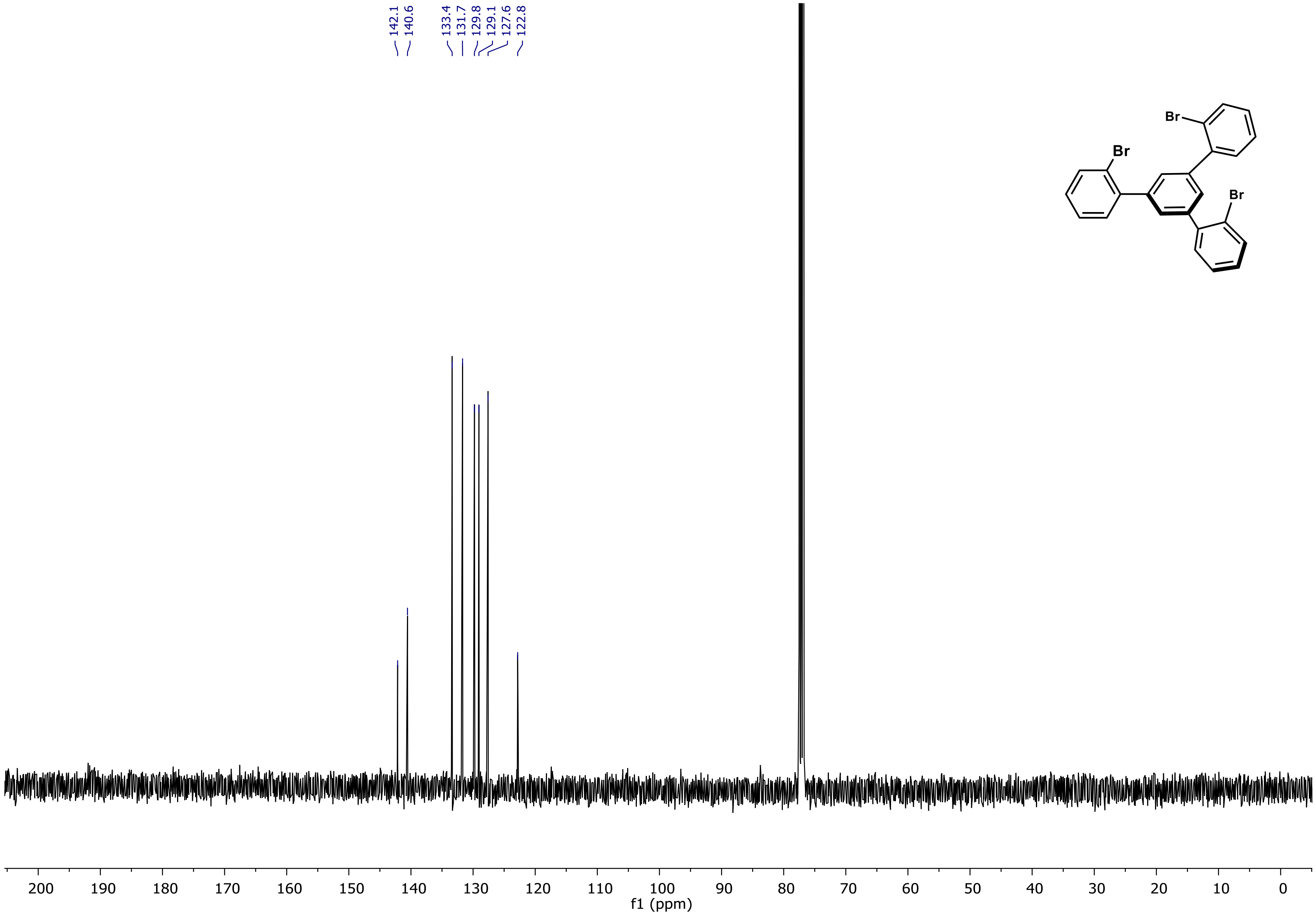

^{1}H NMR of Compound 12a, $CDCl_3$, 23 ºC

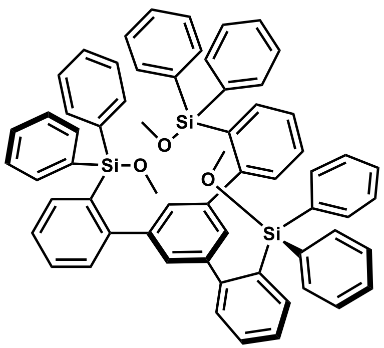

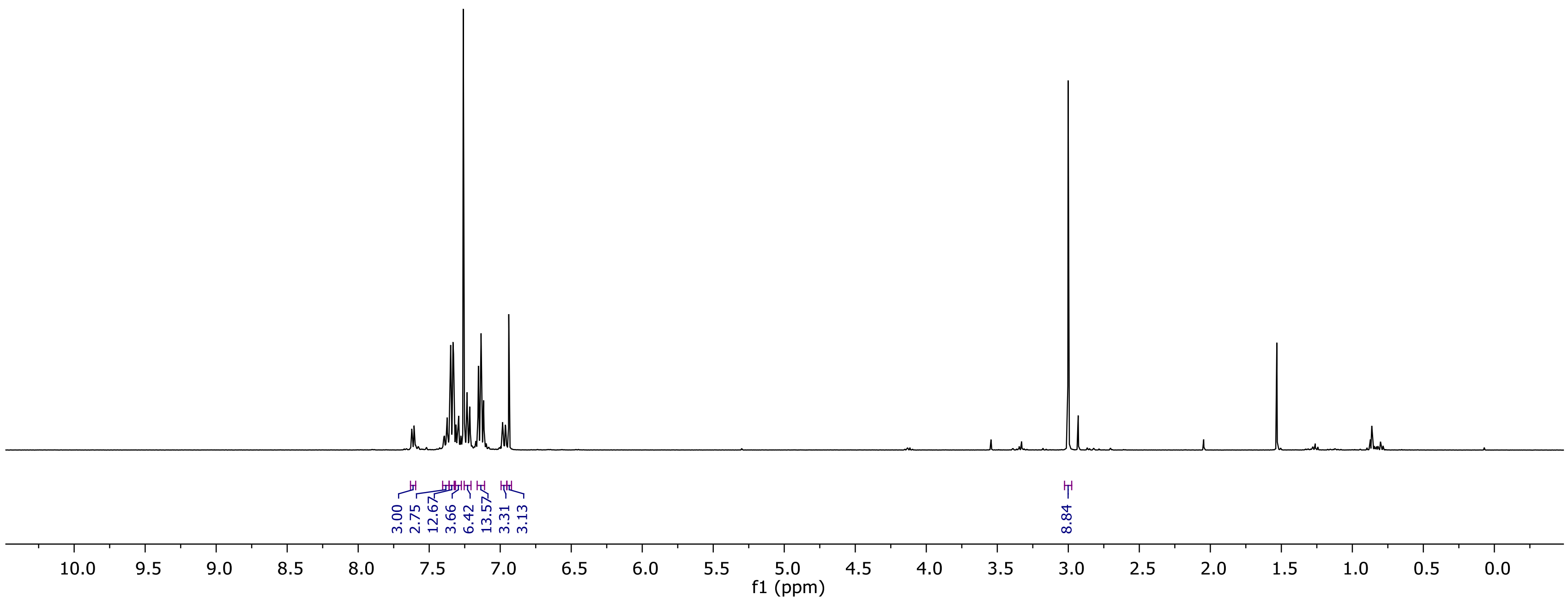

^{13}C NMR of Compound 12a, $CDCl_3$, 23 °C

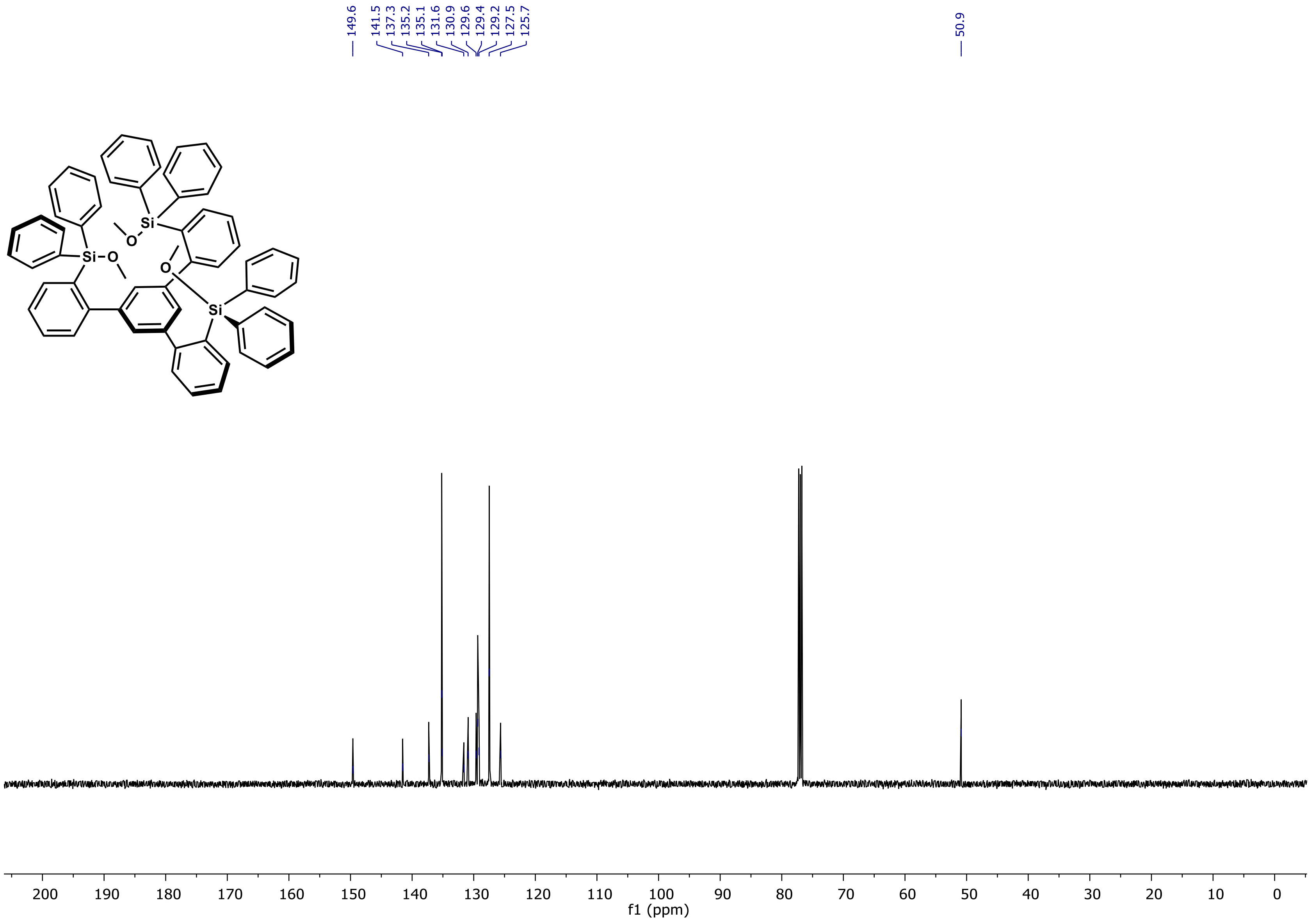

^{29}Si NMR of Compound 12a, $CDCl_3$, 23 ºC

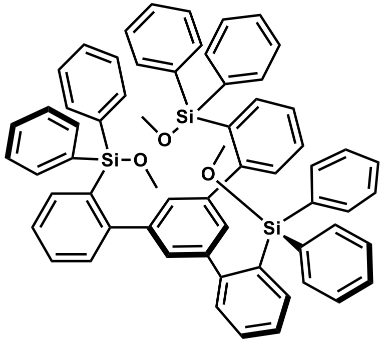

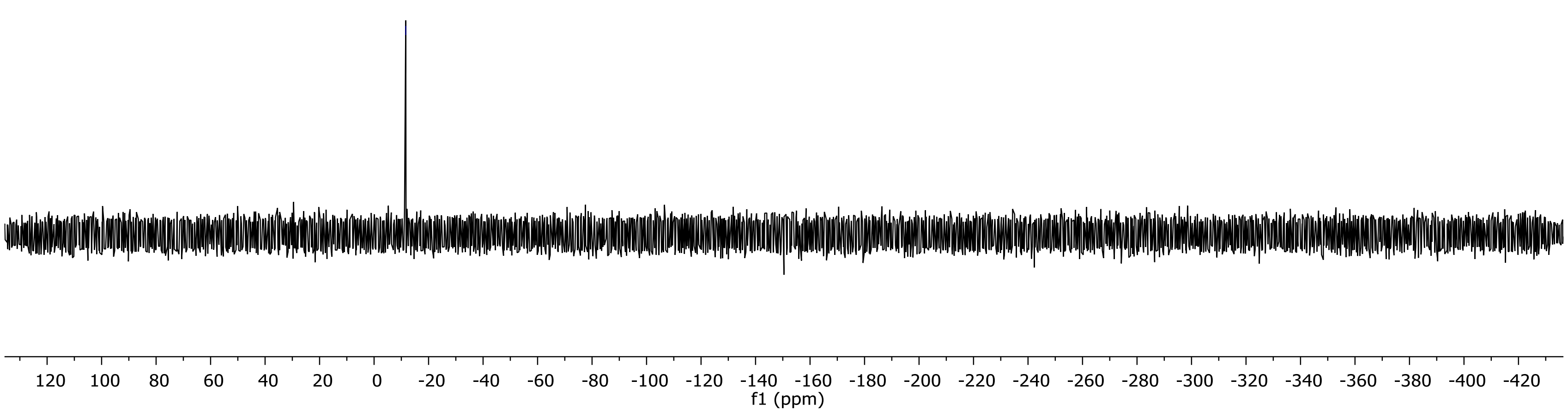

^{1}H NMR of Ligand 13a, $CDCl_3$, 23 ºC

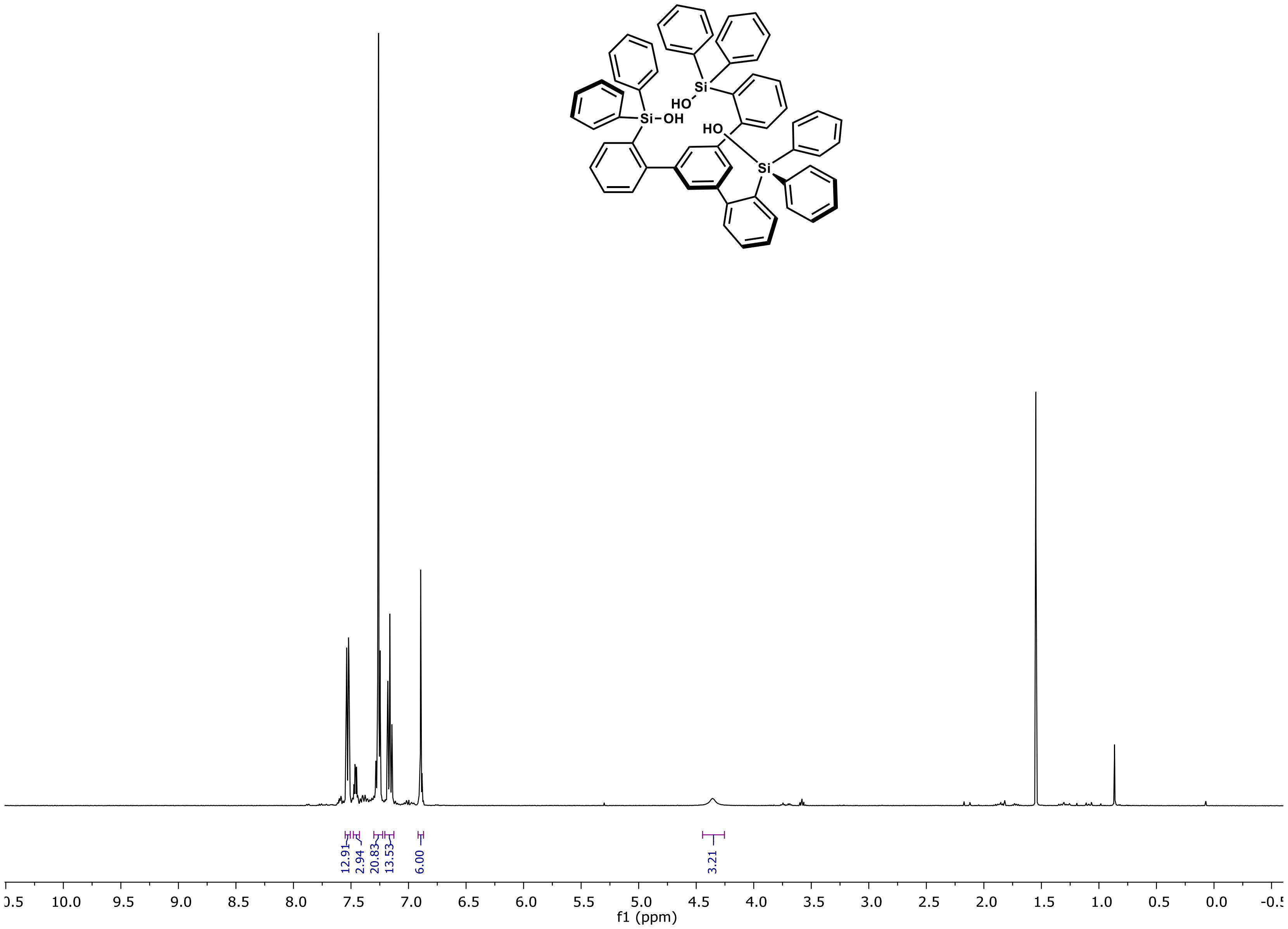

^{13}C NMR of Ligand 13a, $CDCl_3$, 23 °C

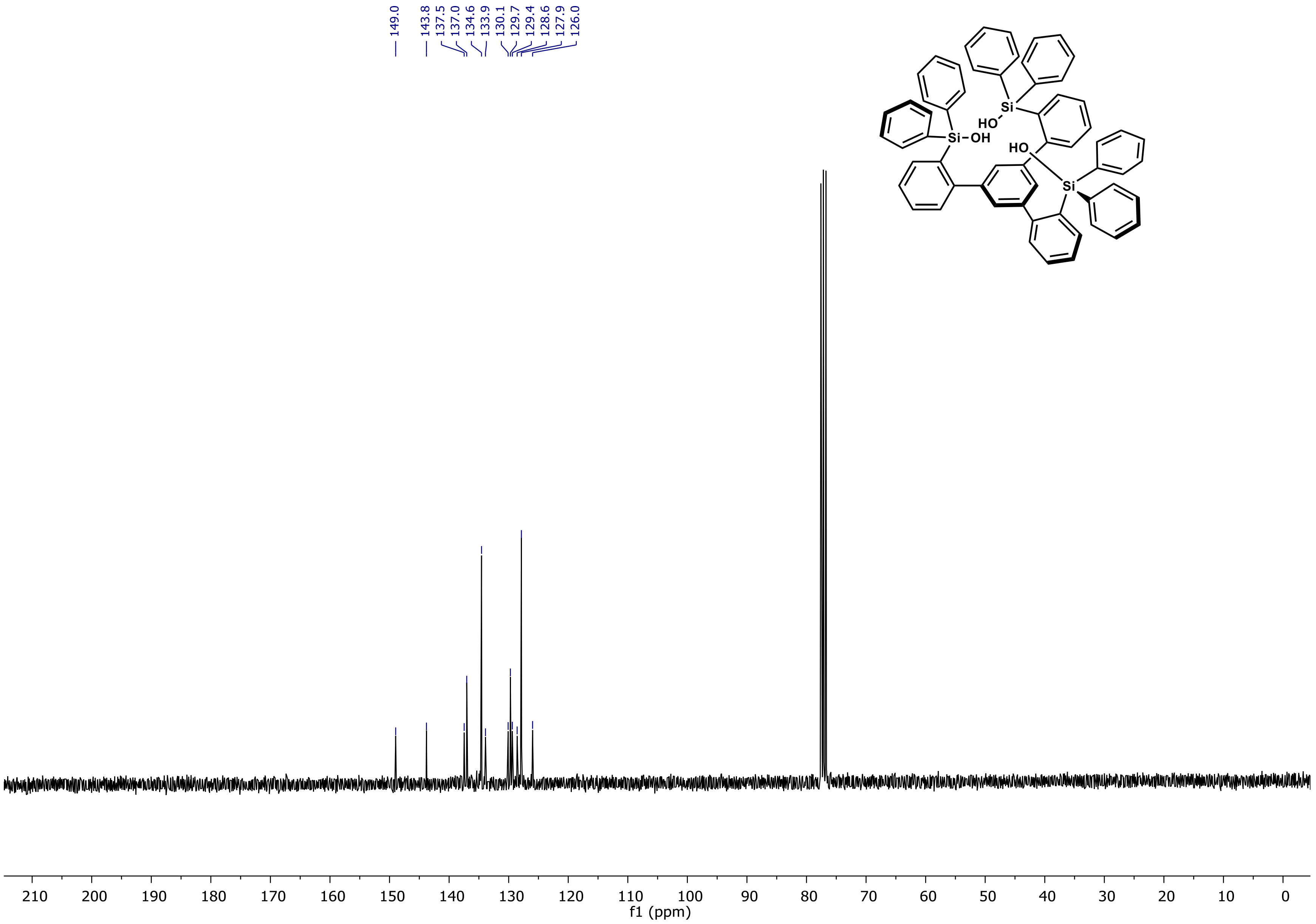

^{1}H NMR of Dimethoxybis(4-methoxyphenyl)silane (S1), C_6D_6, 23 ºC

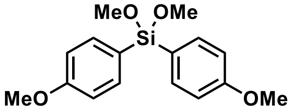

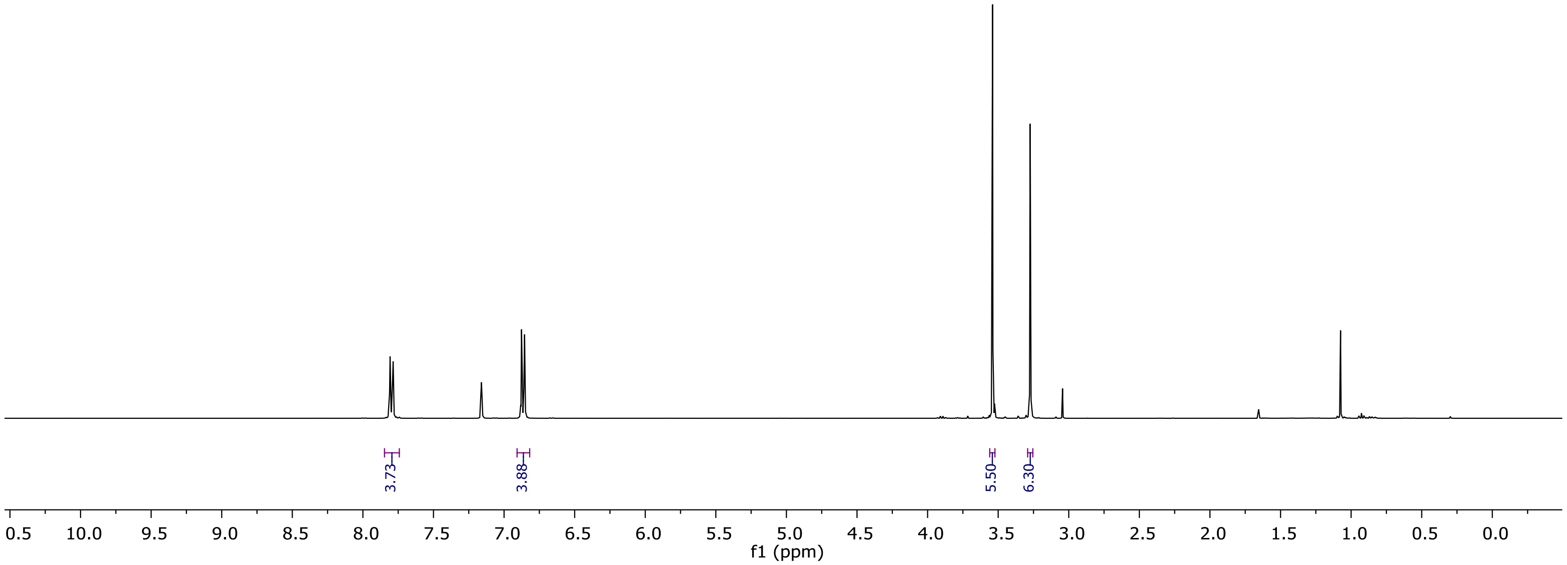

^{13}C NMR of Dimethoxybis(4-methoxyphenyl)silane (S1), C_6D_6, 23 ºC

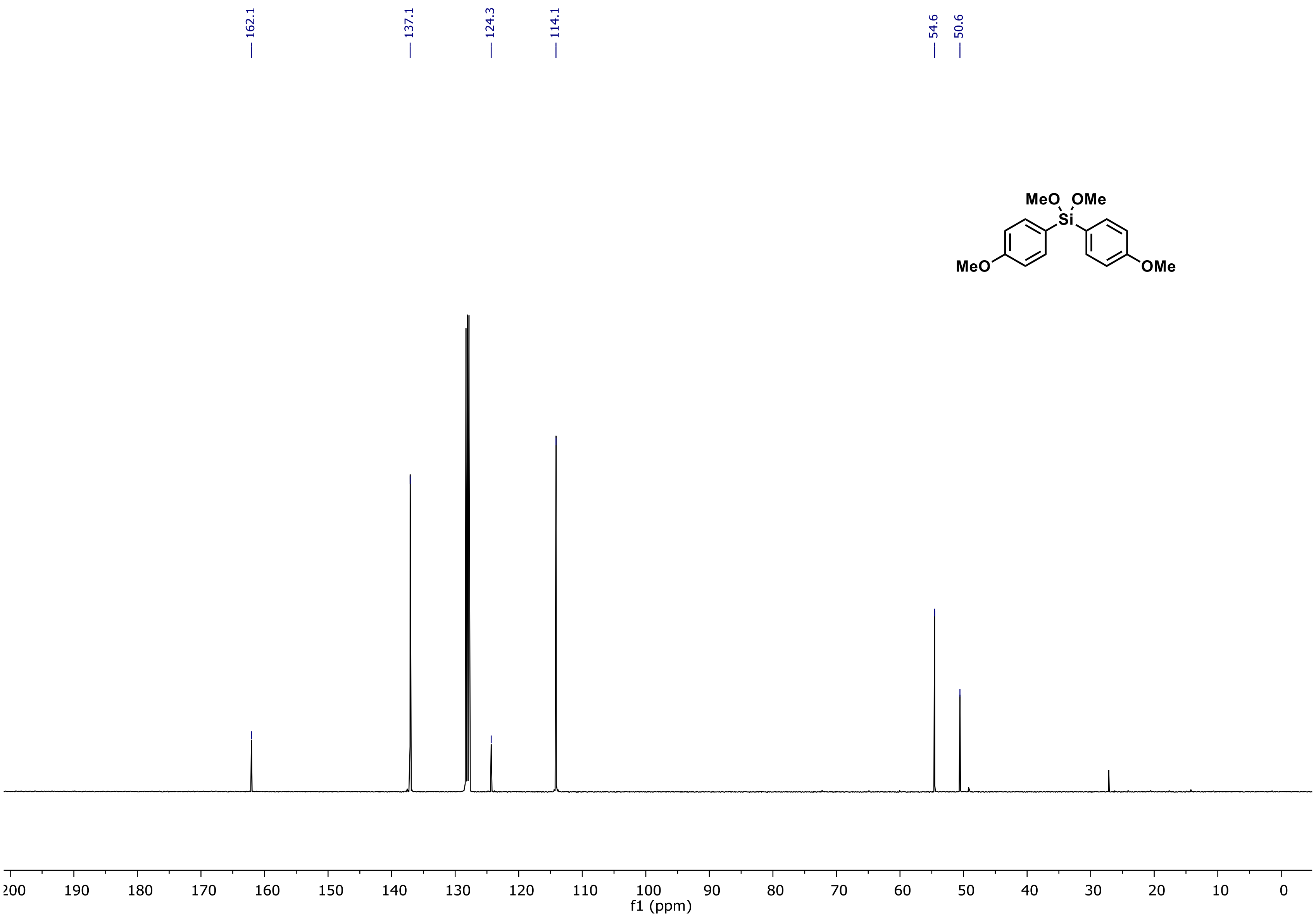

^{1}H NMR of Compound 12b, $CDCl_3$, 23 ºC

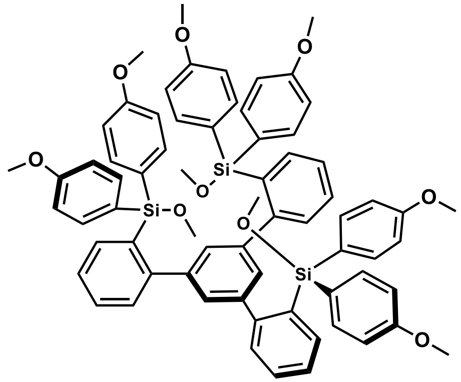

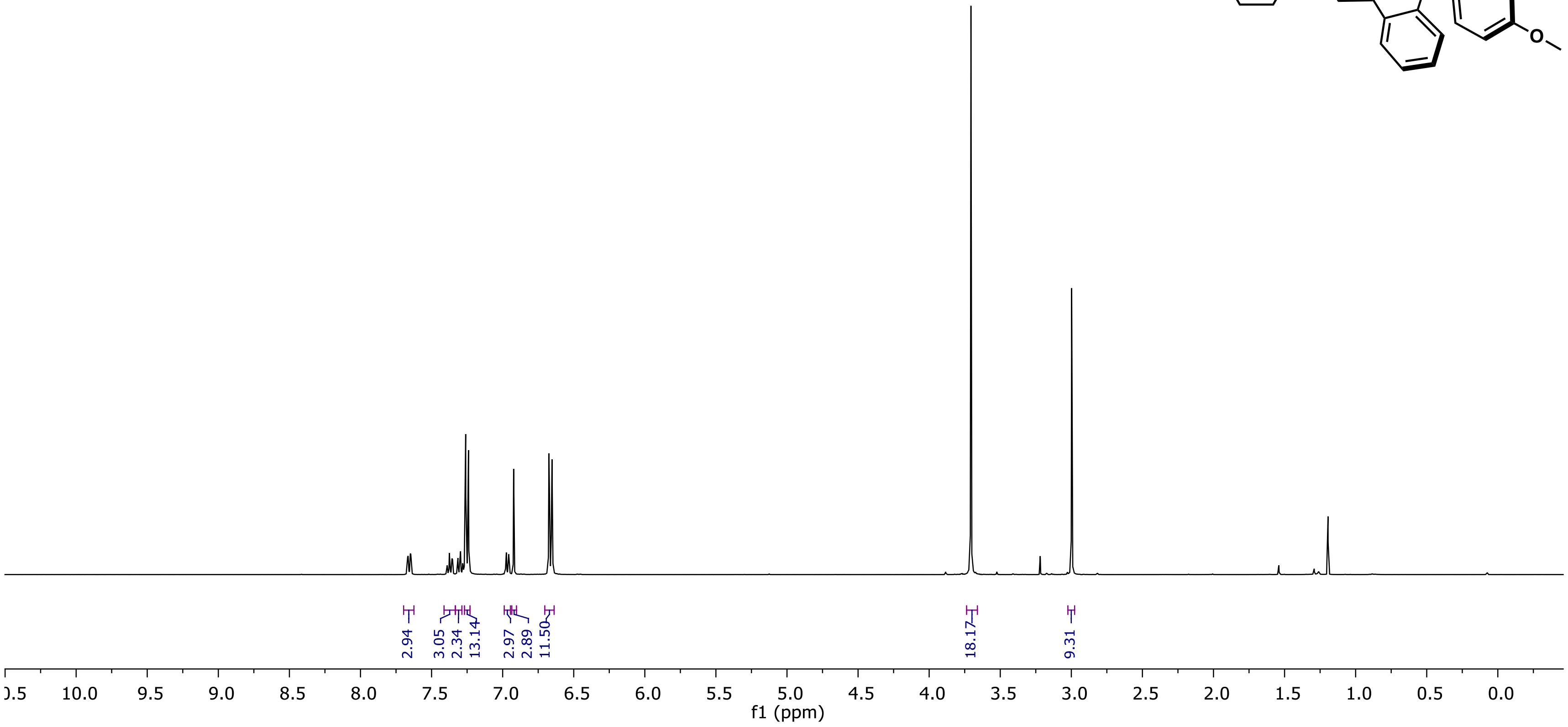

^{13}C NMR of of Compound 12b, $CDCl_3$, 23 ºC

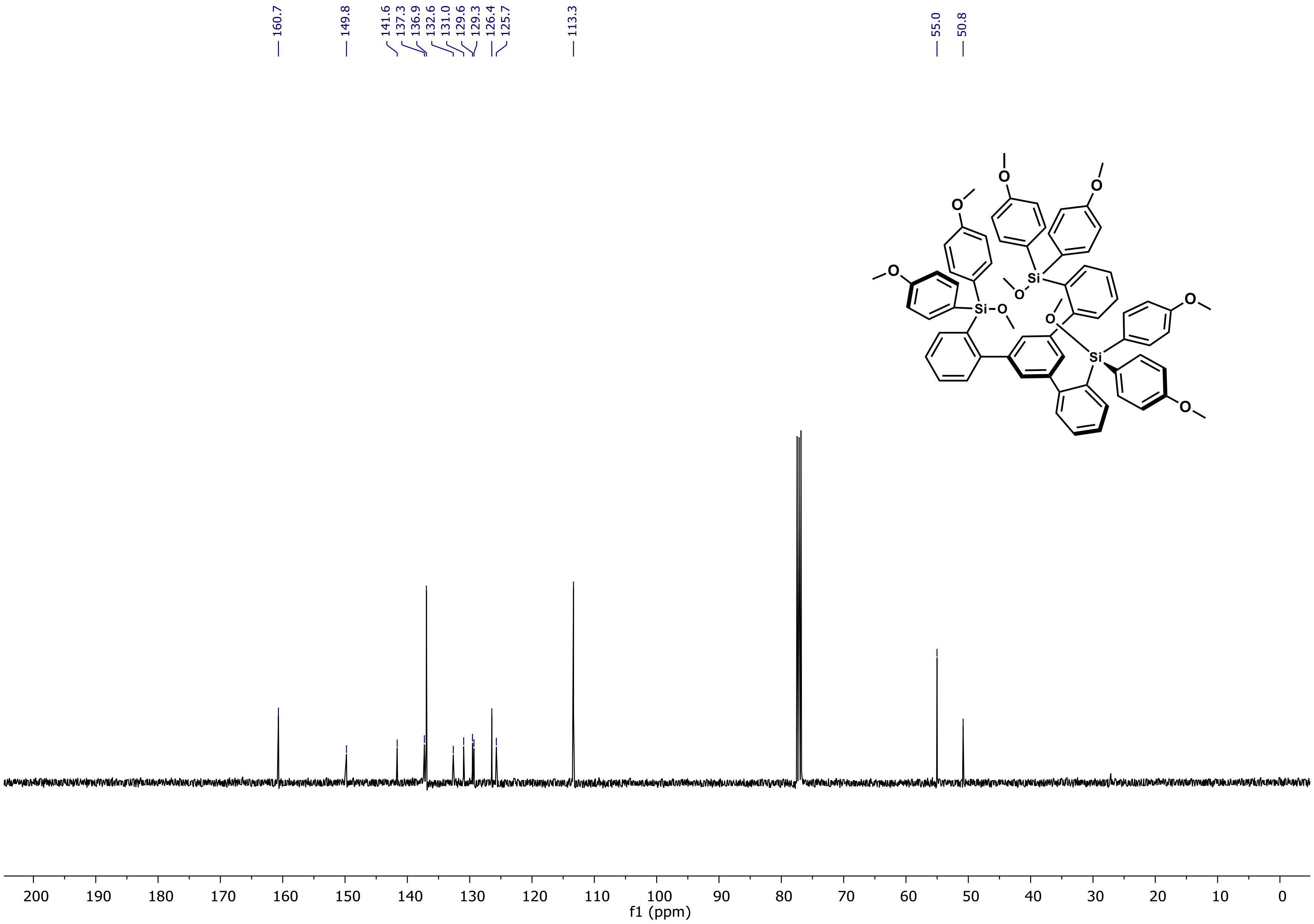

^{29}Si NMR of of Compound 12b, $CDCl_3$, 23 ºC

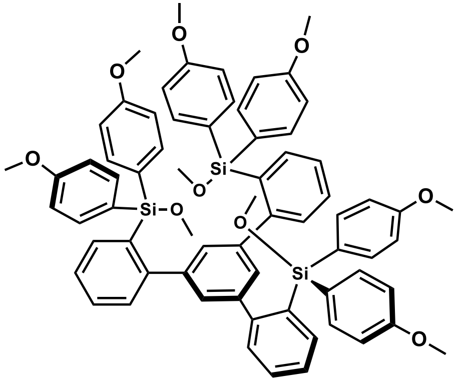

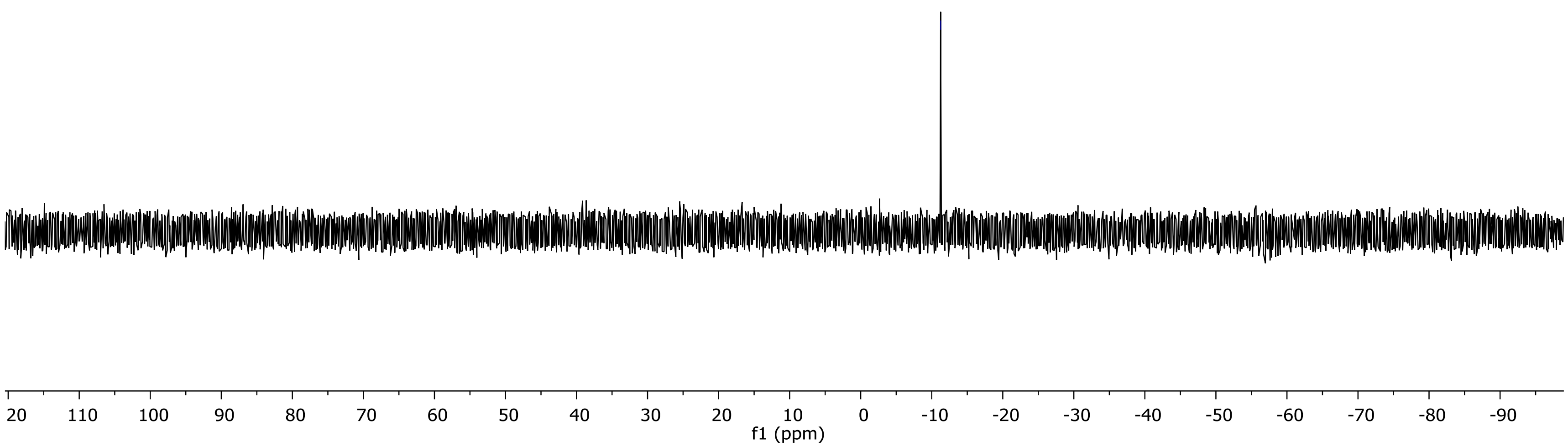

^{1}H NMR of Ligand 13b, $CDCl_3$, 23 ºC

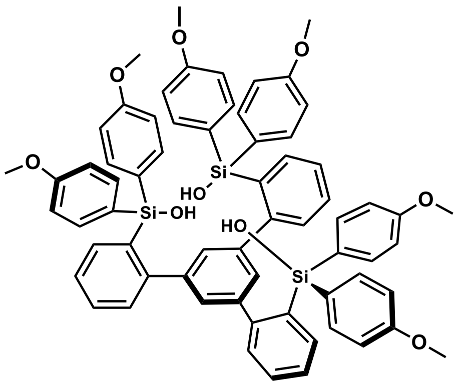

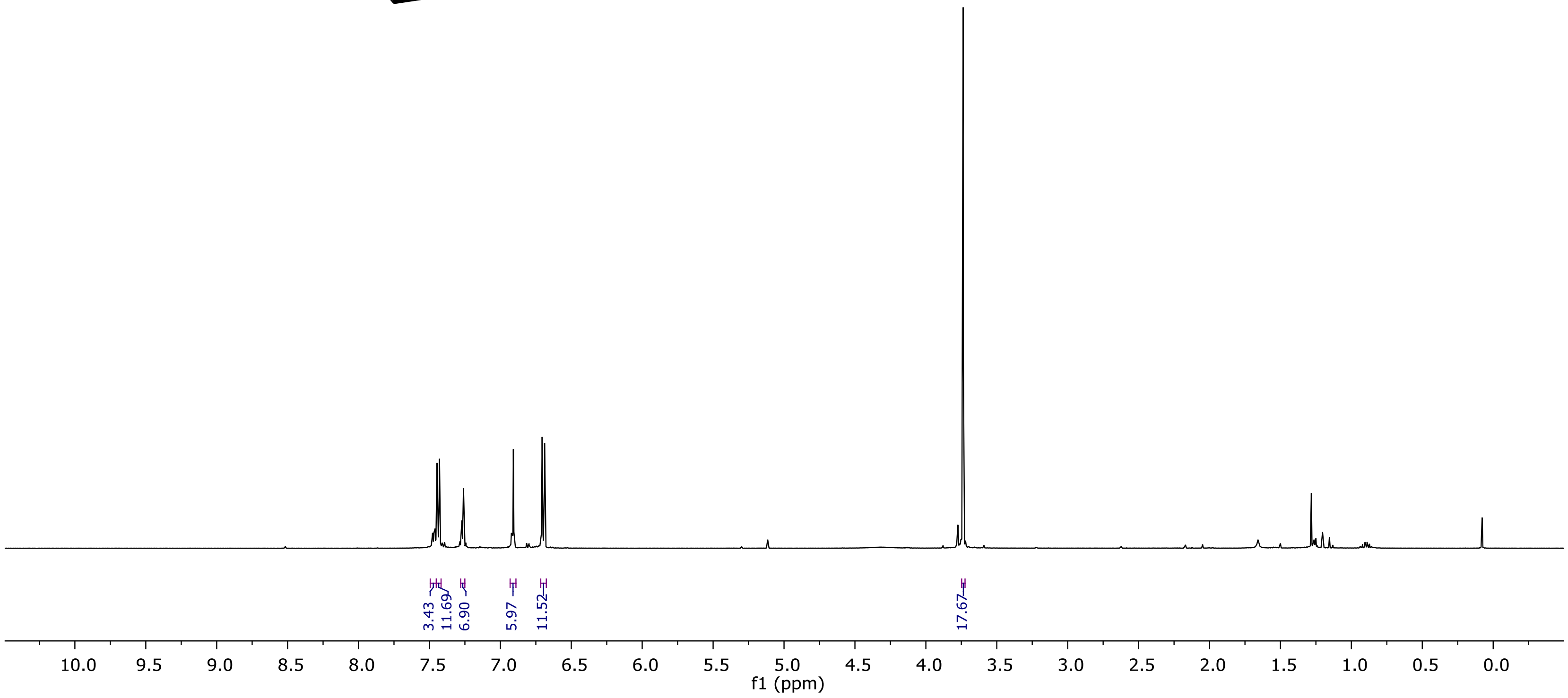

^{13}C NMR of Ligand 13b, $CDCl_3$, 23 ºC

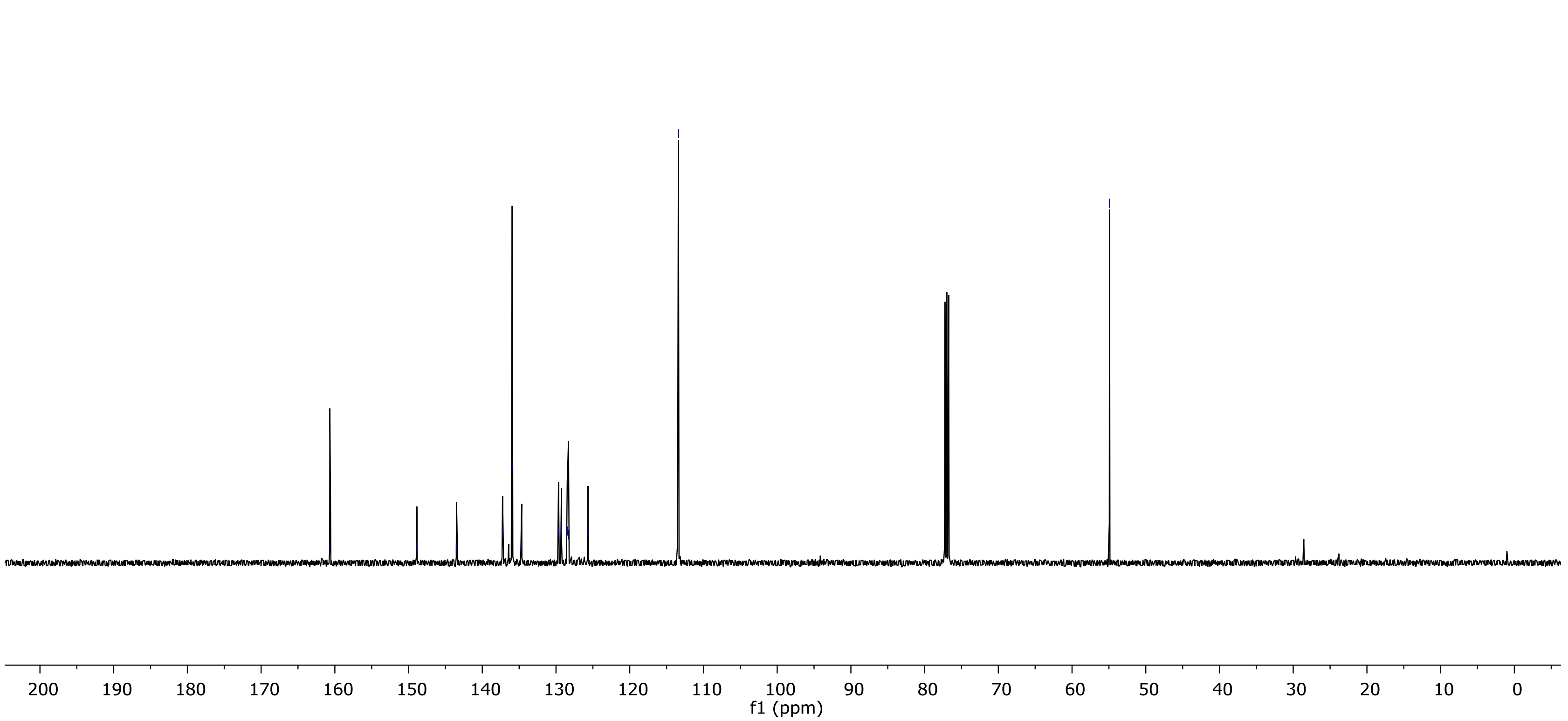

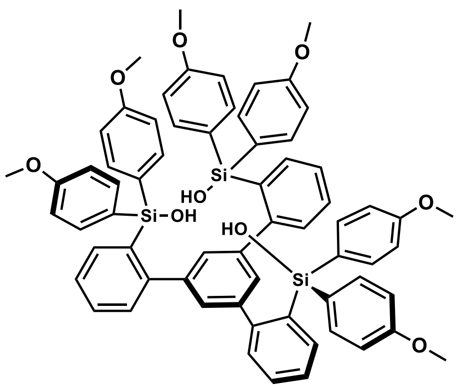

^{29}Si NMR of Ligand 13b, CDCl3, 23 ºC

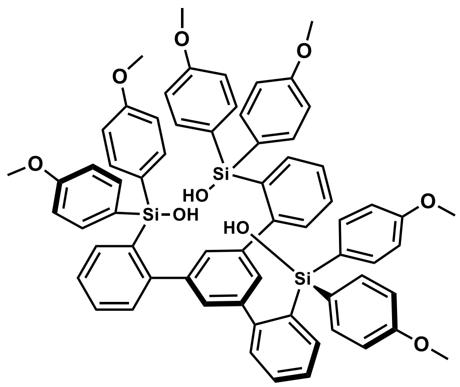

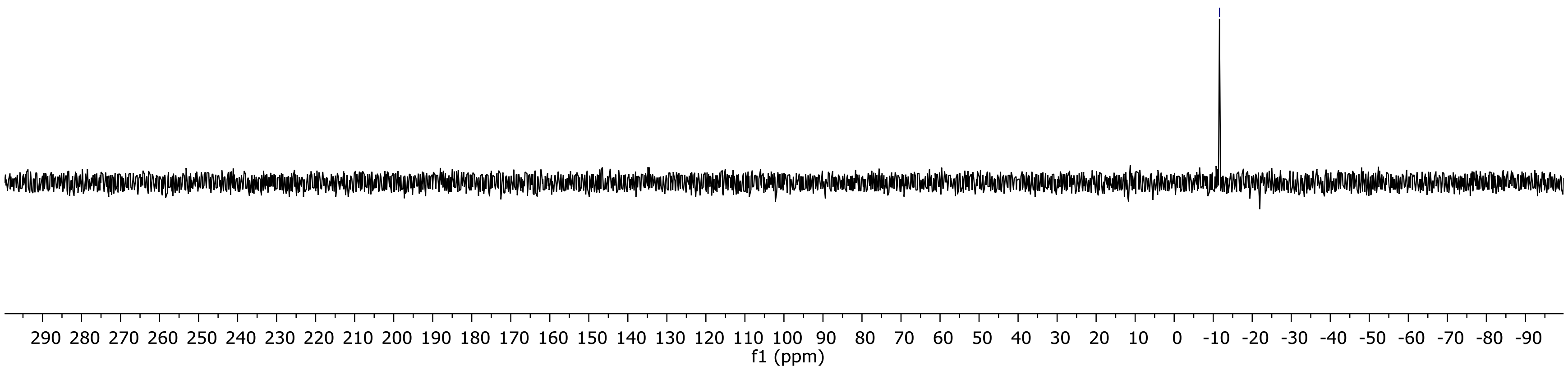

^{1}H NMR of Complex 15, C_6D_6, 23 ºC

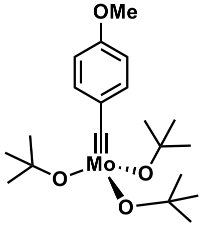

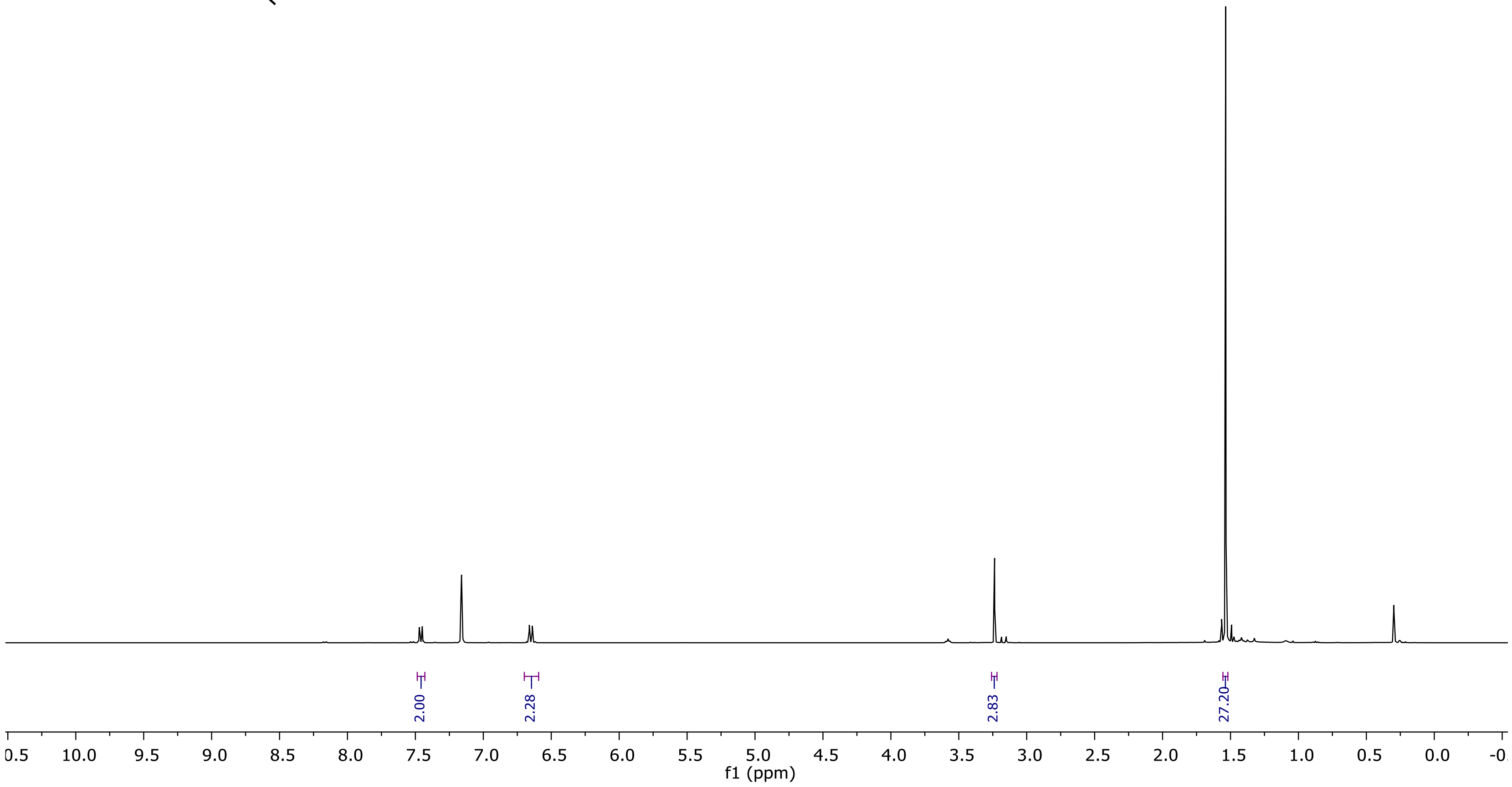

^{13}C NMR of Complex 15, C_6D_6, 23 ºC

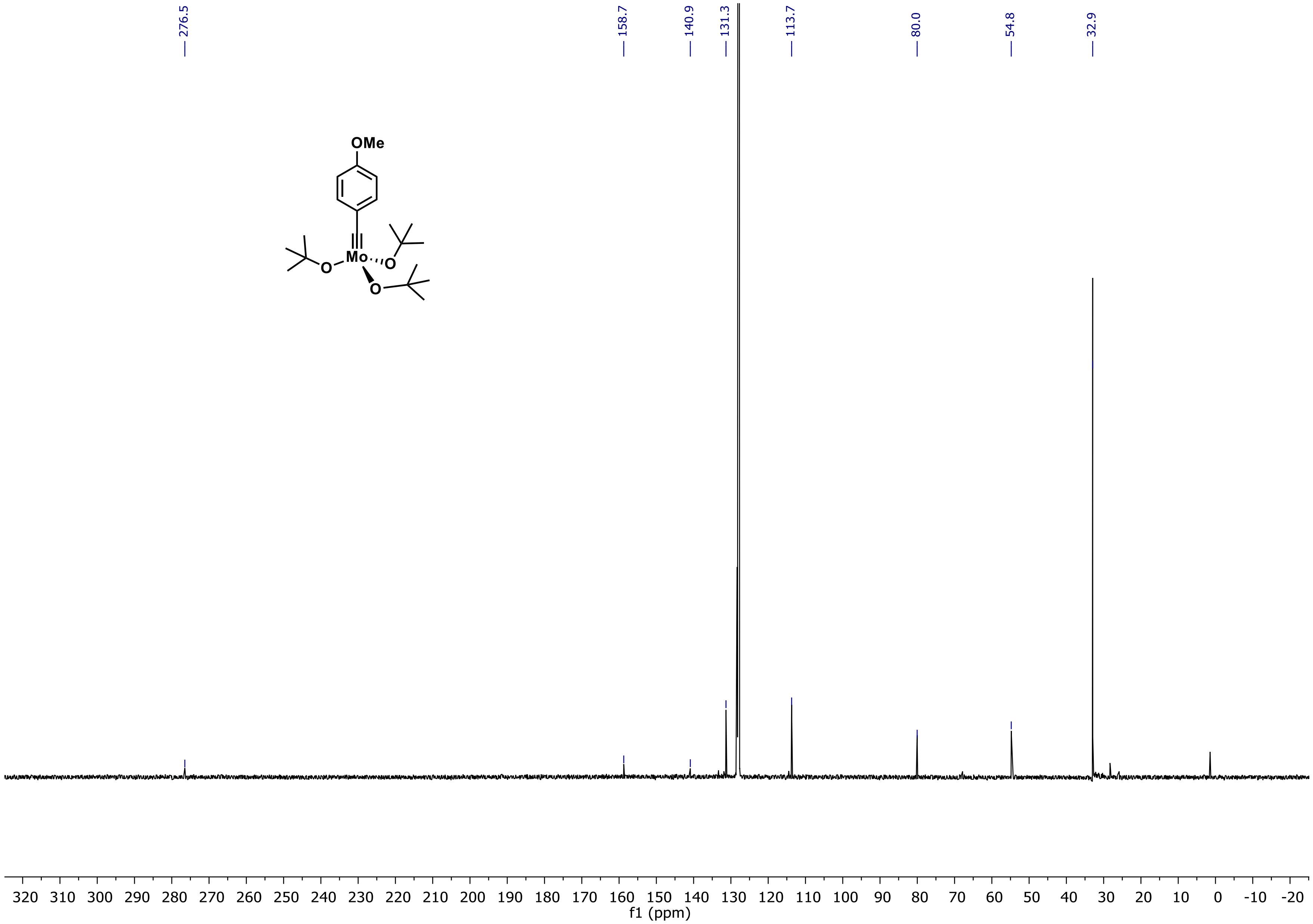

^{1}H NMR (600 MHz) of Complex [16a]$_2$, C_6D_6, 23 ºC

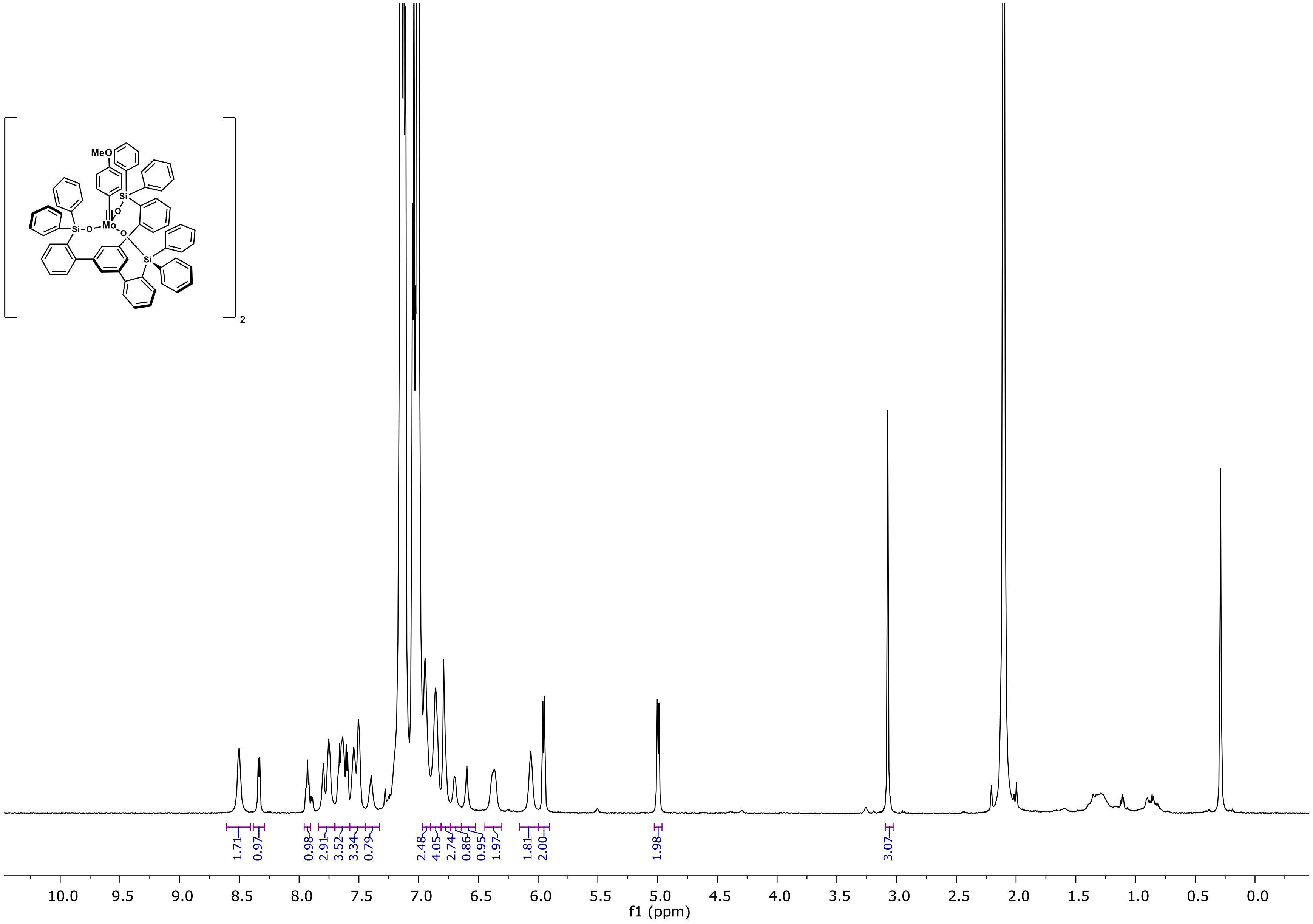

^{13}C NMR (150 MHz) of Complex [16a]$_2$, C_6D_6, 23 ºC

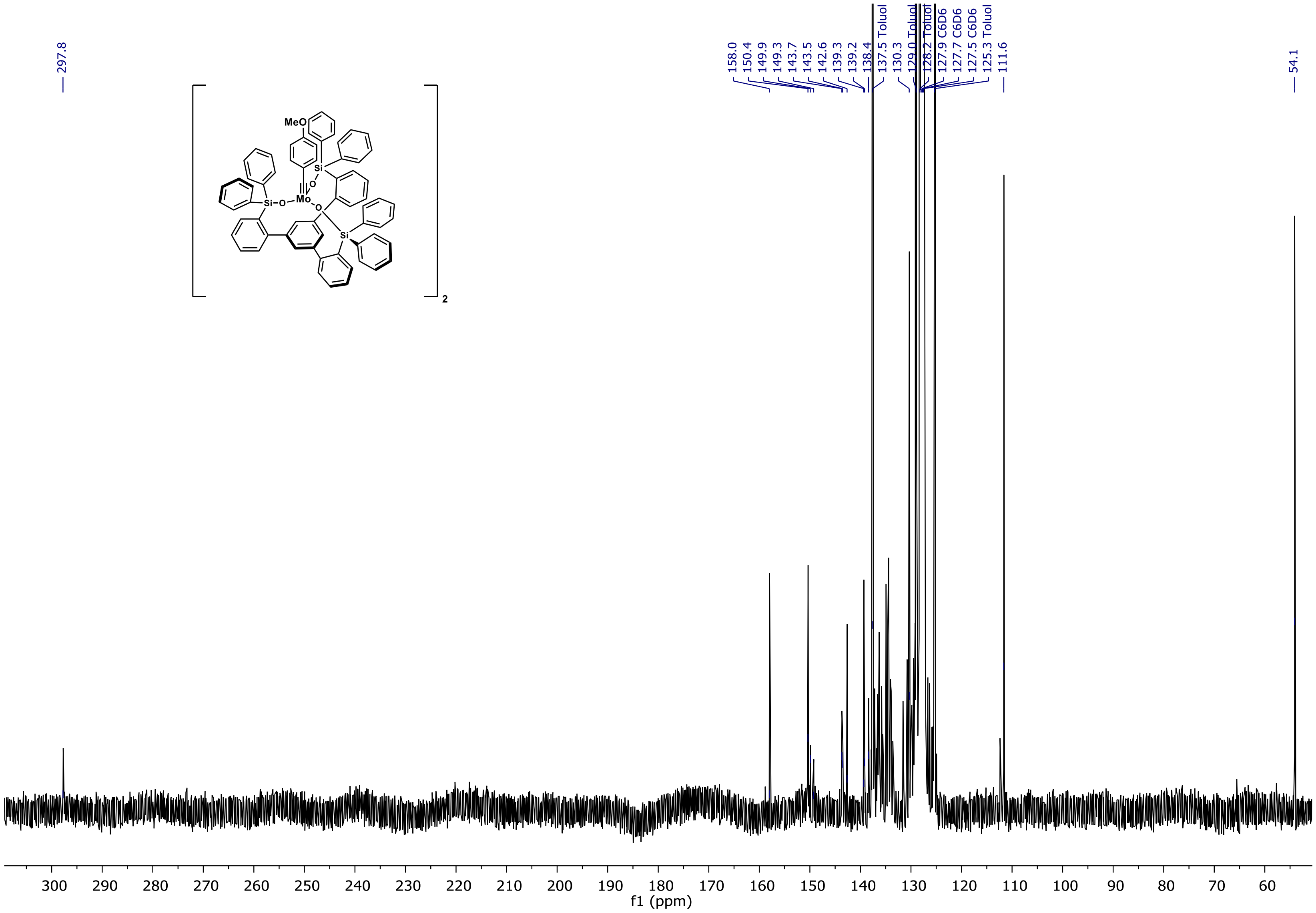

^{1}H-^{29}Si-HMBC NMR of Complex [16a]$_2$, C_6D_6, 23 ^{o}C

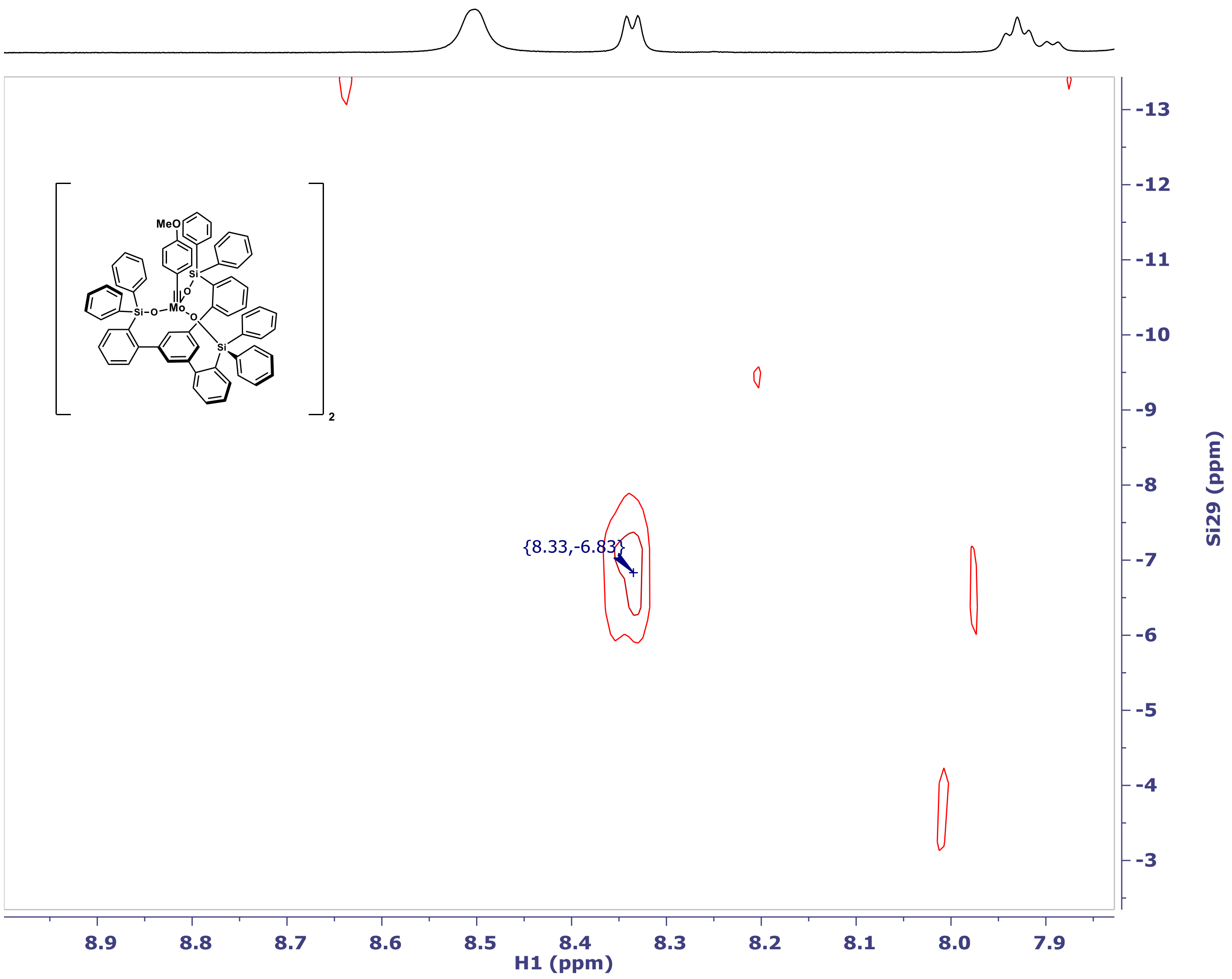

^{1}H NMR conversion studies of Complex [16a]$_2$ into Complex 16a, $C_6D_5CD_3$, 50 ºC

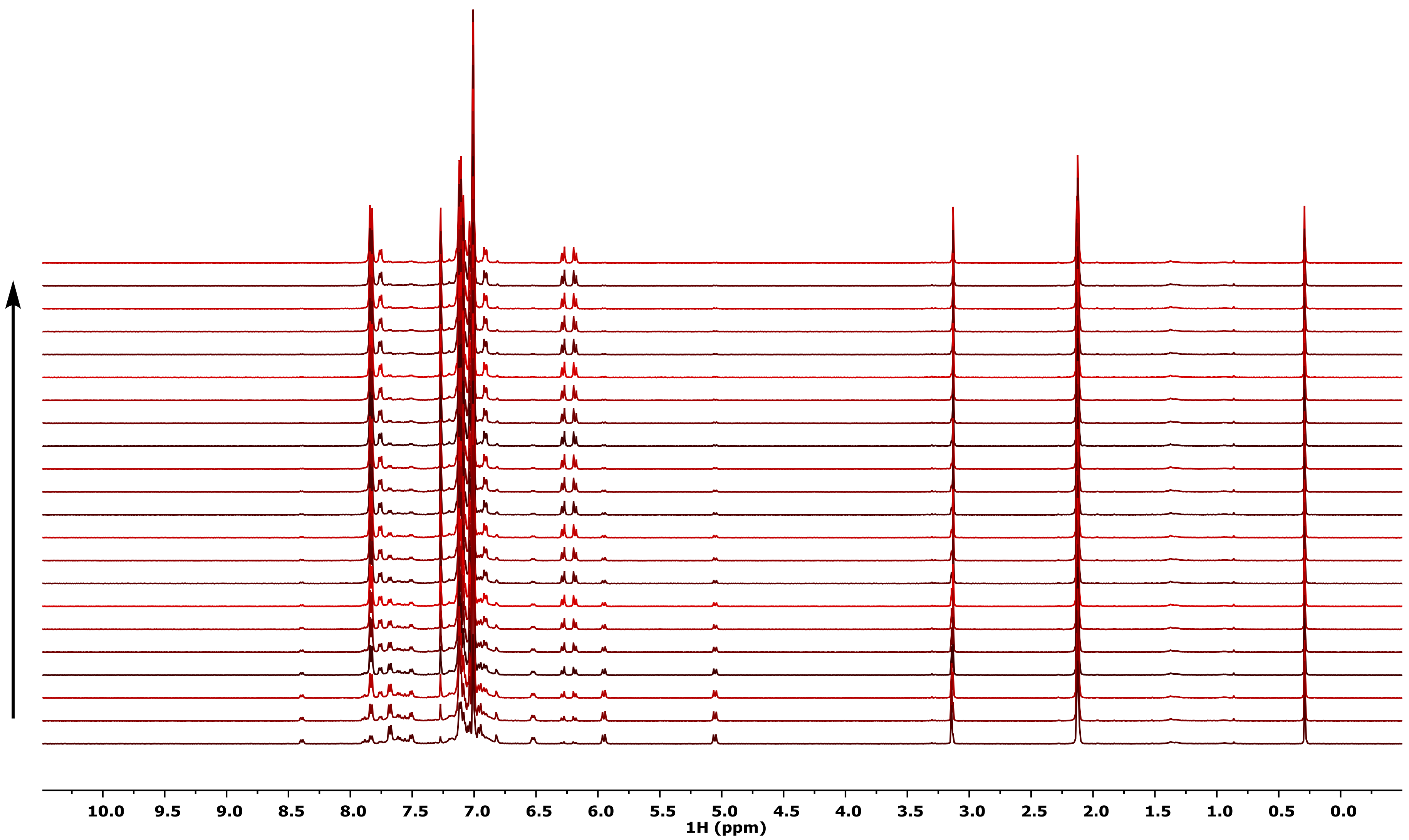

^{1}H NMR conversion studies of Complex [16a]$_2$ into Complex 16a, $C_6D_5CD_3$, 50 ºC

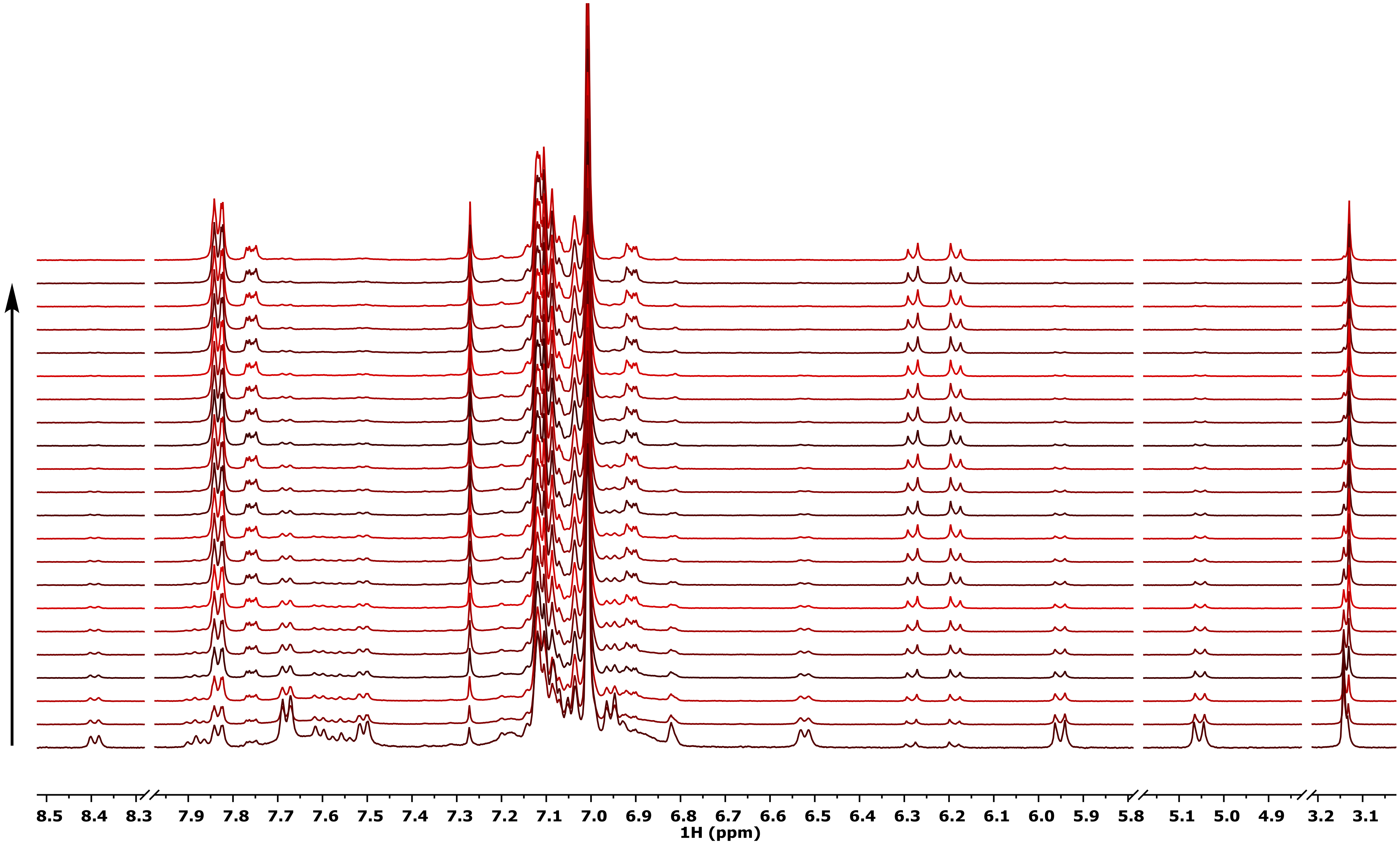

^{1}H-^{13}C-HMBC NMR: Alkylidyne Crosspeaks of [16a]$_2$ and [16a], $C_6D_5CD_3$, 23 °C

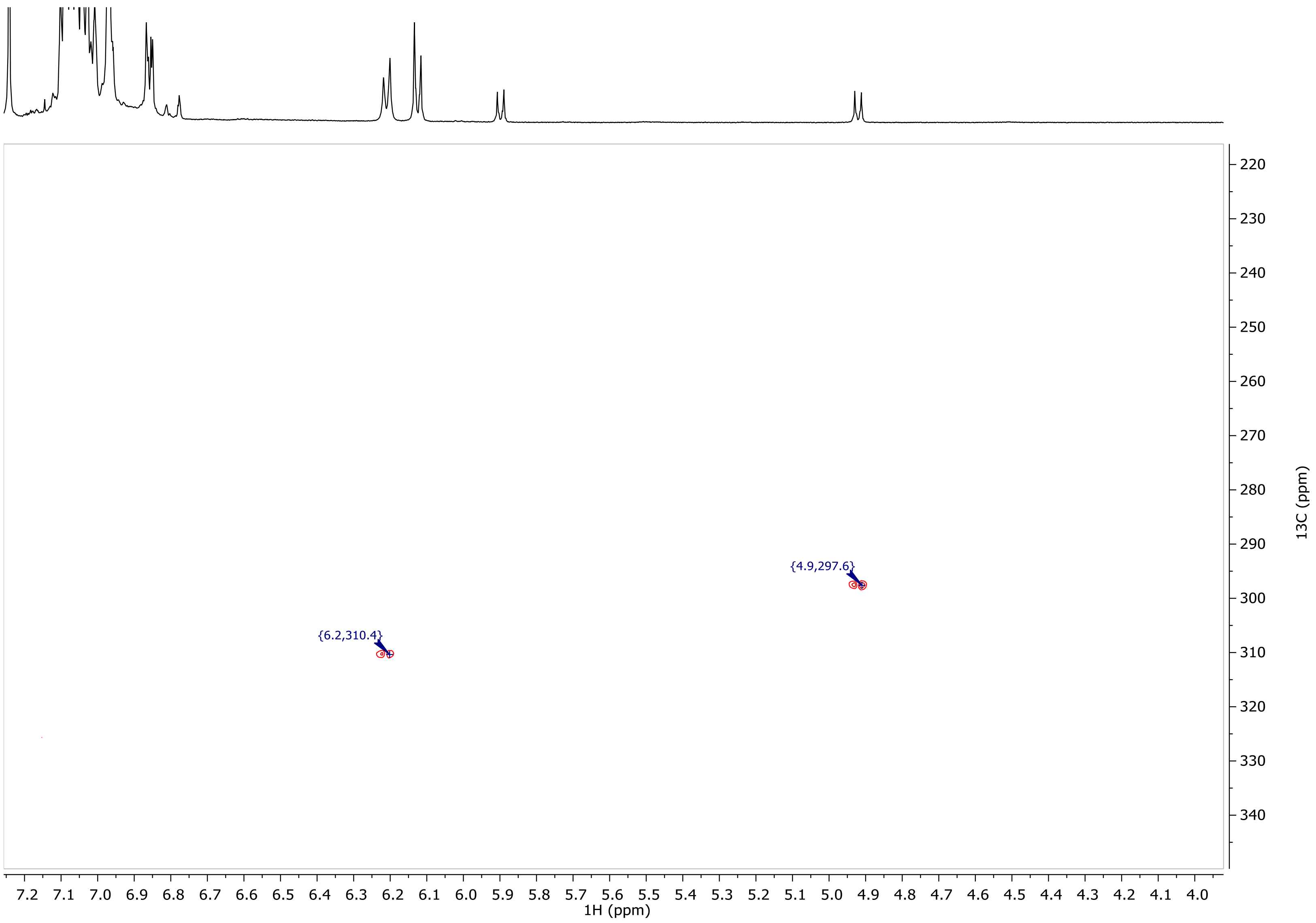

^{1}H NMR of Complex 16a (monomer), $C_6D_5CD_3$, 23 ºC

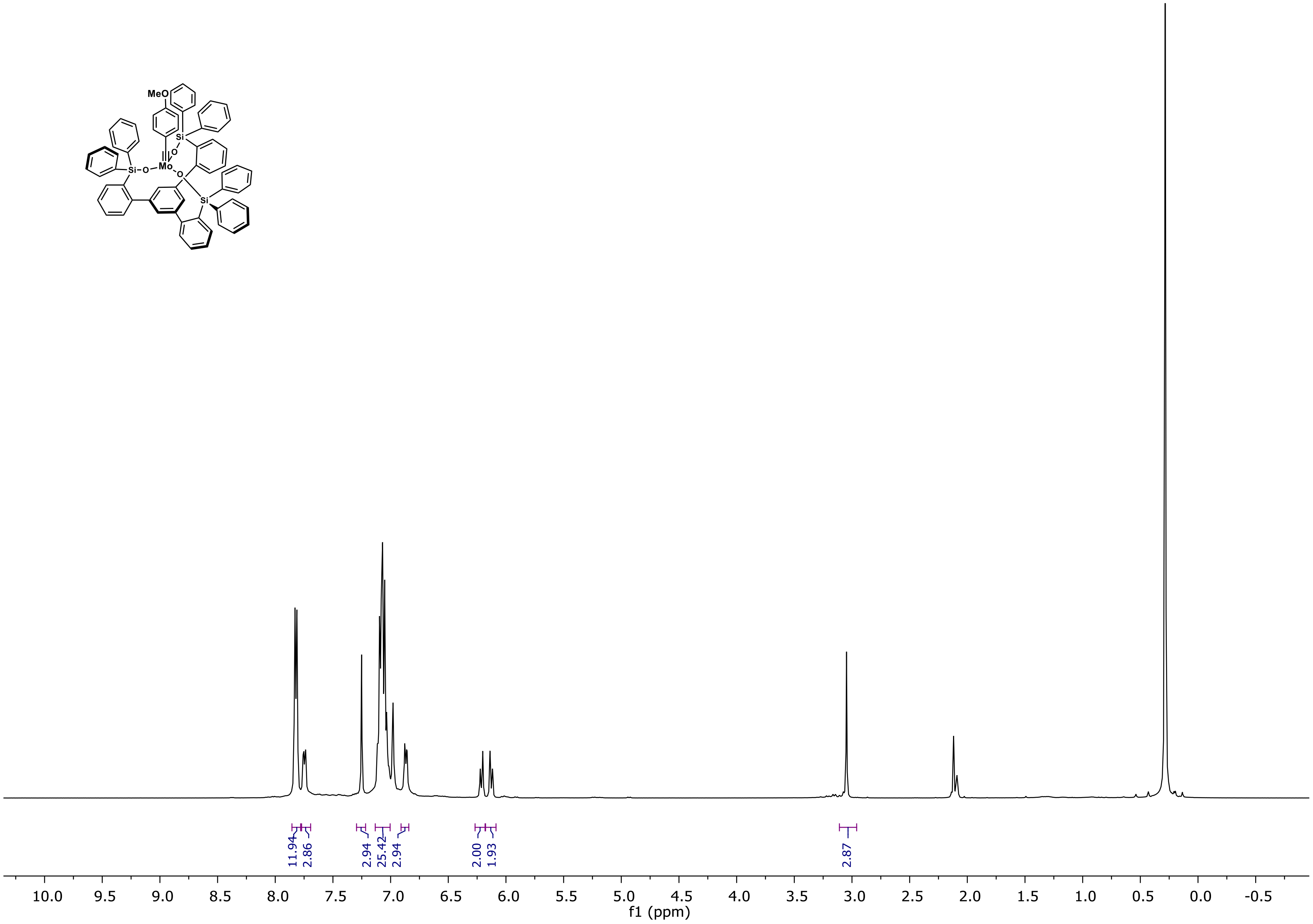

^{13}C NMR of Complex 16a (monomer), $C_6D_5CD_3$, 23 ºC

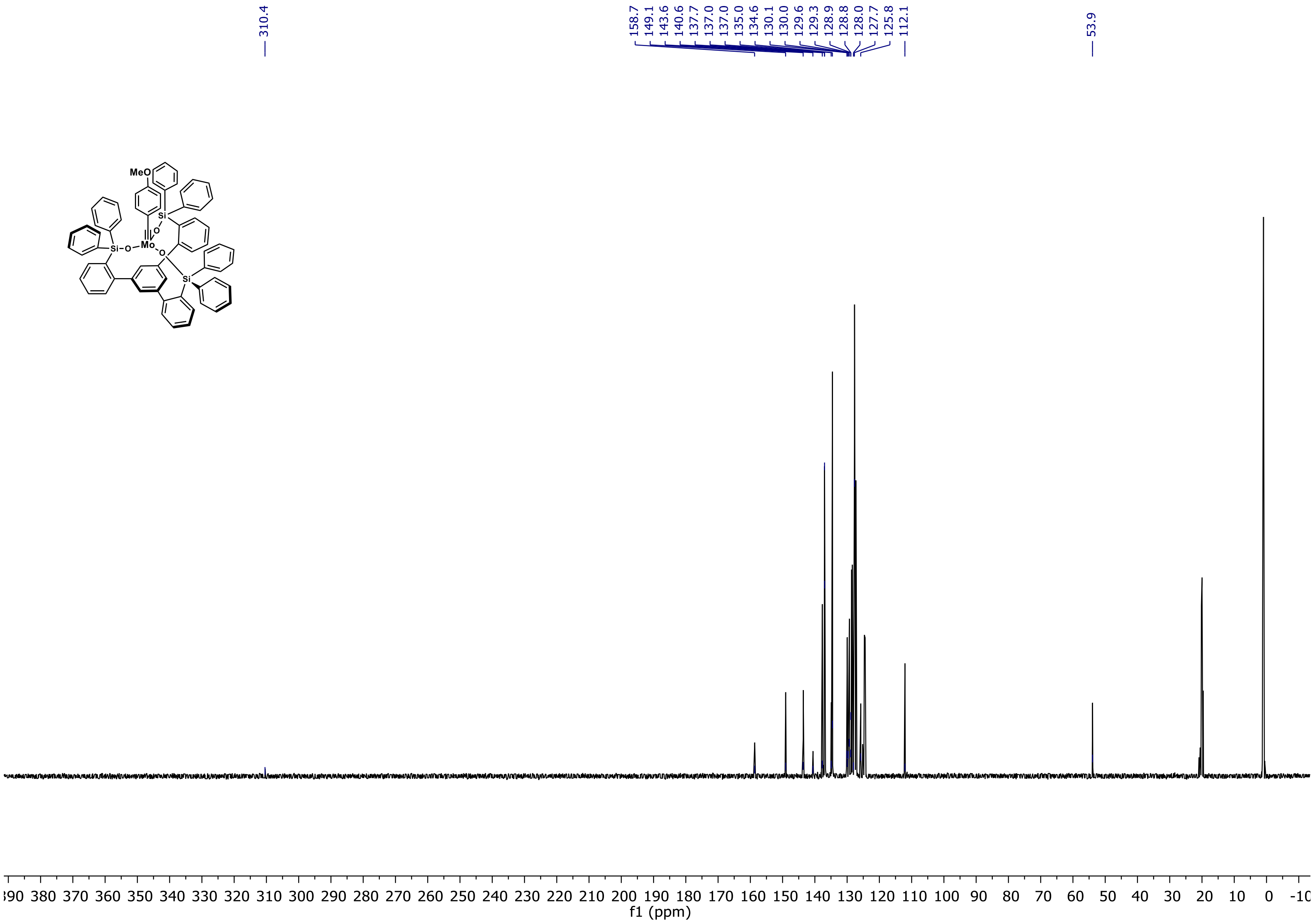

^{1}H-^{29}Si-HMBC NMR of Complex 16a (monomer), $C_6D_5CD_3$, 23 ^{o}C

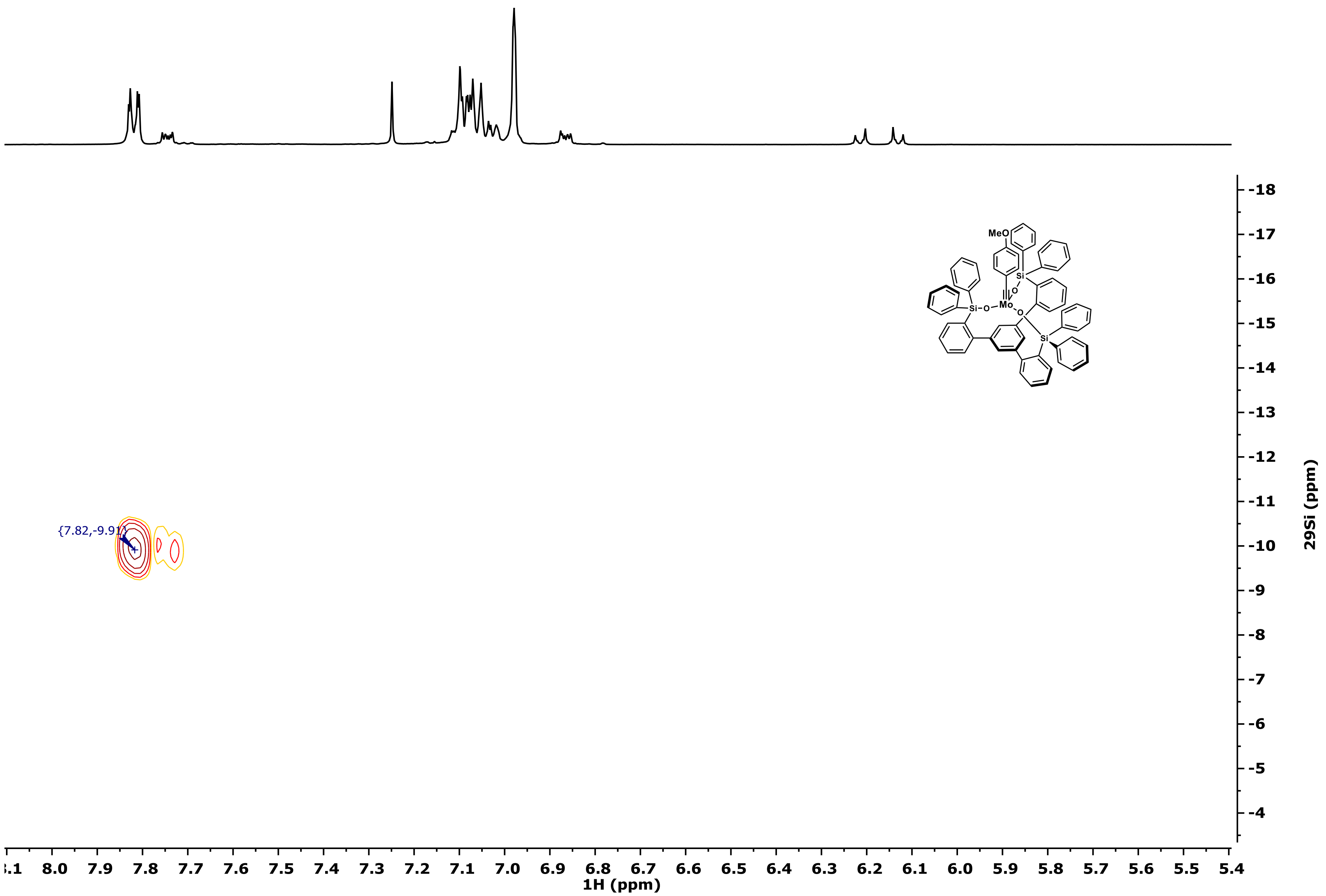

^{95}Mo NMR of Complex 16a (monomer), $C_6D_5CD_3$, 60 ºC

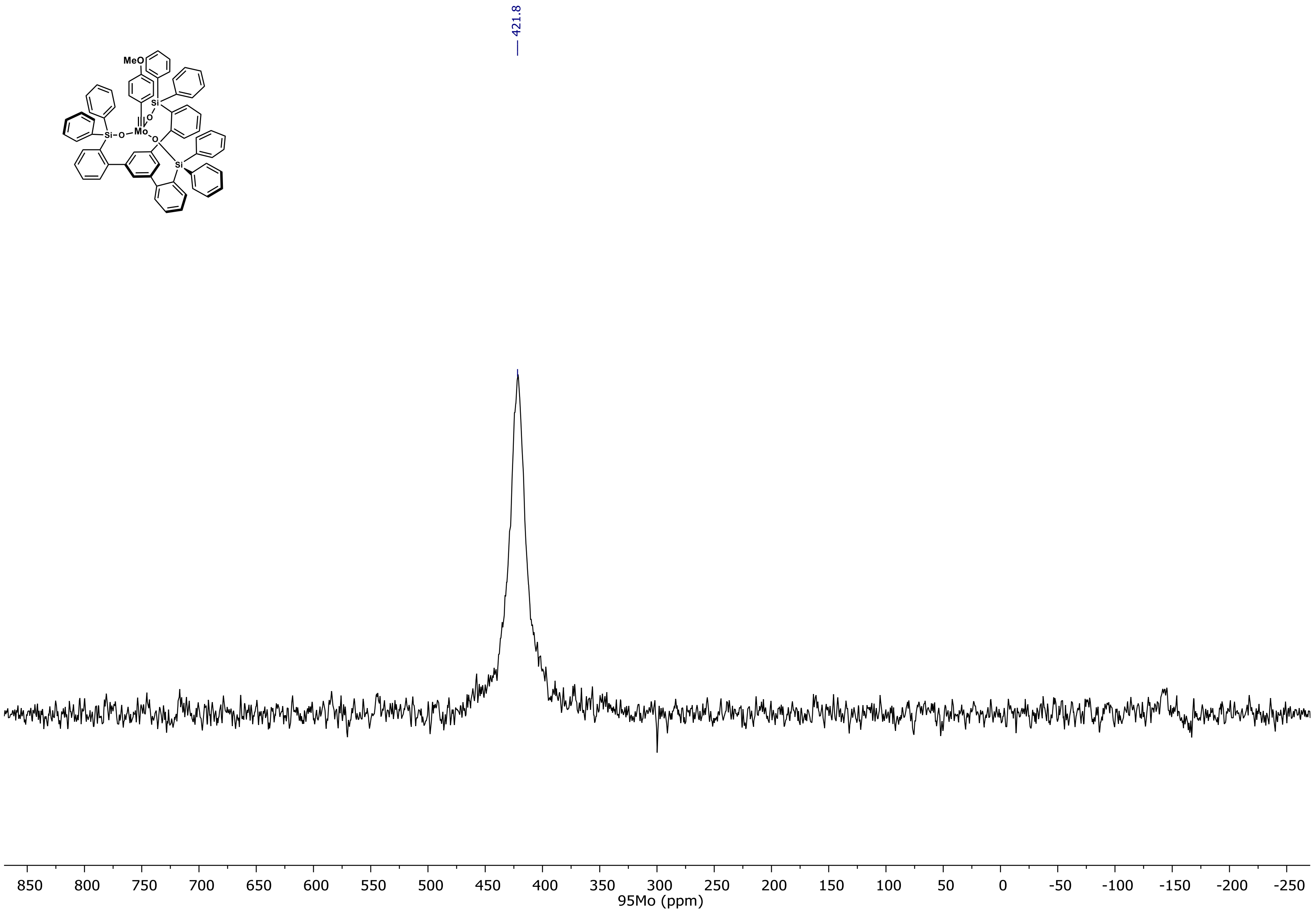

^{1}H NMR conversion studies of Complex [16a)$_2$ into Complex 16a·MeCN, $C_6D_5CD_3$, 60 ºC

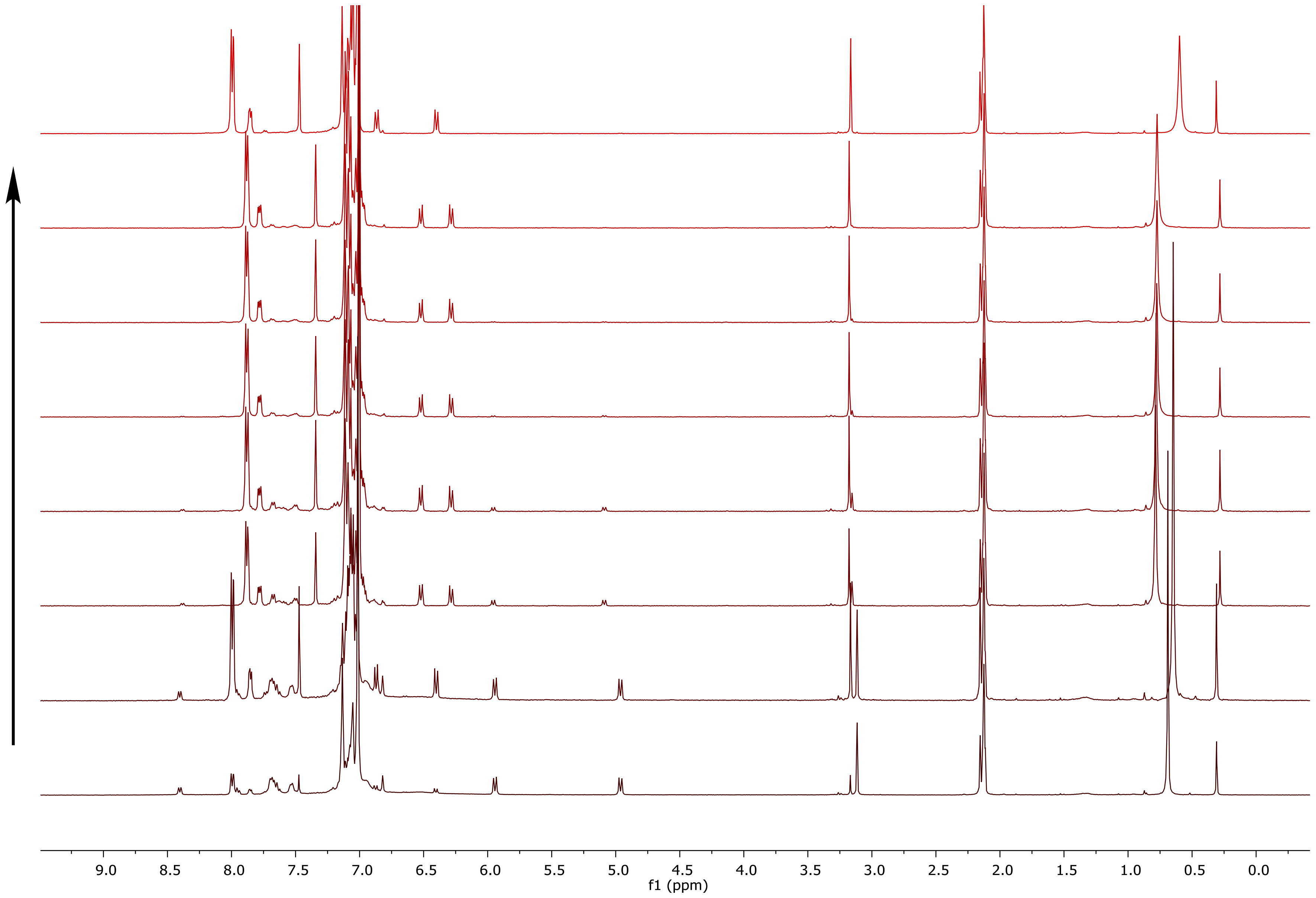

^{1}H NMR of Complex 16a·MeCN, $C_6D_5CD_3$, 23 ºC

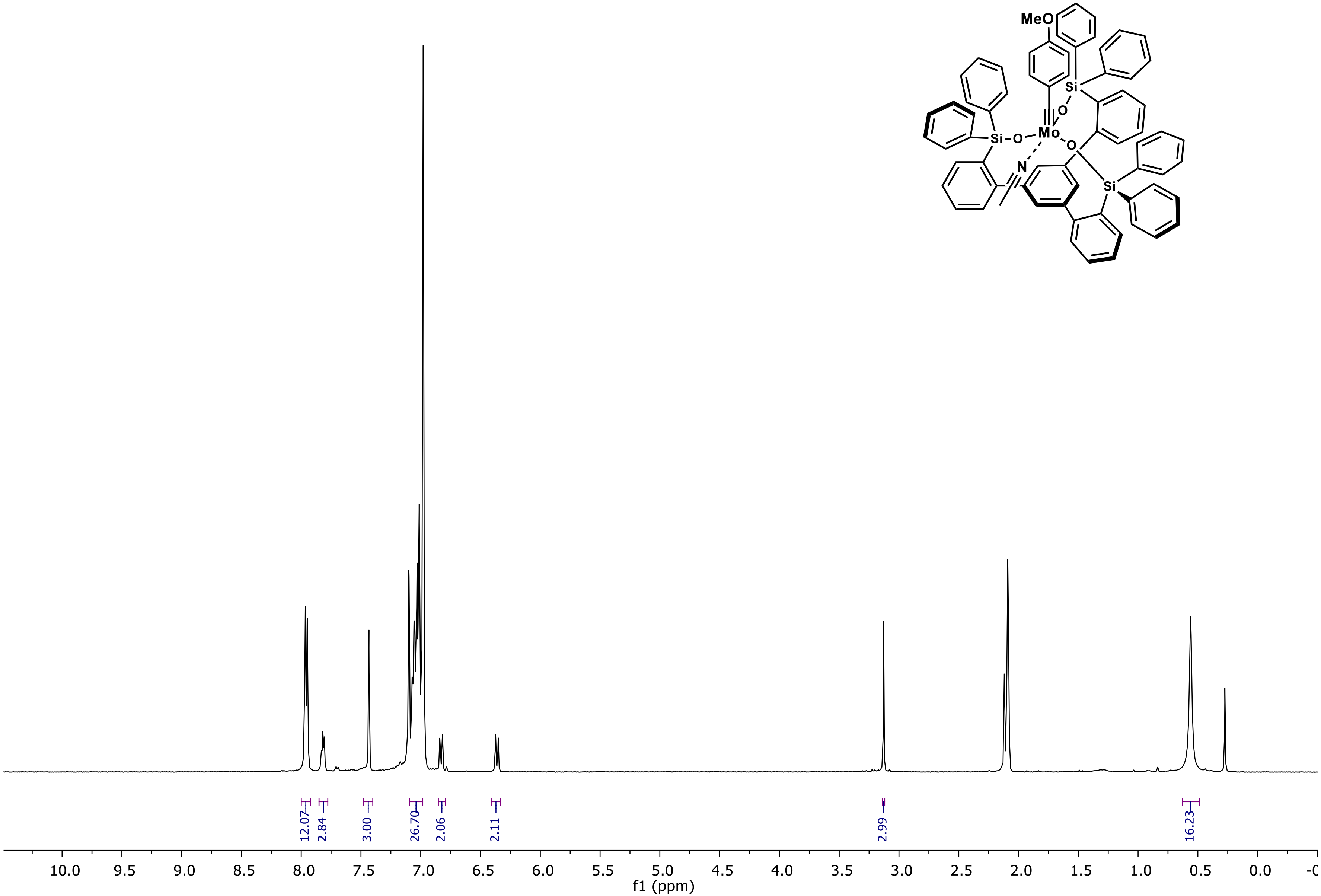

^{1}H NMR conversion studies of Complex [16b]$_2$ to Complex 16b, $C_6D_5CD_3$, 60 ^{o}C

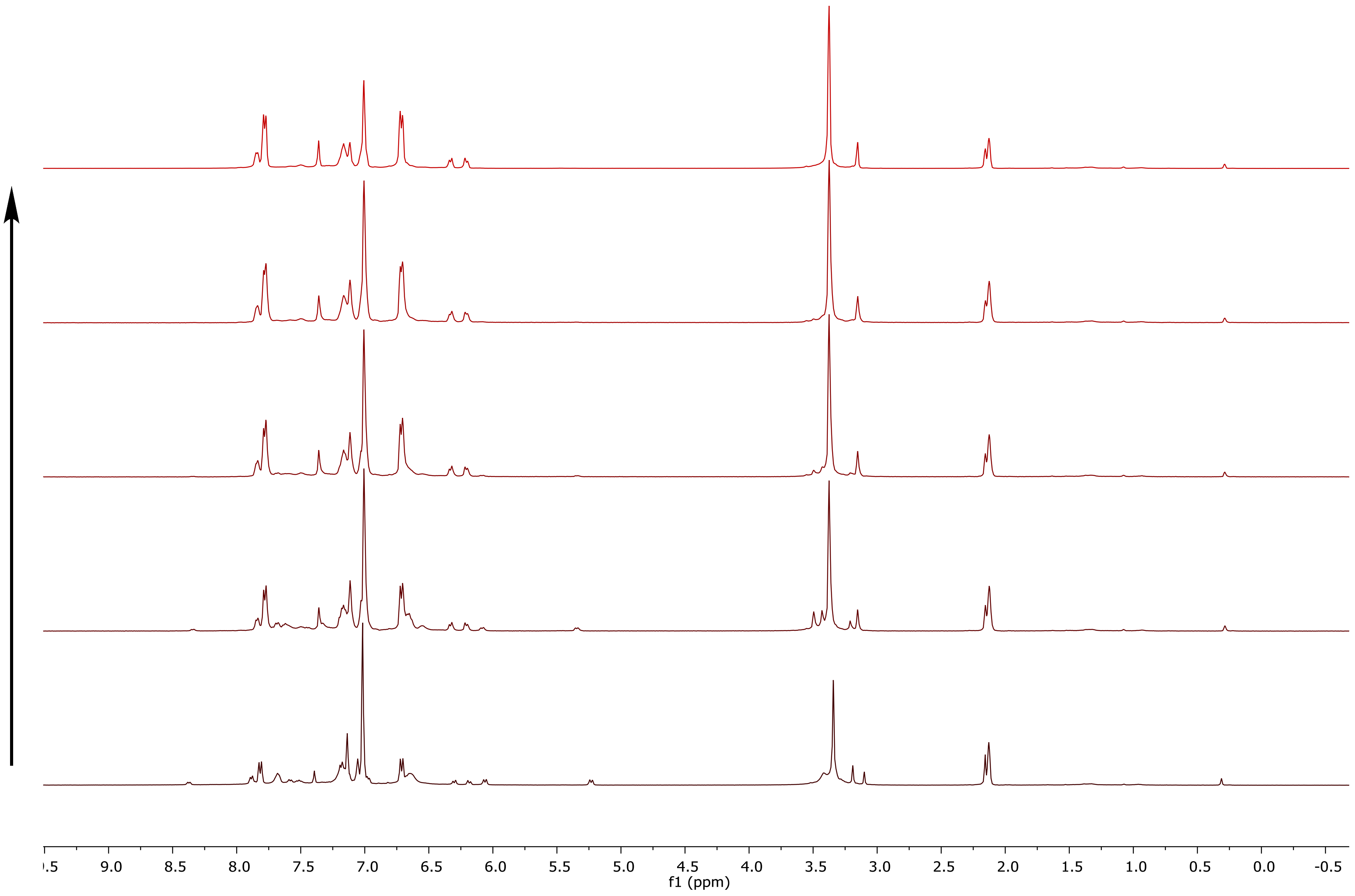

^{1}H NMR conversion studies of Complex Complex [16b]$_2$ to Complex 16b, $C_6D_5CD_3$, 60 ºC

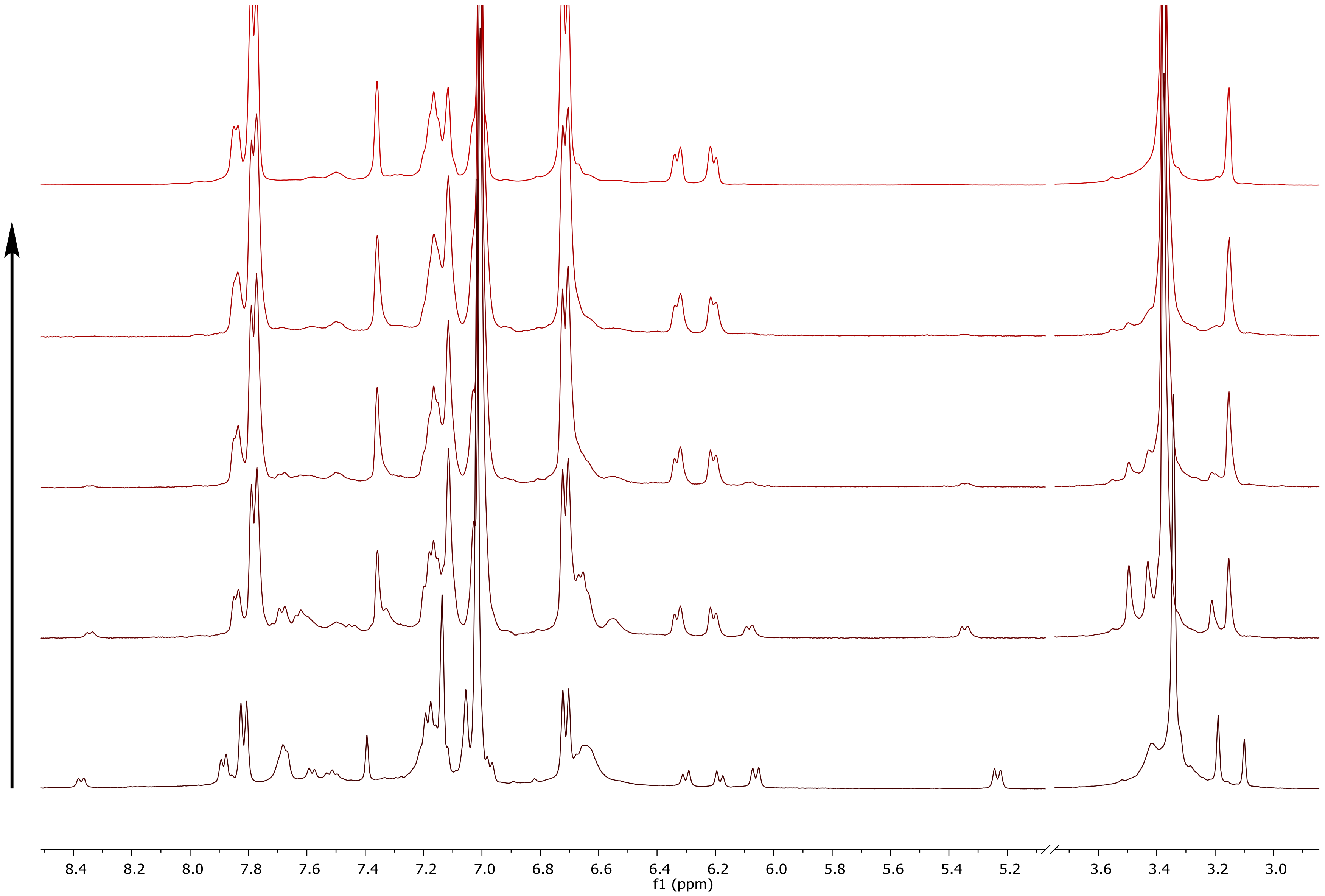

^{1}H NMR of Complex 16b (monomer), $C_6D_5CD_3$, 23 ºC

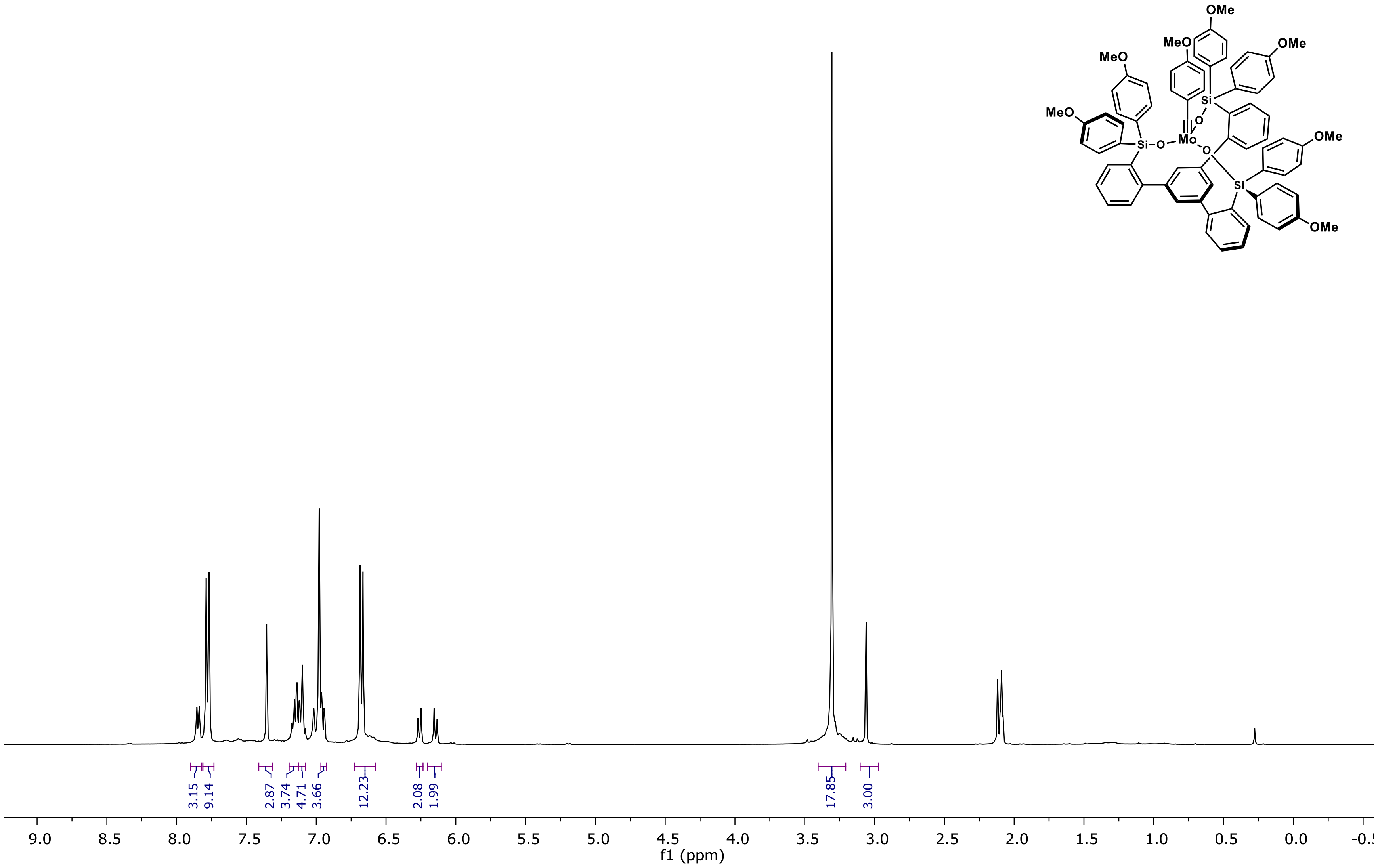

^{1}H NMR of Complex 16b (monomer), $C_6D_5CD_3$, 23 ºC

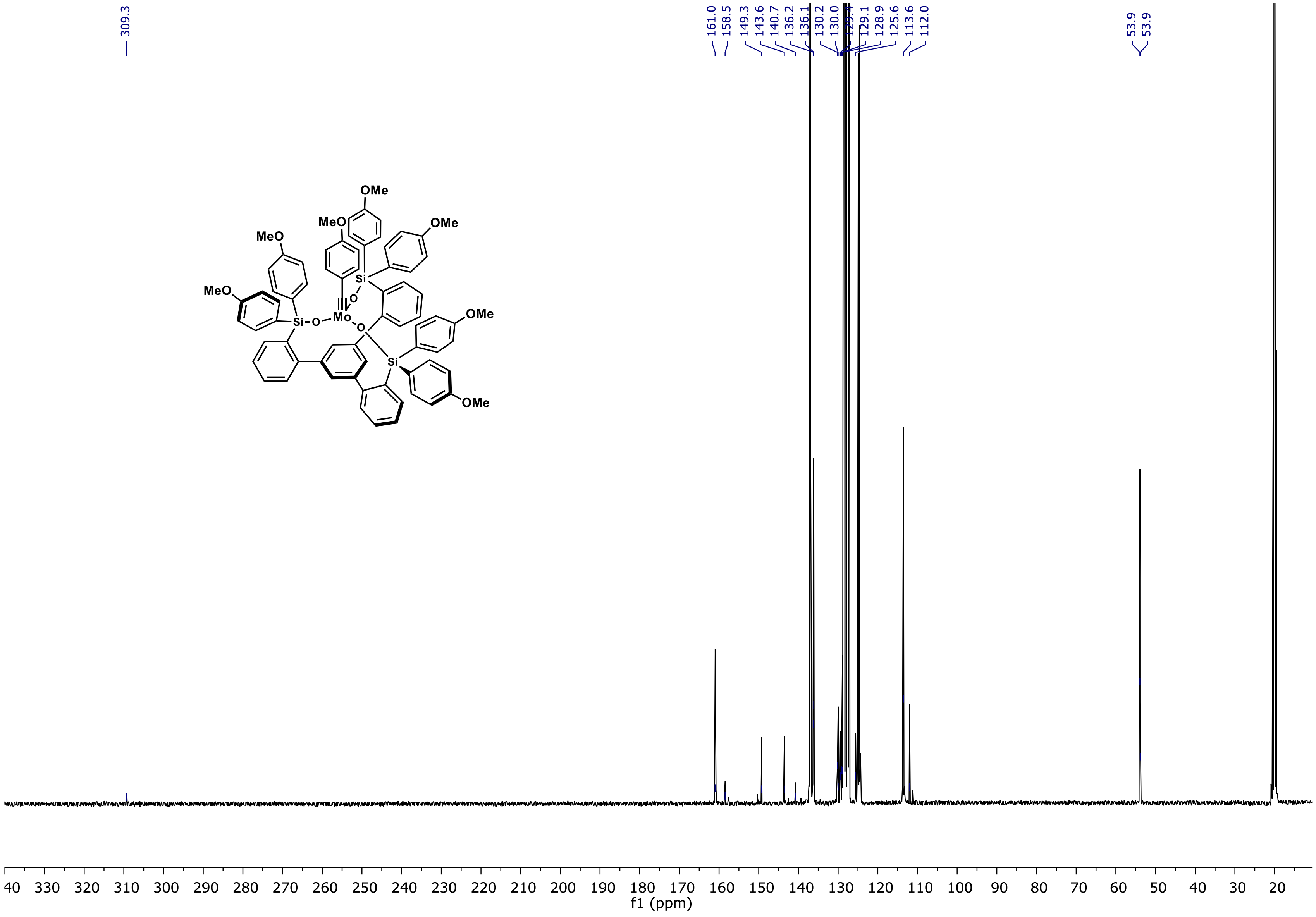

^{29}Si NMR of Complex 16b (monomer), $C_6D_5CD_3$, 23 °C

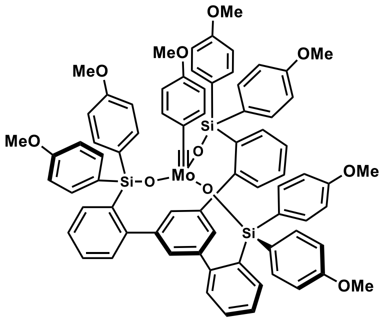

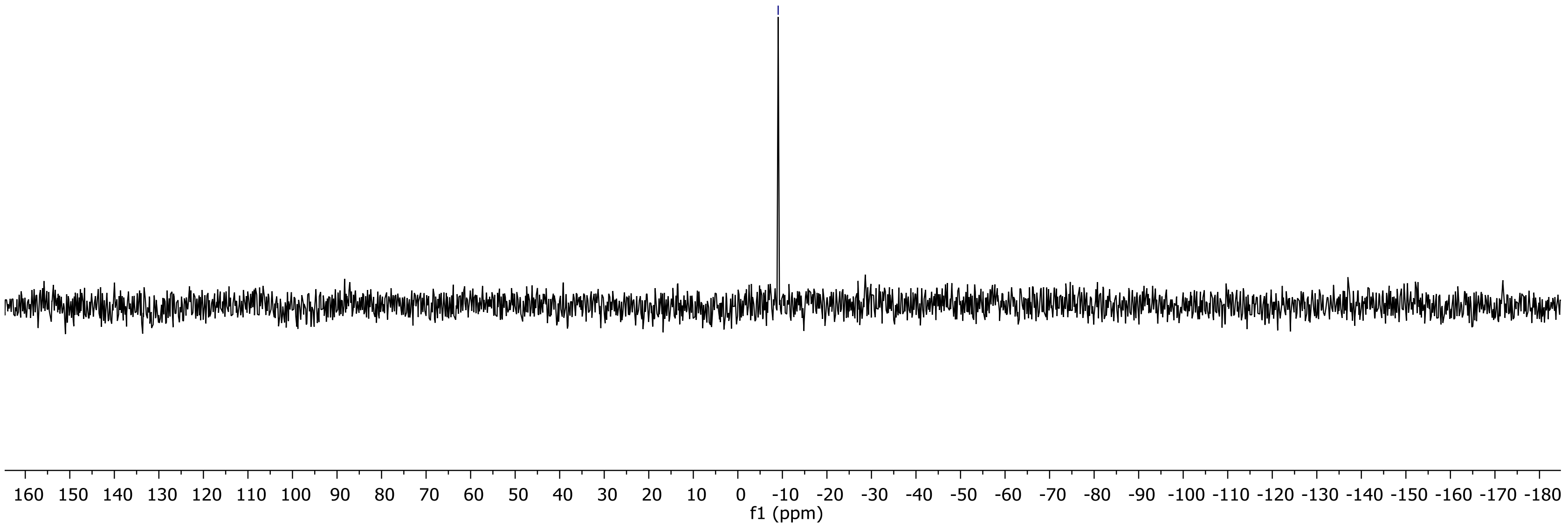

^{95}Mo NMR of Complex 16b (monomer), $C_6D_5CD_3$, 60 ºC

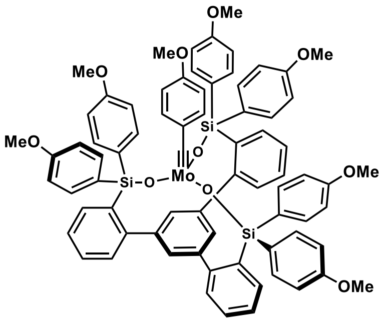

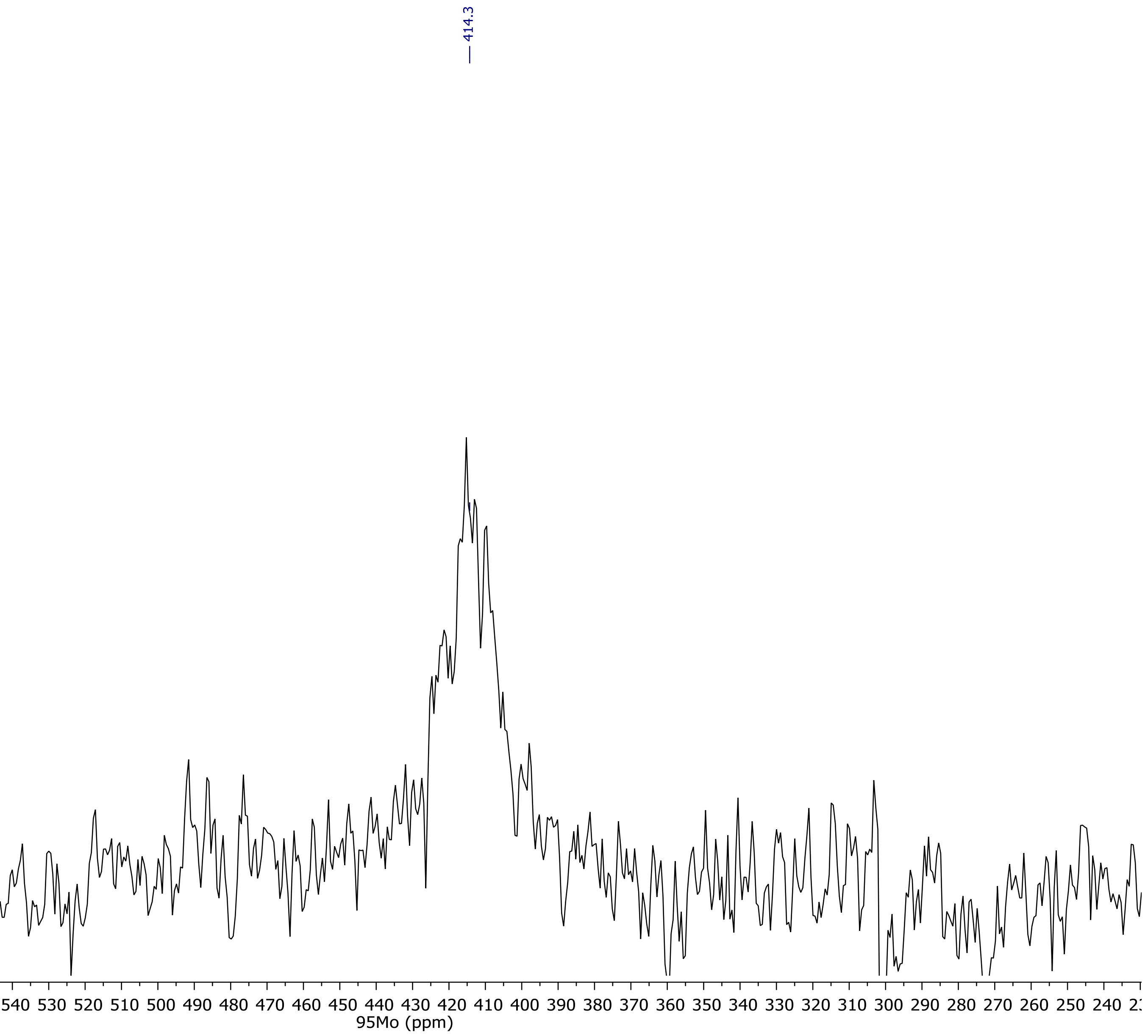

^{1}H NMR of Complex 1, $C_6D_5CD_3$, 23 ºC

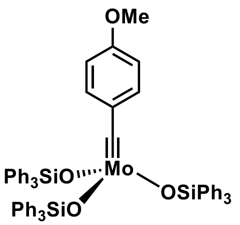

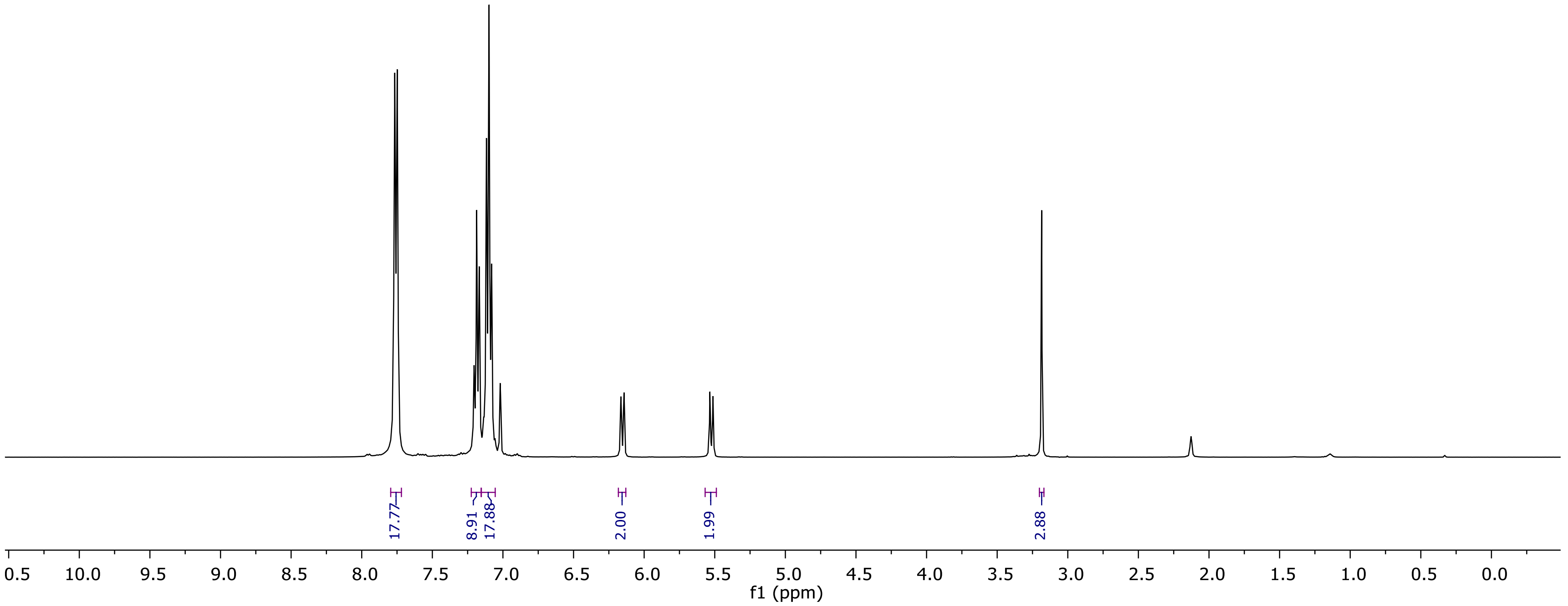

^{13}C NMR of Complex 1, $C_6D_5CD_3$, 23 ºC

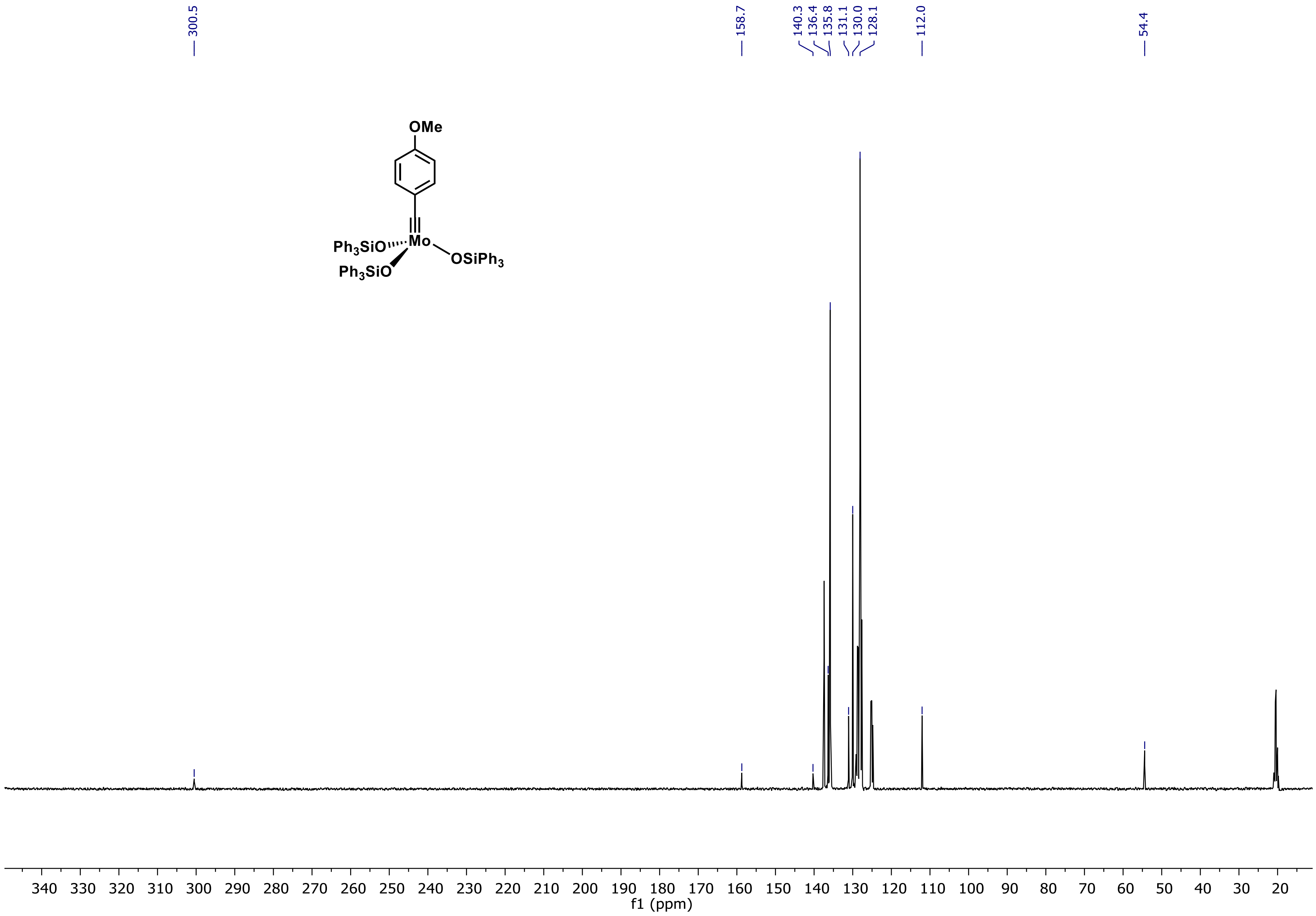

^{29}Si NMR of Complex 1, $C_6D_5CD_3$, 23 ºC

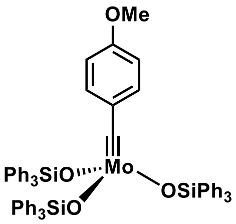

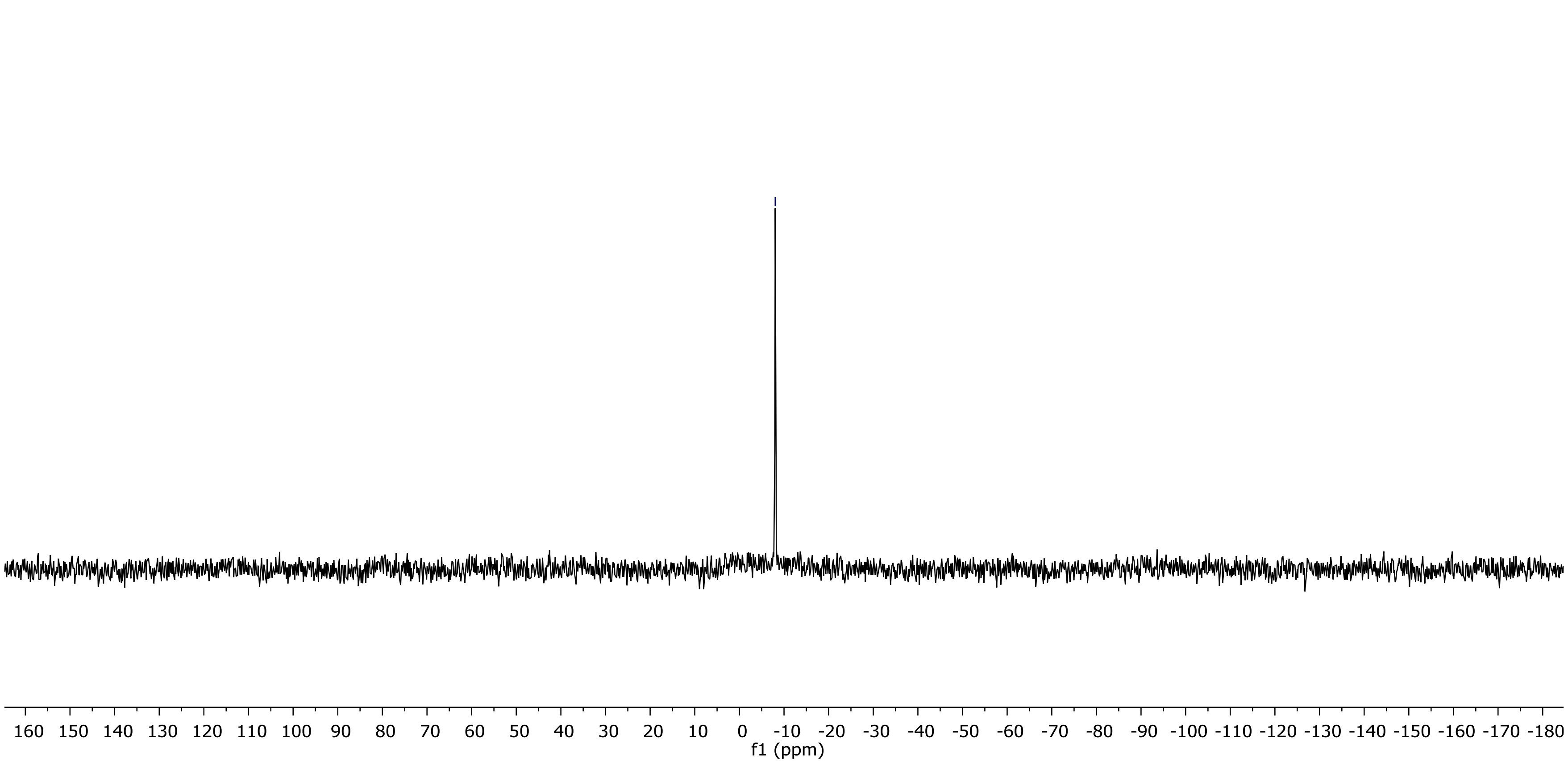

^{95}Mo NMR of Complex 1, $C_6D_5CD_3$, **23 ºC**

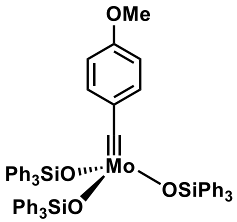

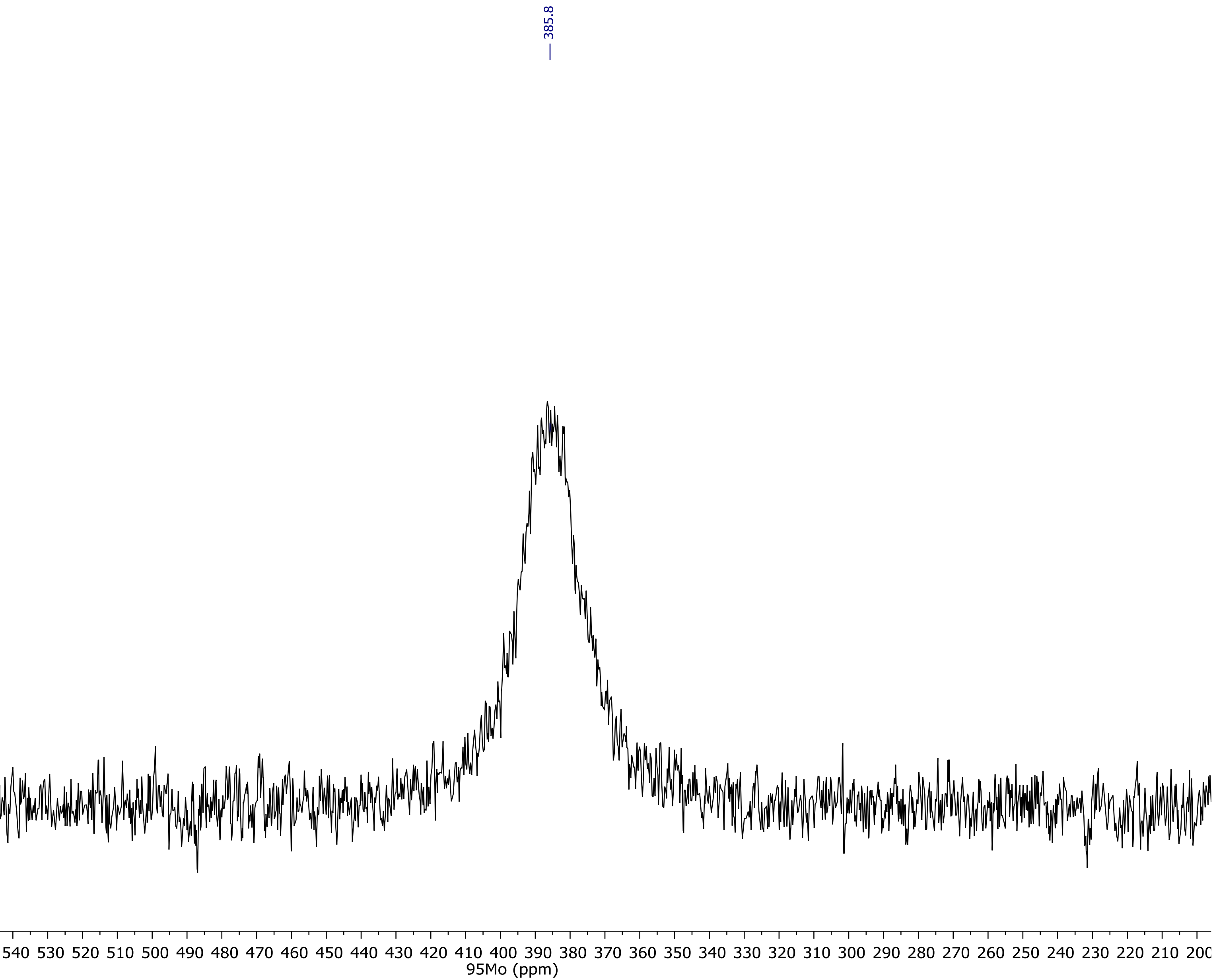

^{95}Mo NMR of Complex 1, $C_6D_5CD_3$, **60 ºC**

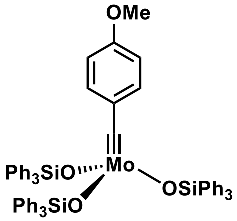

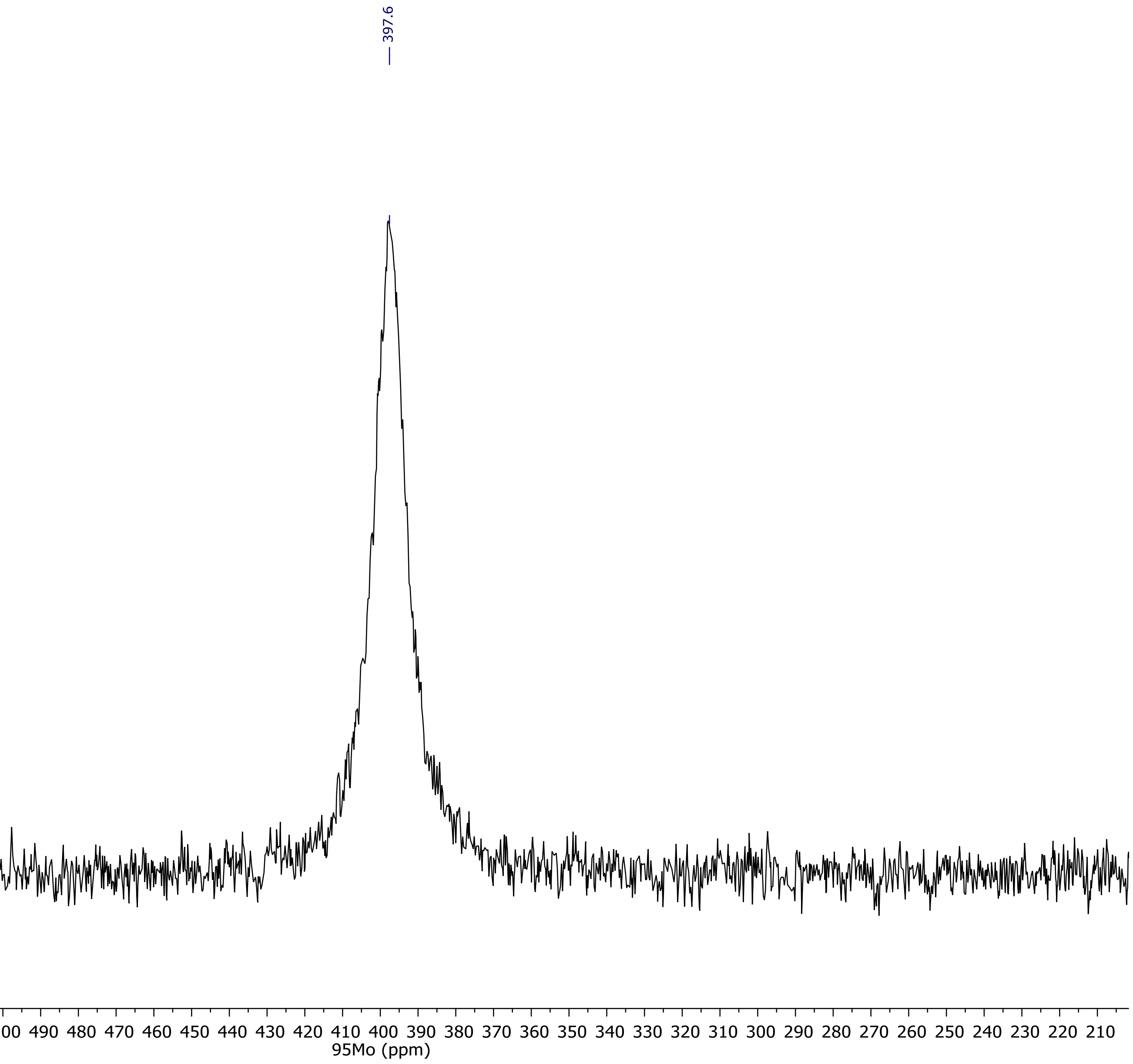

^{1}H NMR of Octadec-9-yne-1,18-diol (S2), $CDCl_3$, 23 ºC

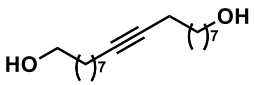

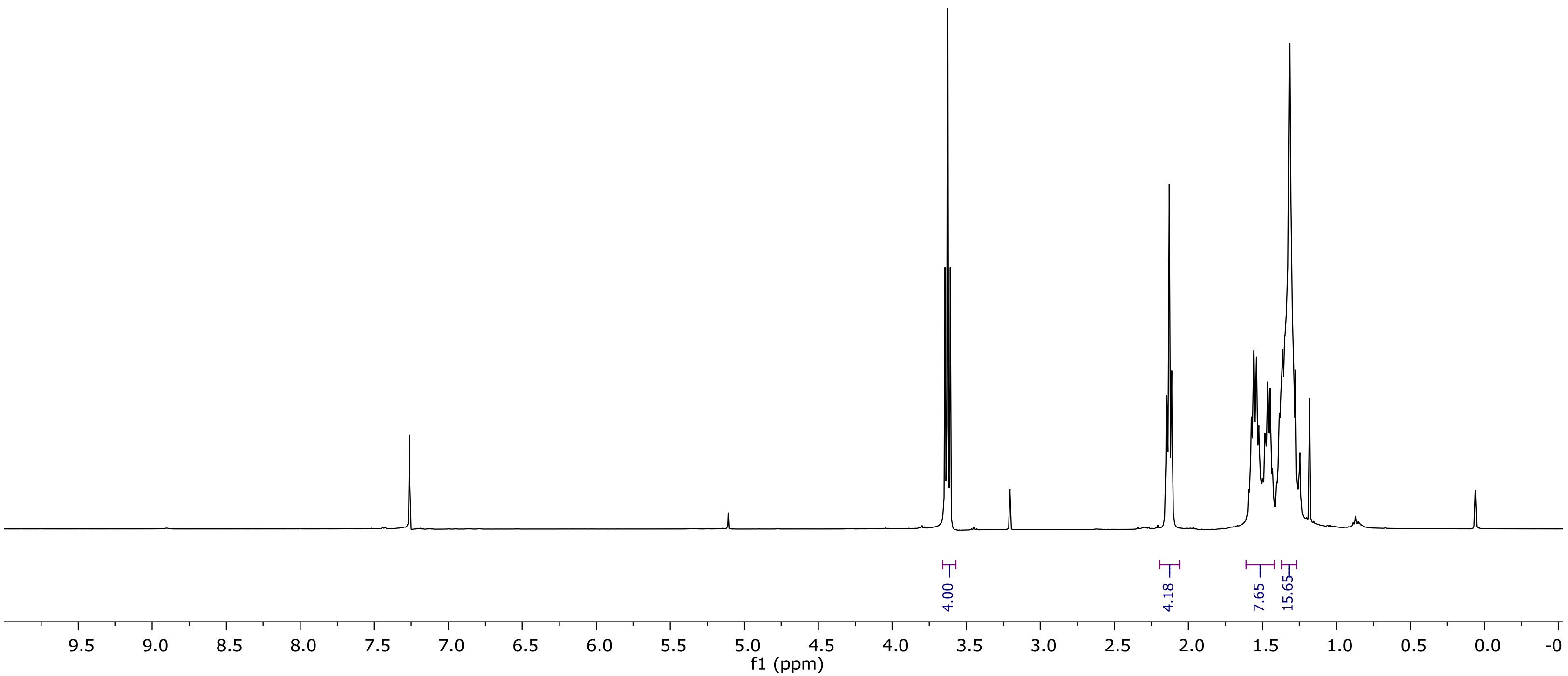

^{13}C NMR of Octadec-9-yne-1,18-diol (S2), $CDCl_3$, 23 °C

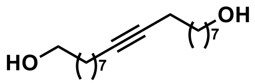

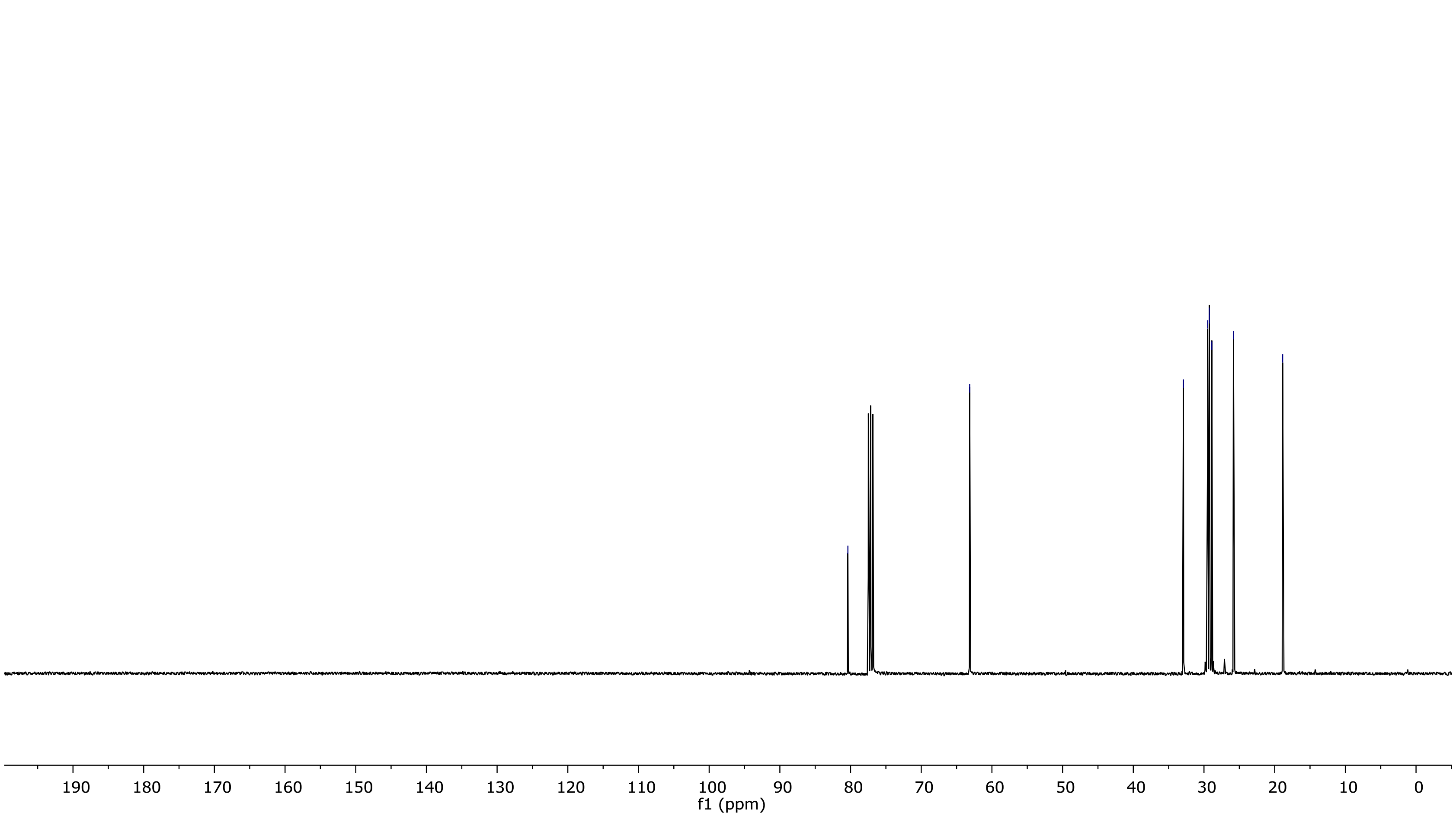

^{1}H NMR of 1,2-Bis(4-methoxyphenyl)ethyne (S3), $CDCl_3$, 23 ºC

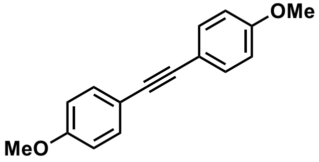

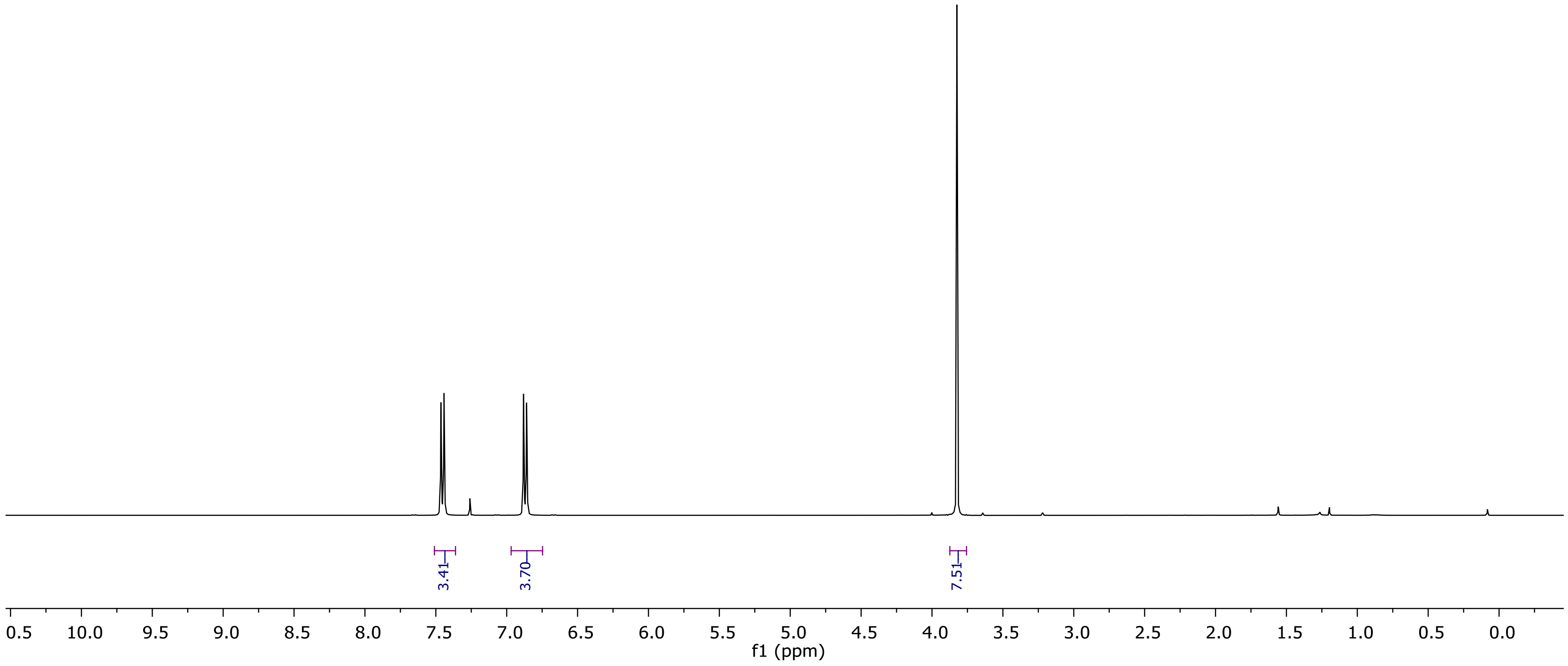

^{13}C NMR of 1,2-Bis(4-methoxyphenyl)ethyne (S3), $CDCl_3$, 23 °C

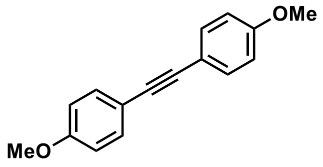

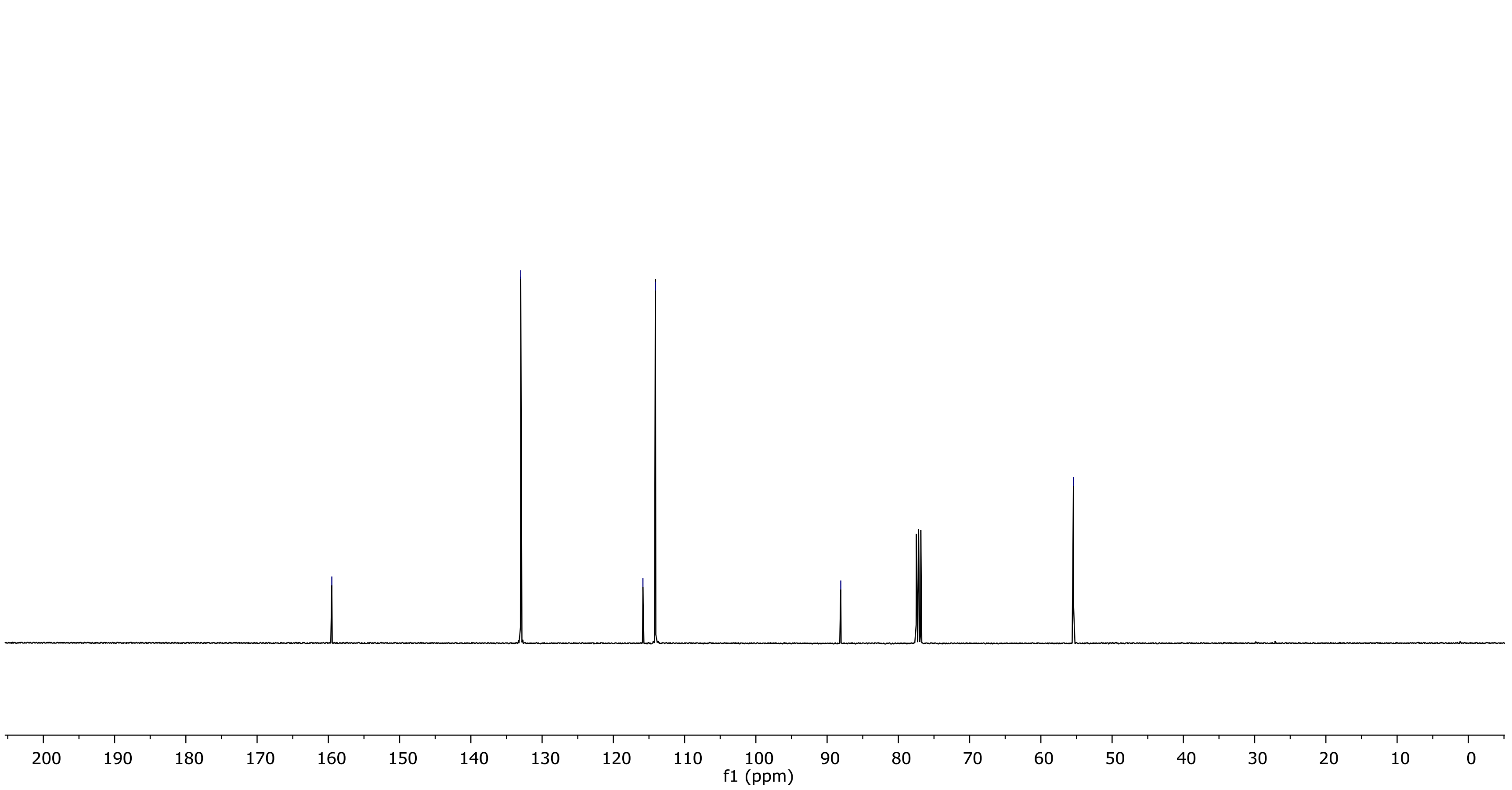

^{1}H NMR of 1,2-Bis(4-(trifluoromethyl)phenyl)ethyne (S4), $CDCl_3$, 23 °C

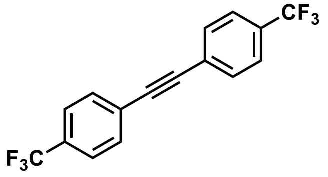

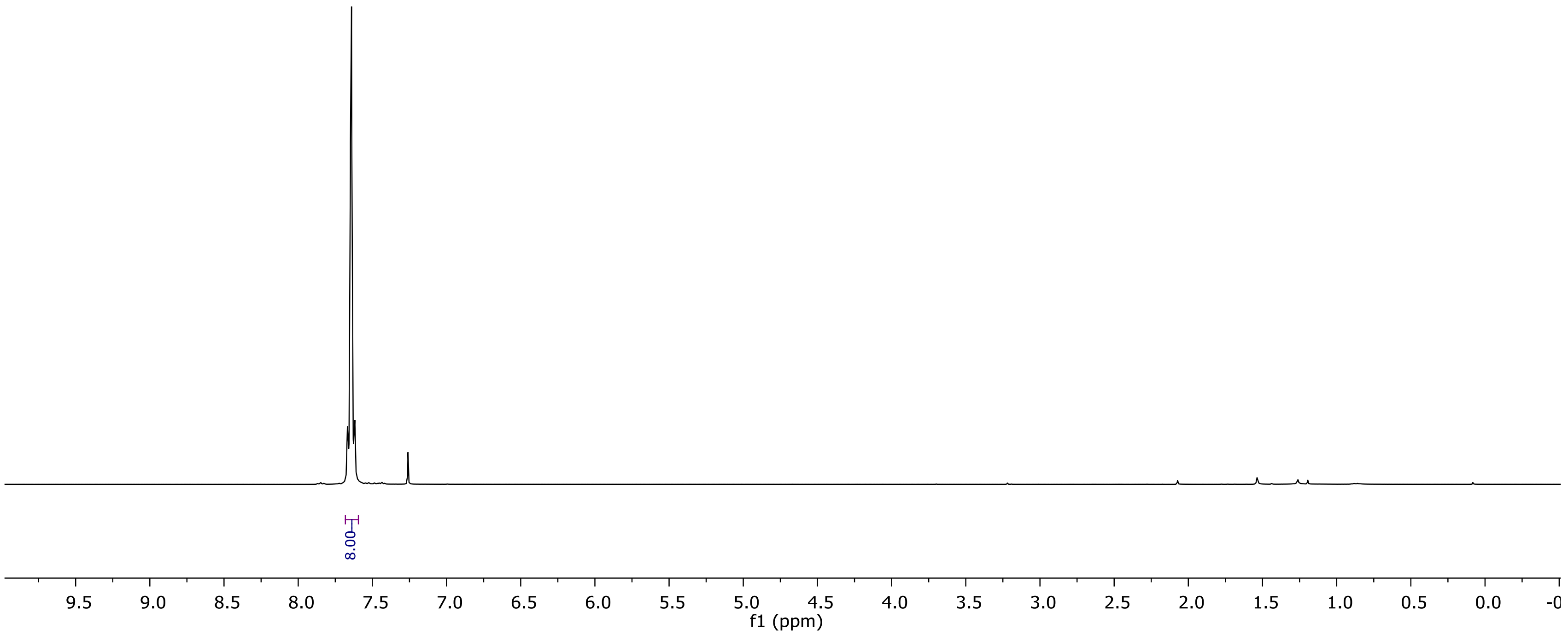

^{13}C NMR of 1,2-Bis(4-(trifluoromethyl)phenyl)ethyne (S4), $CDCl_3$, 23 ºC

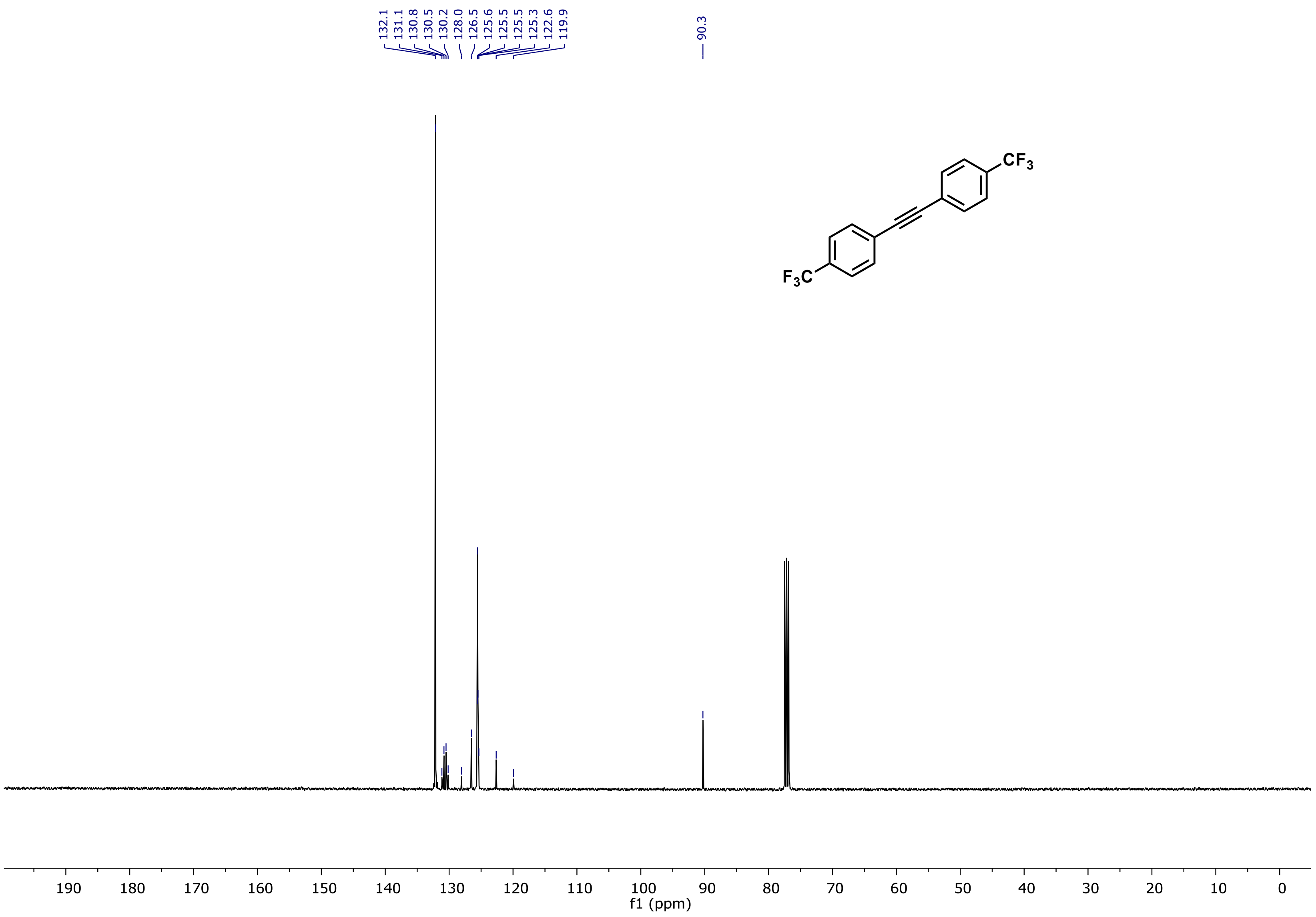

^{19}F NMR of 1,2-Bis(4-(trifluoromethyl)phenyl)ethyne (S4), $CDCl_3$, 23 ºC

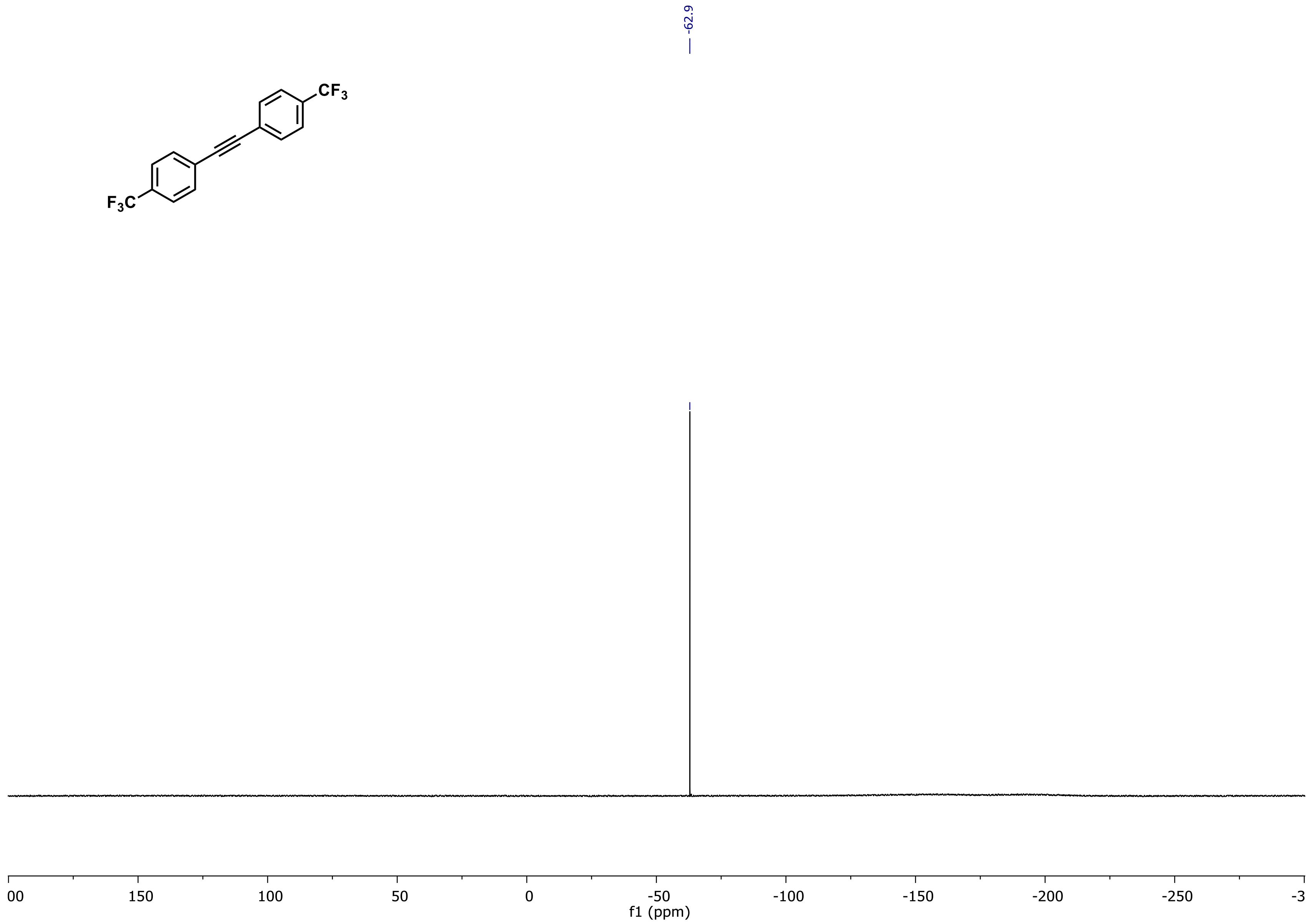

^{1}H NMR of 4,4'-(Ethyne-1,2-diyl)dibenzonitrile (S5), CDCl3, 23 ºC

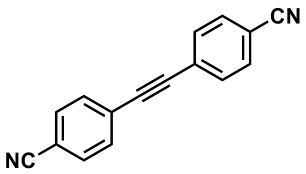

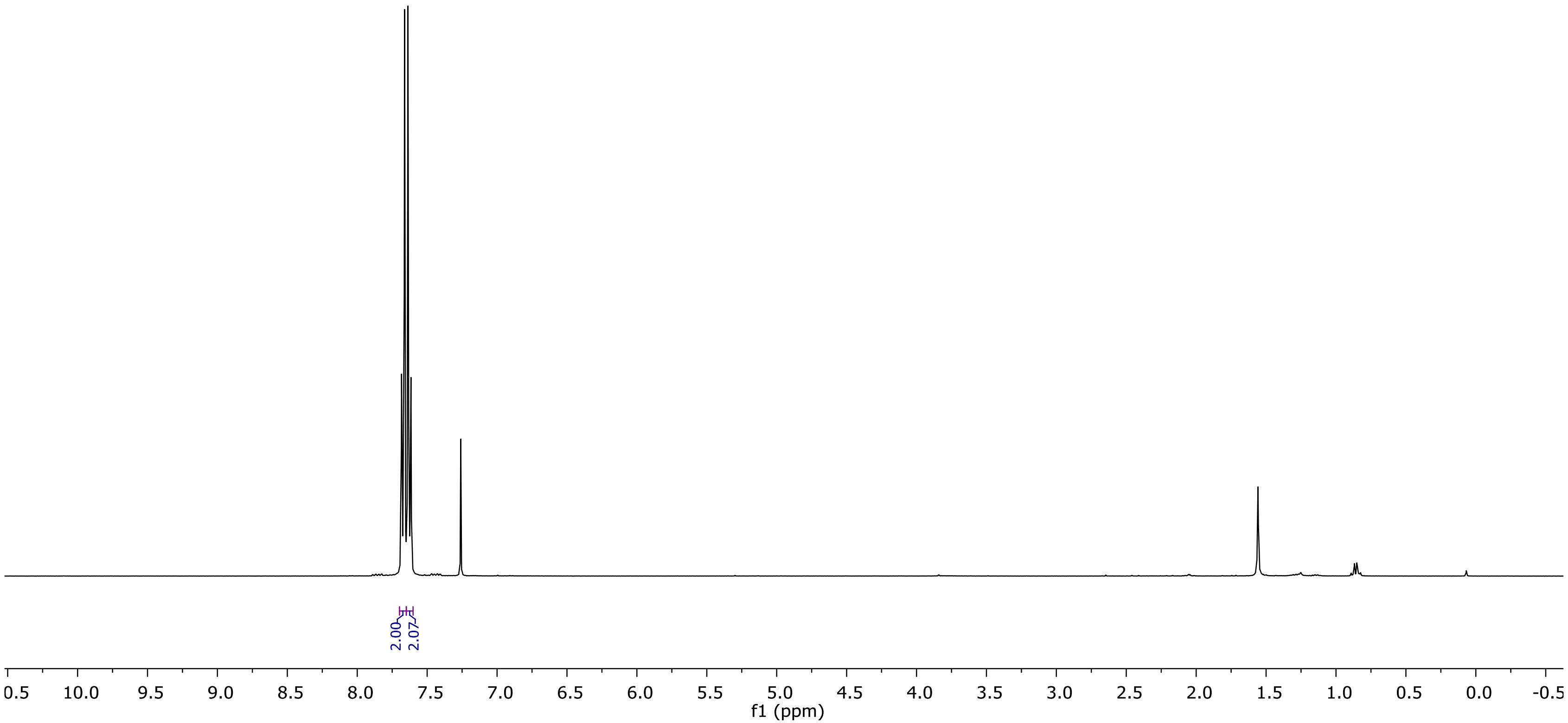

^{13}C NMR of 4,4'-(Ethyne-1,2-diyl)dibenzonitrile (S5), $CDCl_3$, 23 ºC

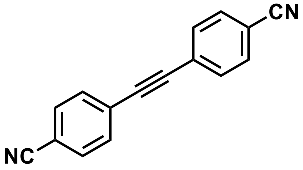

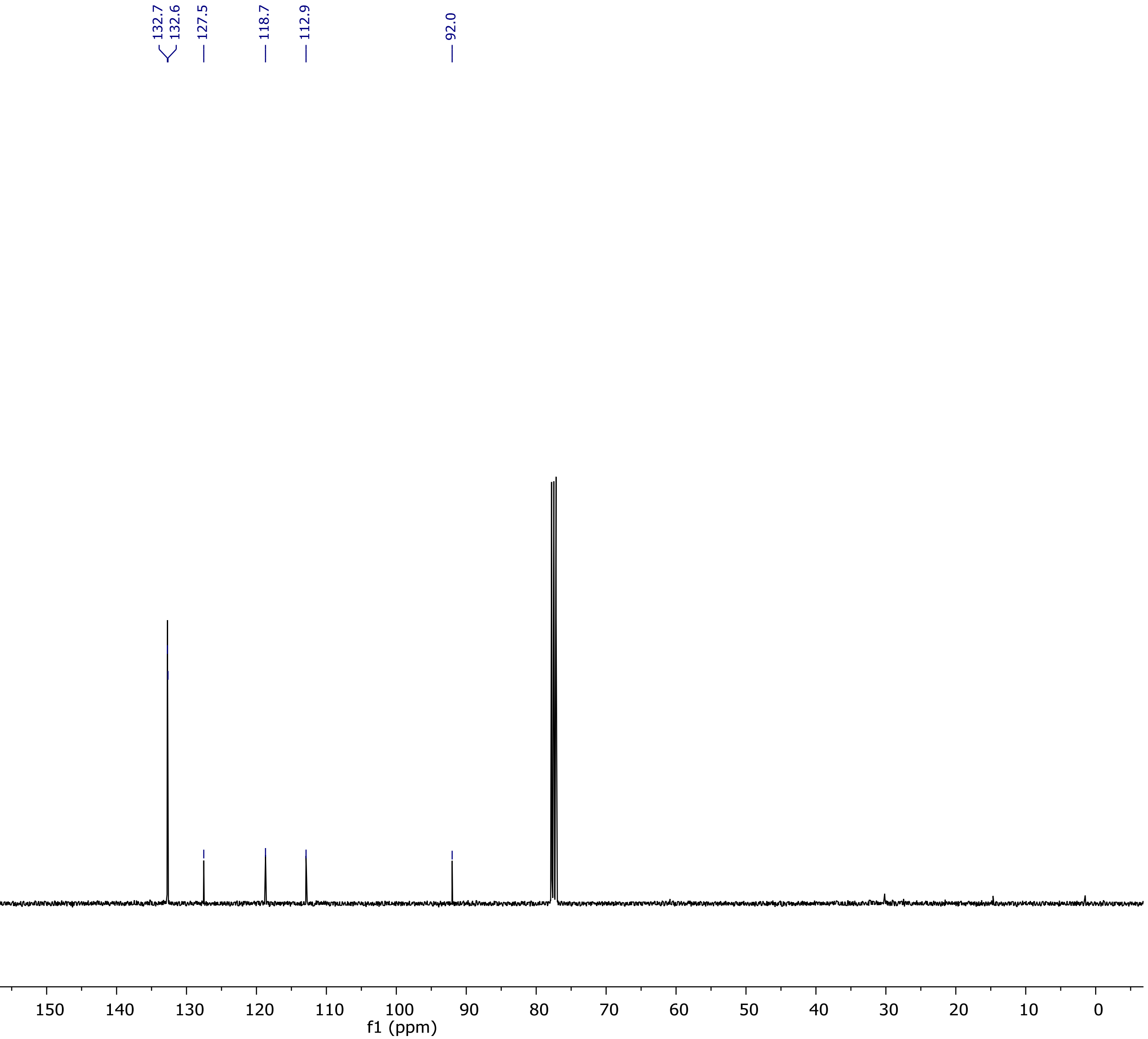

¹H NMR of 4,4'-(Ethyne-1,2-diyl)diphenol (S6), CD_3CN, 23 ºC

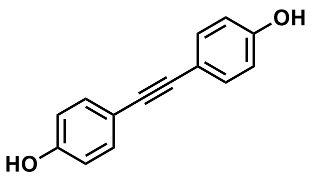

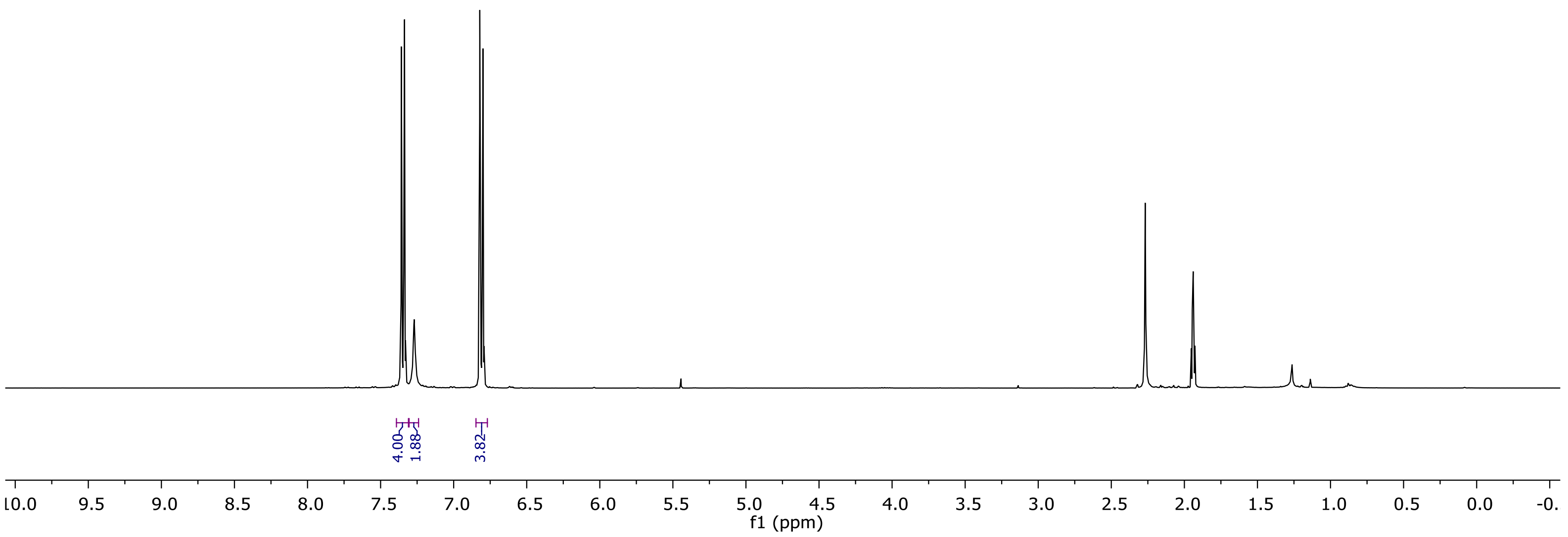

^{13}C NMR of 4,4'-(Ethyne-1,2-diyl)diphenol (S6), CD_3CN, 23 ºC

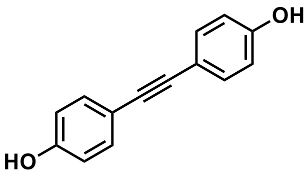

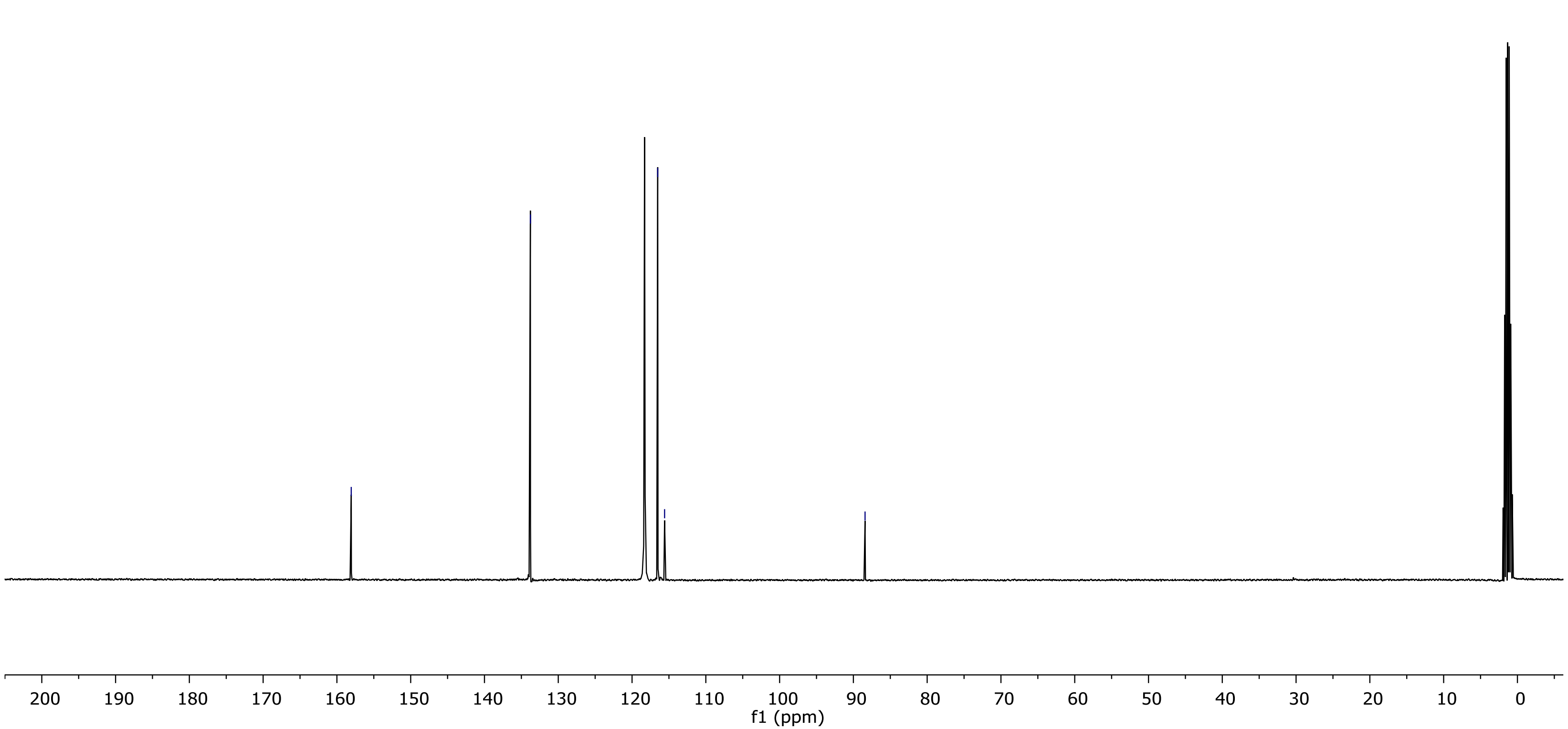

^{1}H NMR of Dimethyl 2,7-diacetamidooct-4-ynedioate (S7), $CDCl_3$, 23 ºC

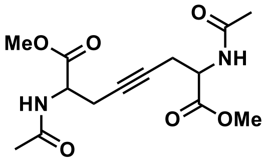

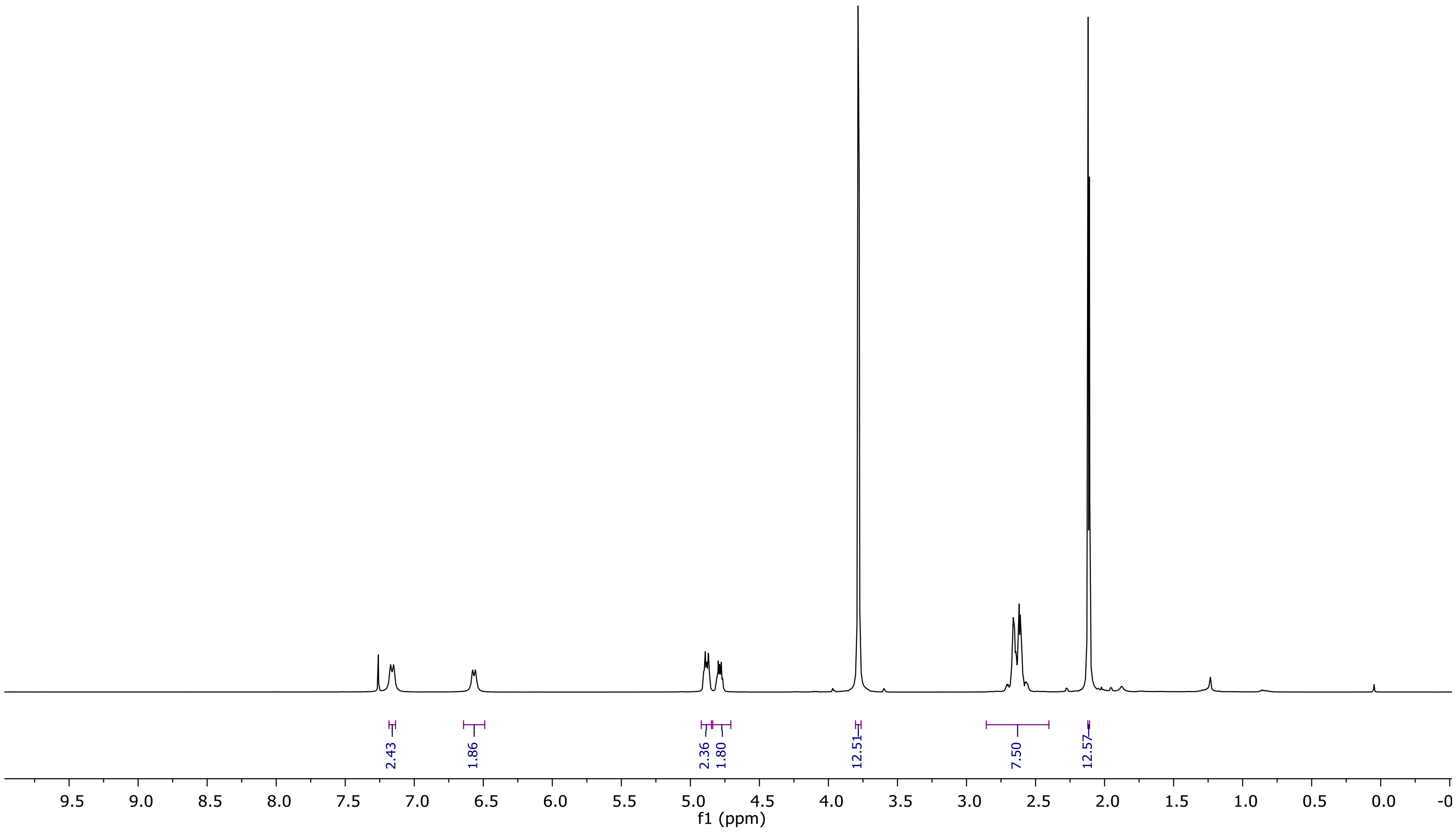

^{13}**C NMR of Dimethyl 2,7-diacetamidooct-4-ynedioate (S7),** $CDCl_3$, 23 ºC

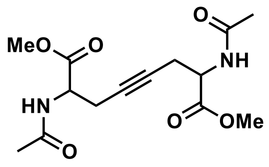

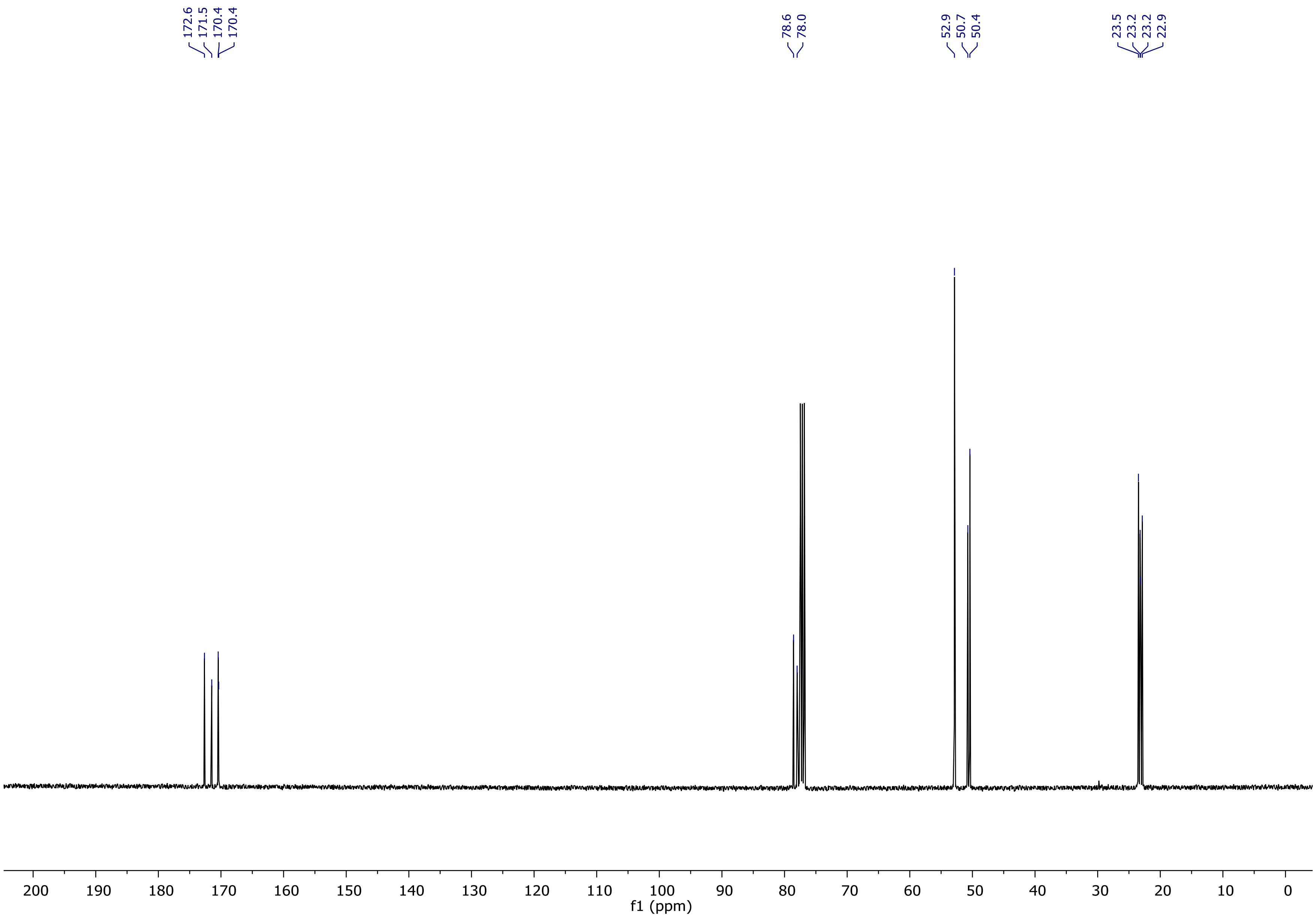

^{1}H NMR of *O*,*O*'-(Dodec-6-yne-1,12-diyl) dimethyl dimalonate *(*S8*),* CDCl$_3$, 23 ºC

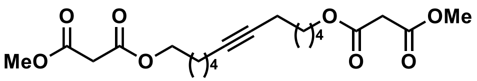
MeO
OMe
O
4

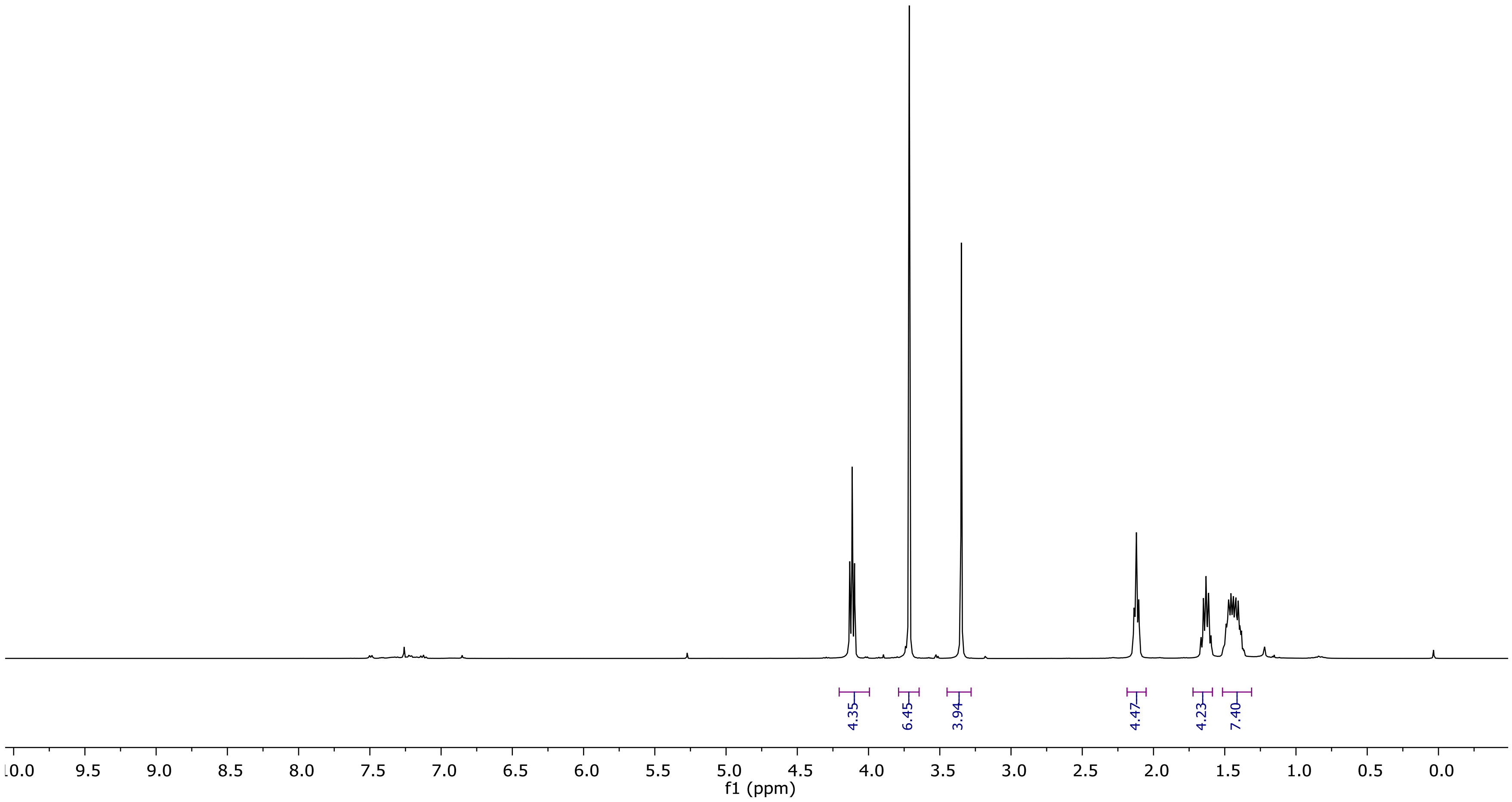
4.35
6.45
3.94
4.47
4.23
7.40
10.0
9.5
9.0
8.5
8.0
7.5
7.0
6.5
6.0
5.5
5.0
4.5
4.0
3.5
3.0
2.5
2.0
1.5
1.0
0.5
0.0
f1 (ppm)

^{13}C NMR of *O*,*O*'-(Dodec-6-yne-1,12-diyl) dimethyl dimalonate *(S8)*, CDCl$_3$, 23 ºC

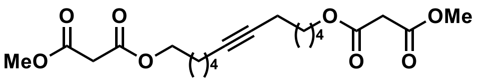

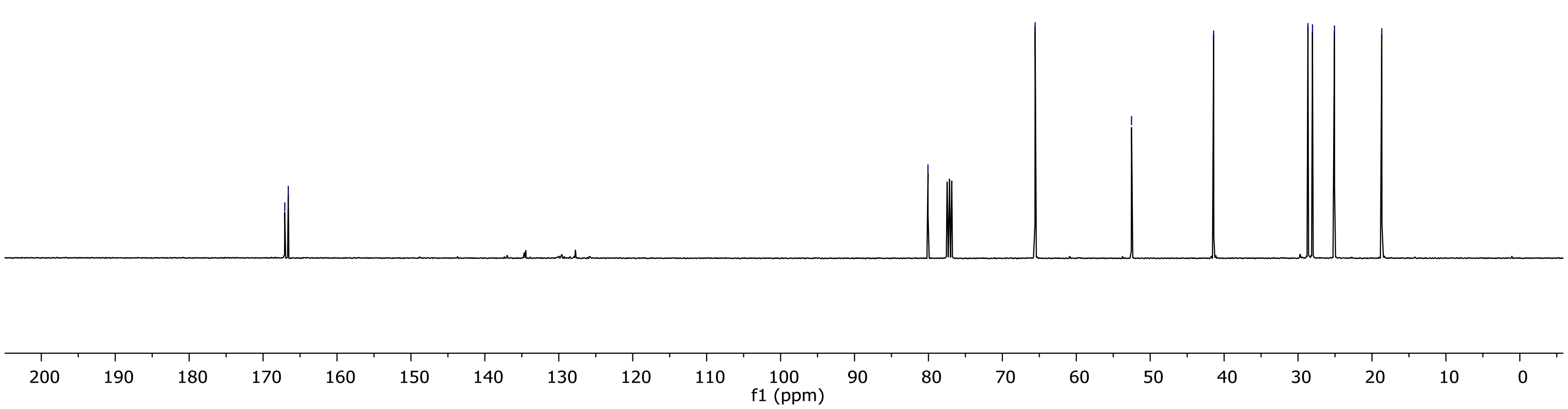

^{1}H NMR of N^{1},N^{8}-dimethoxy-N^{1},N^{8}-dimethyloct-4-ynediamide (S9), $CDCl_3$, 23 ºC

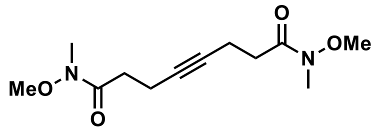

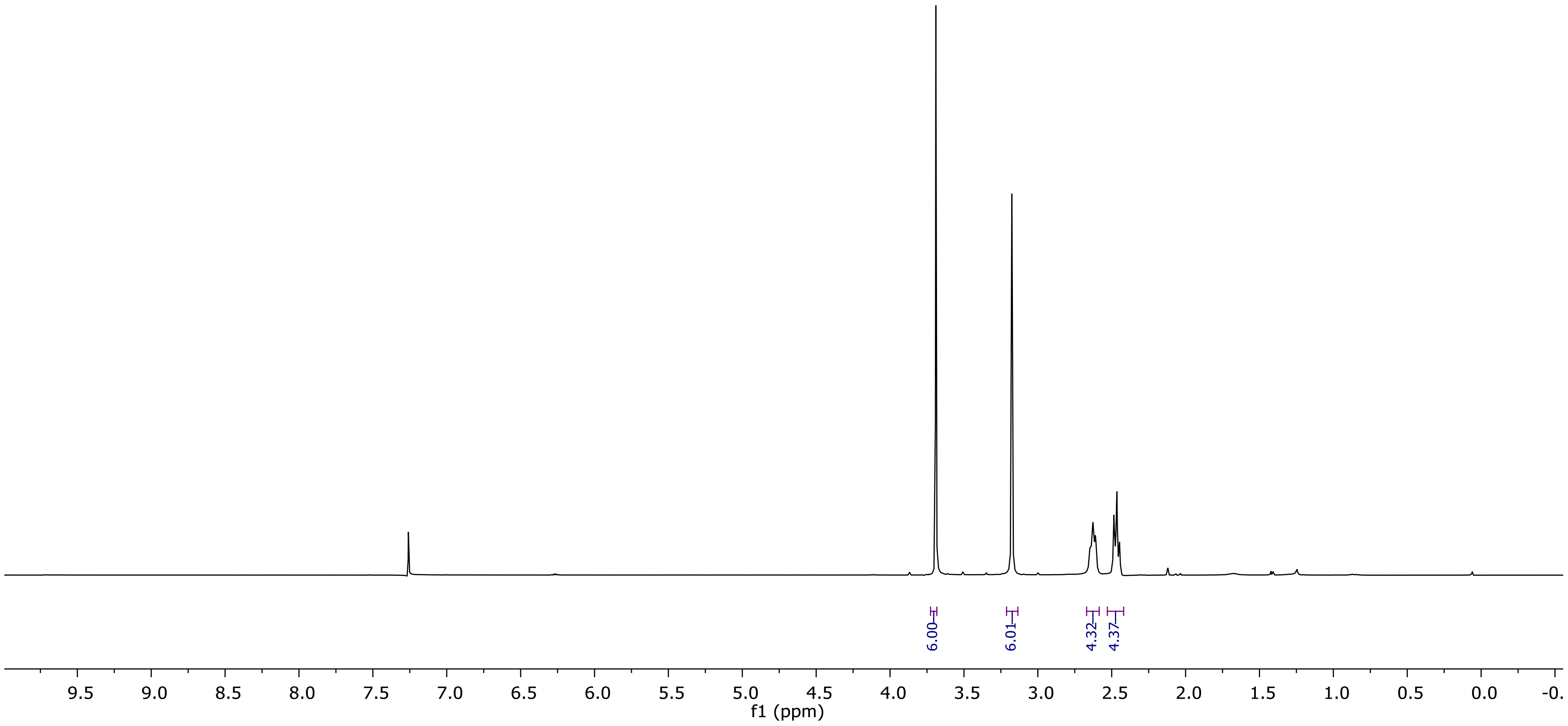

^{13}C NMR of *N*[1],*N*[8]-dimethoxy-*N*[1],*N*[8]-dimethyloct-4-ynediamide (S9), $CDCl_3$, 23 ºC

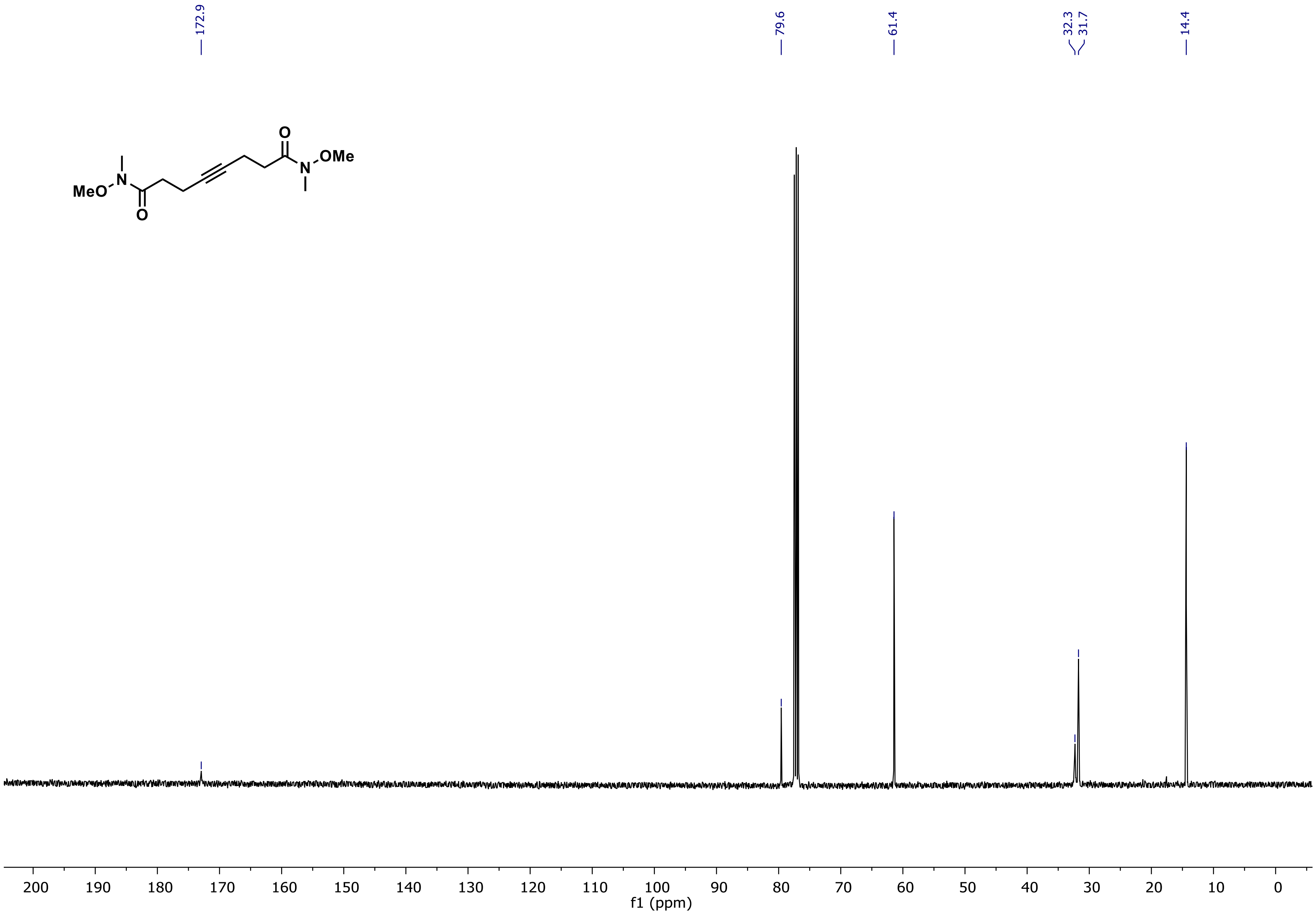

^{1}H NMR of 2,2'-(Octadec-9-yne-1,18-diyl)bis(isoindoline-1,3-dione) (S10), $CDCl_3$, 23 ºC

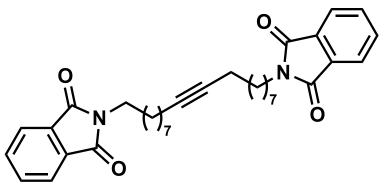

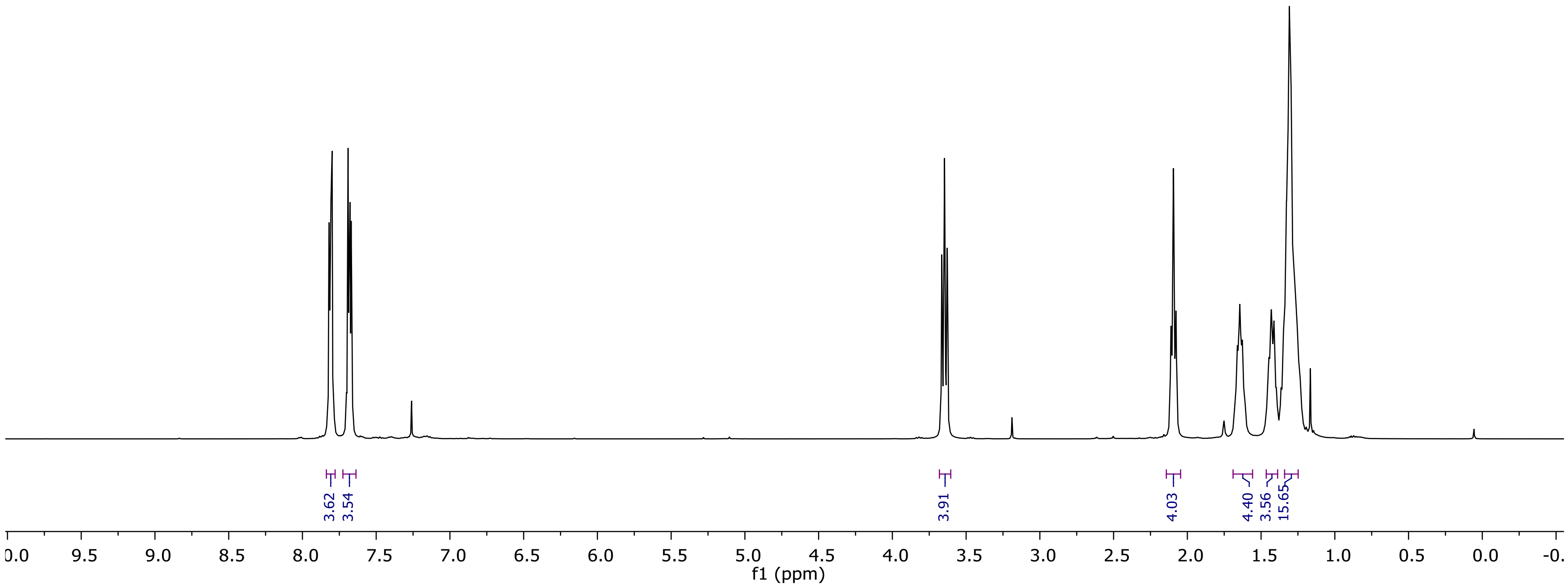

^{13}C NMR of 2,2'-(Octadec-9-yne-1,18-diyl)bis(isoindoline-1,3-dione) (S10), $CDCl_3$, 23 ºC

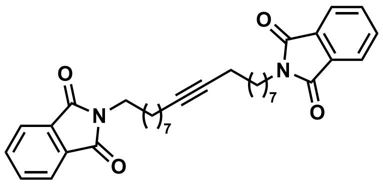

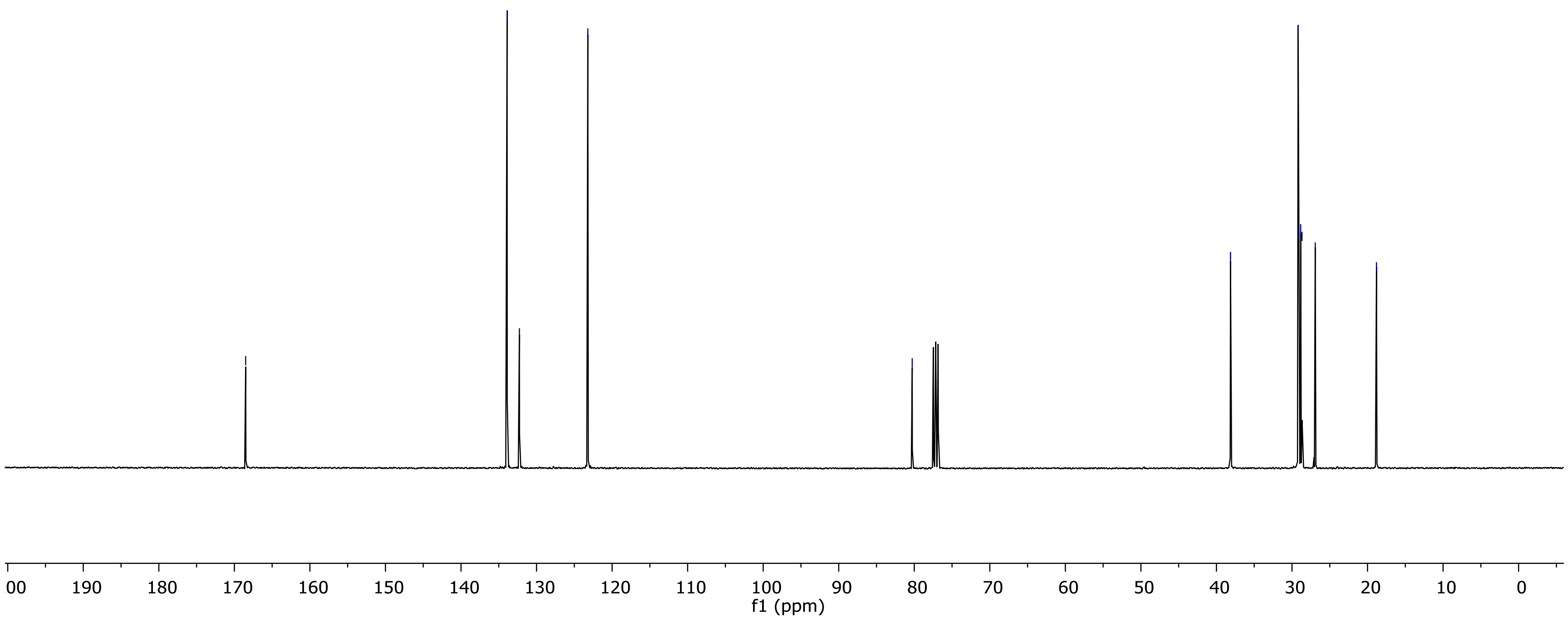

^{1}H NMR of *N*^{1},*N*^{12}-Dibutyldodec-6-yne-1,12-diamine (S11), $CDCl_3$, 23 ^{o}C

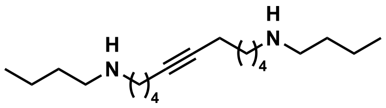

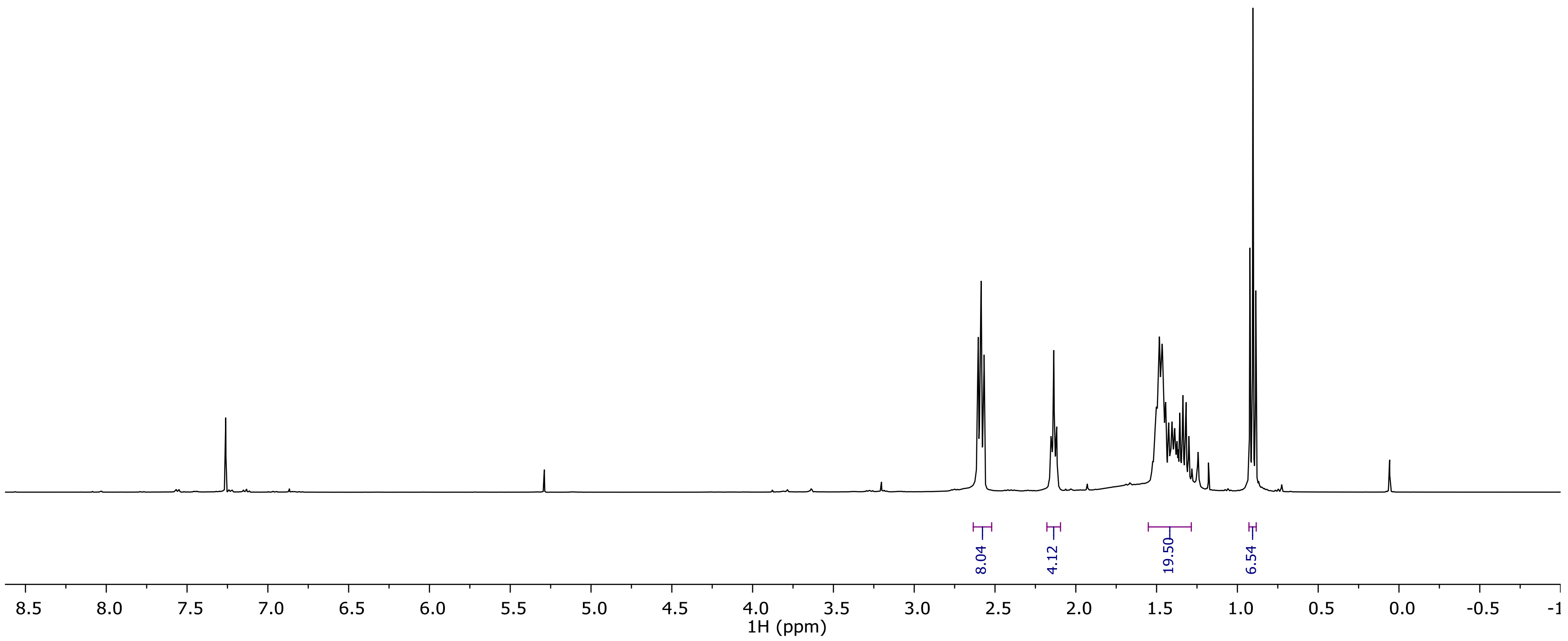

^{13}C NMR of *N*^{1},*N*^{12}-dibutyldodec-6-yne-1,12-diamine (S11), $CDCl_3$, 23 °C

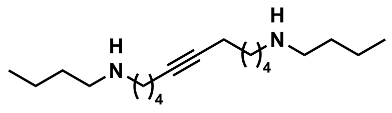

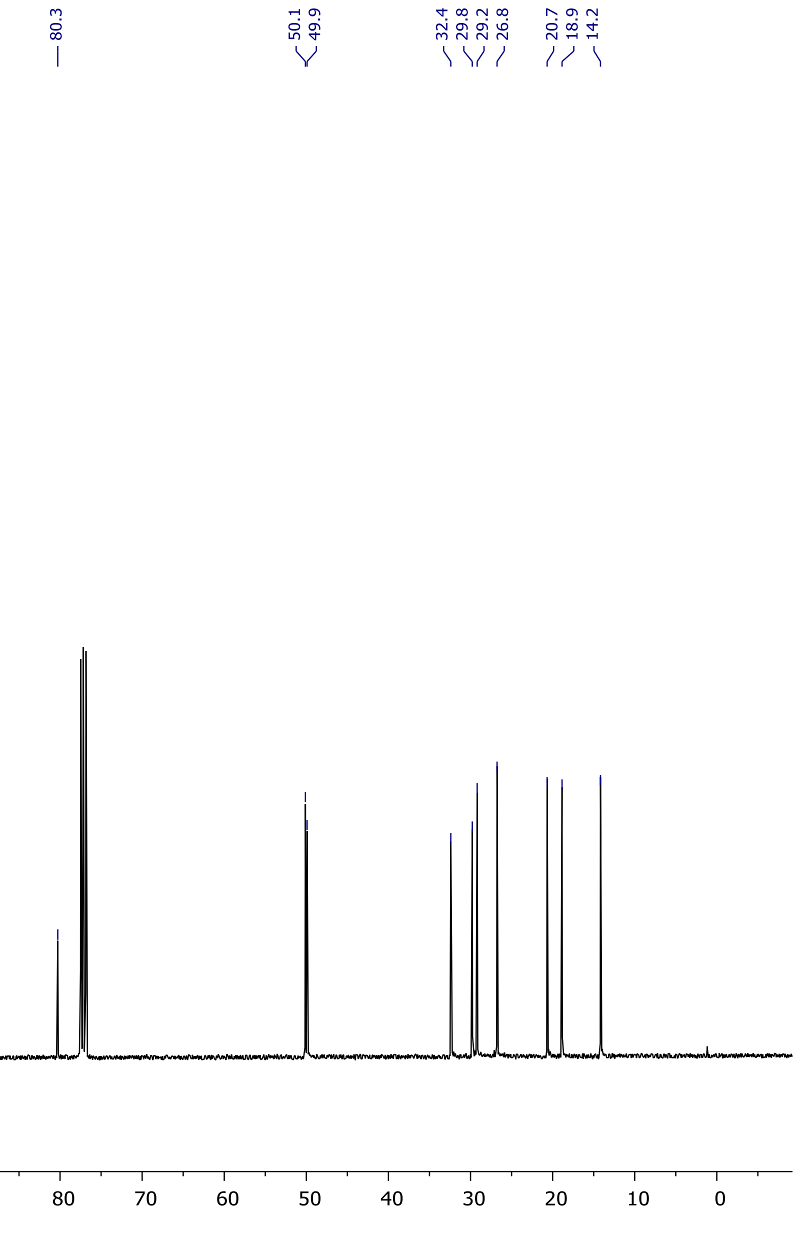

^{1}H NMR of 1,12-Di(piperidin-1-yl)dodec-6-yne (S12), $CDCl_3$, 23 ºC

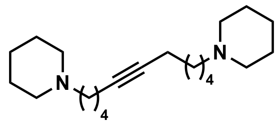

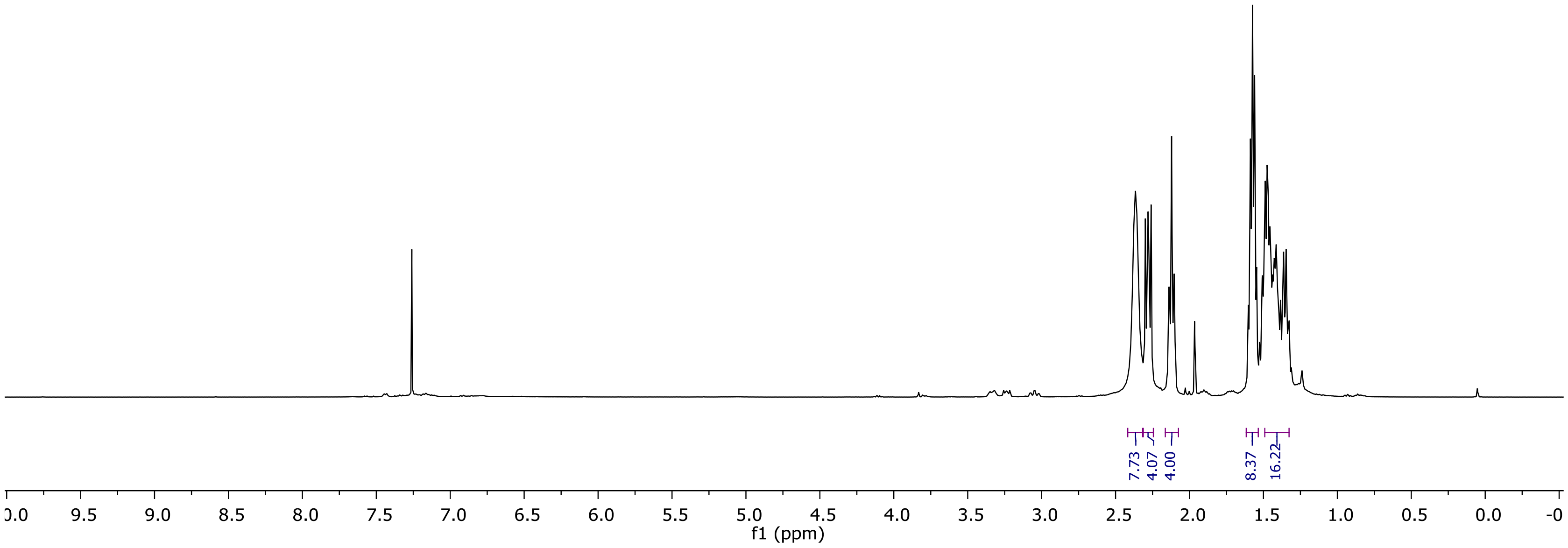

^{13}C NMR of 1,12-Di(piperidin-1-yl)dodec-6-yne (S12), $CDCl_3$, 23 ºC

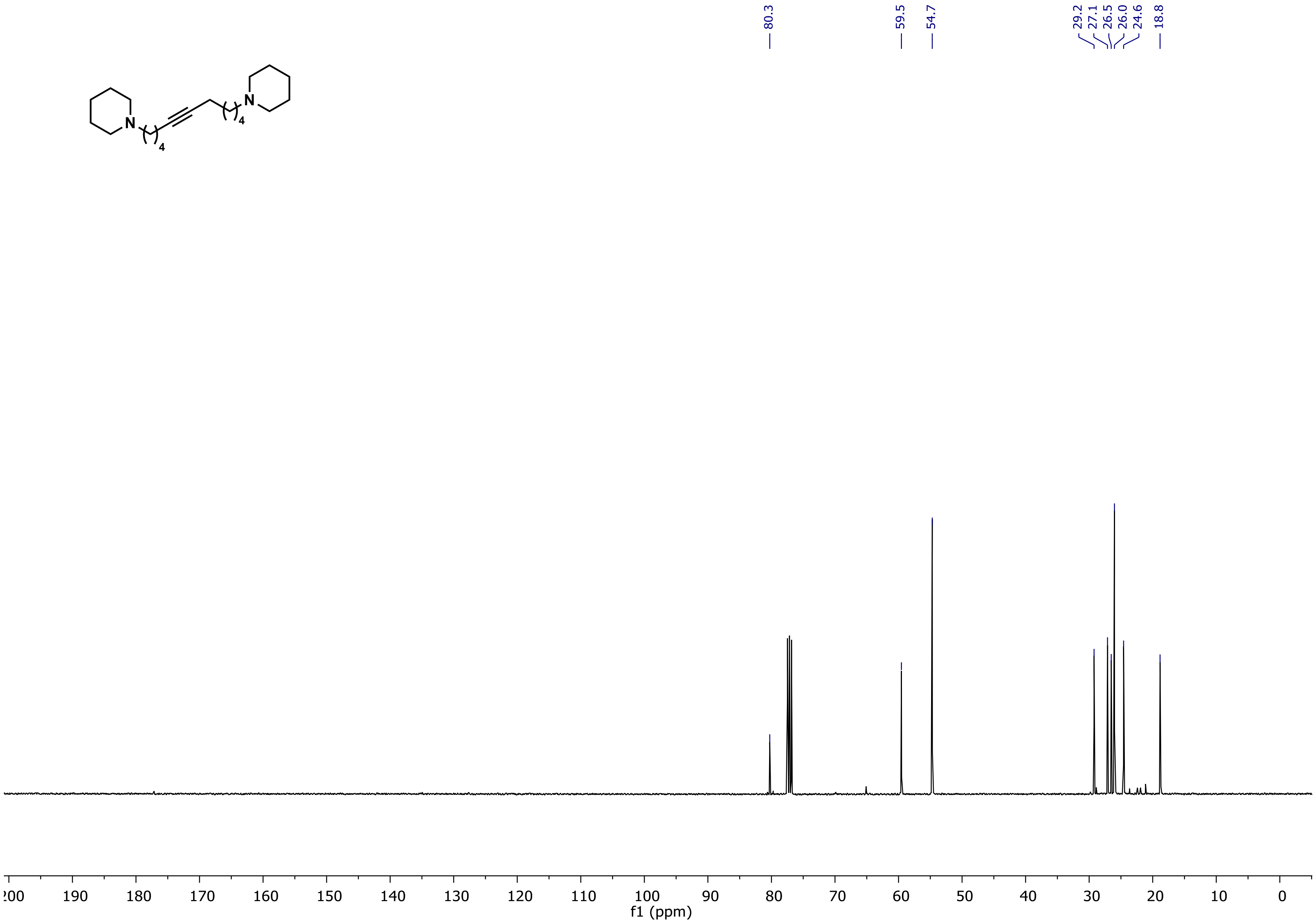

^{1}H NMR of Hex-3-yne-1,6-diyl bis(4-methylbenzenesulfonate) (S13), $CDCl_3$, 23 ºC

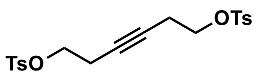

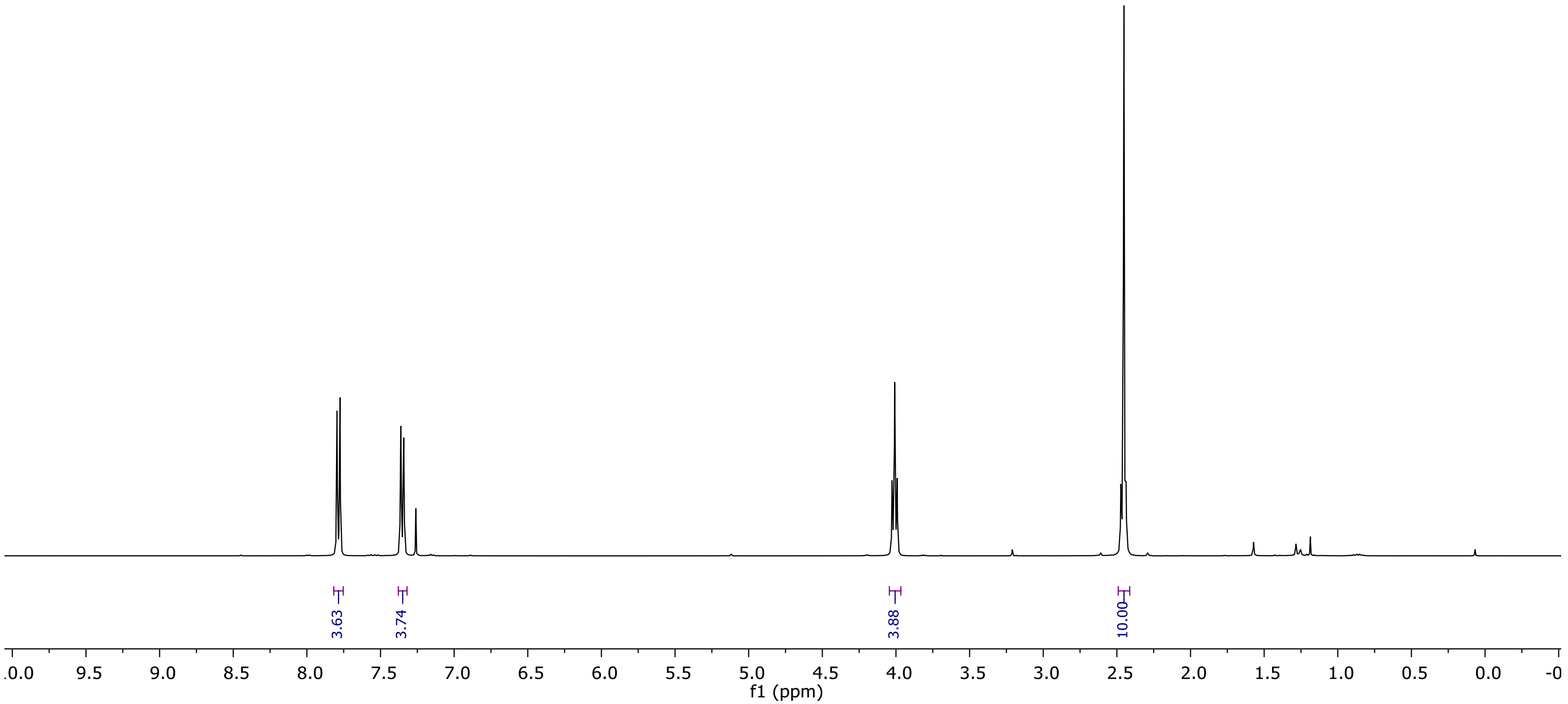

^{13}C NMR of Hex-3-yne-1,6-diyl bis(4-methylbenzenesulfonate) (S13), $CDCl_3$, 23 ºC

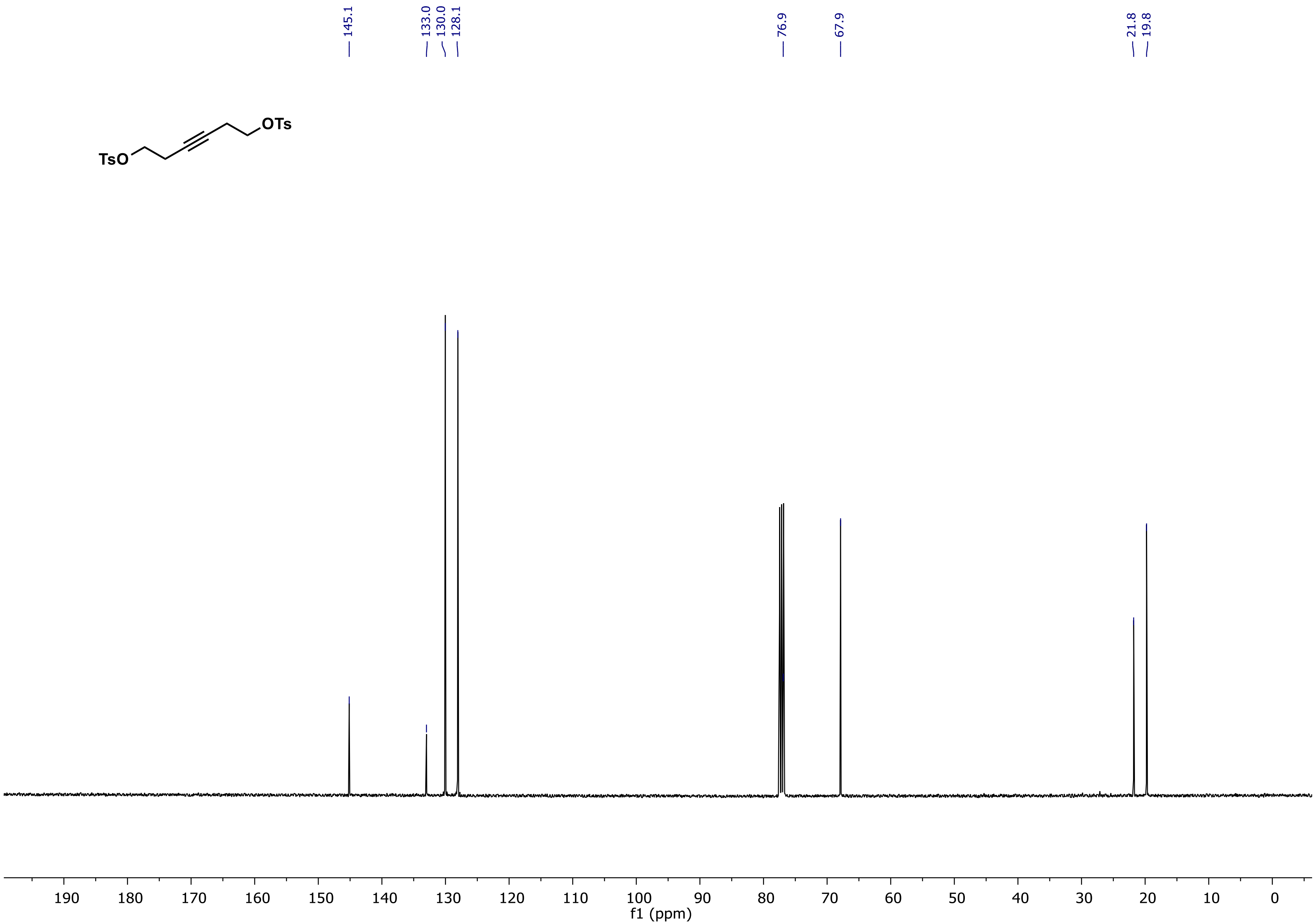

^{1}H NMR of 1,8-Dioxacyclotetradec-11-yne-2,7-dione (S14), $CDCl_3$, 23 ºC

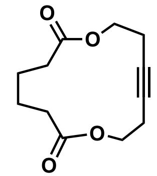

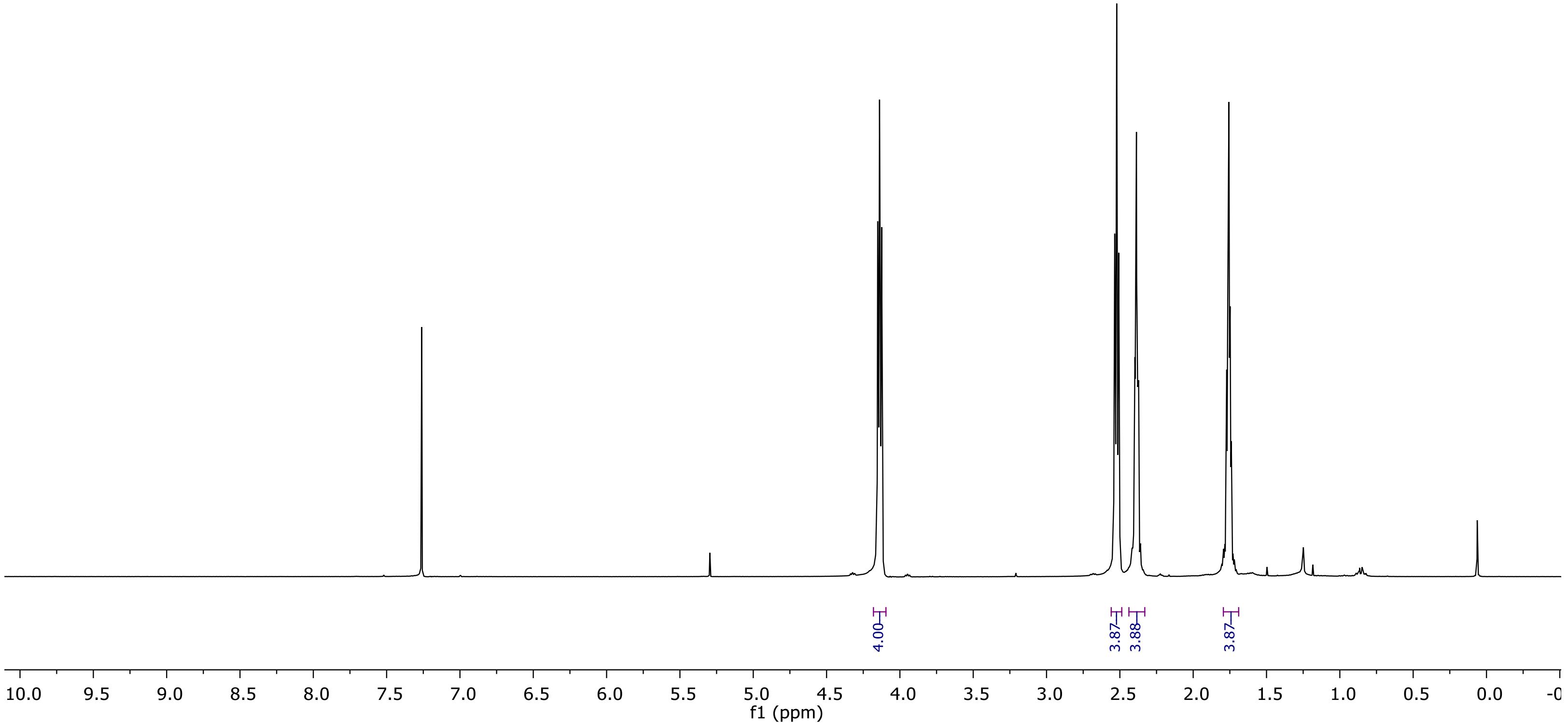

^{13}C NMR of 1,8-Dioxacyclotetradec-11-yne-2,7-dione (S14), $CDCl_3$, 23 ºC

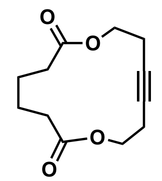

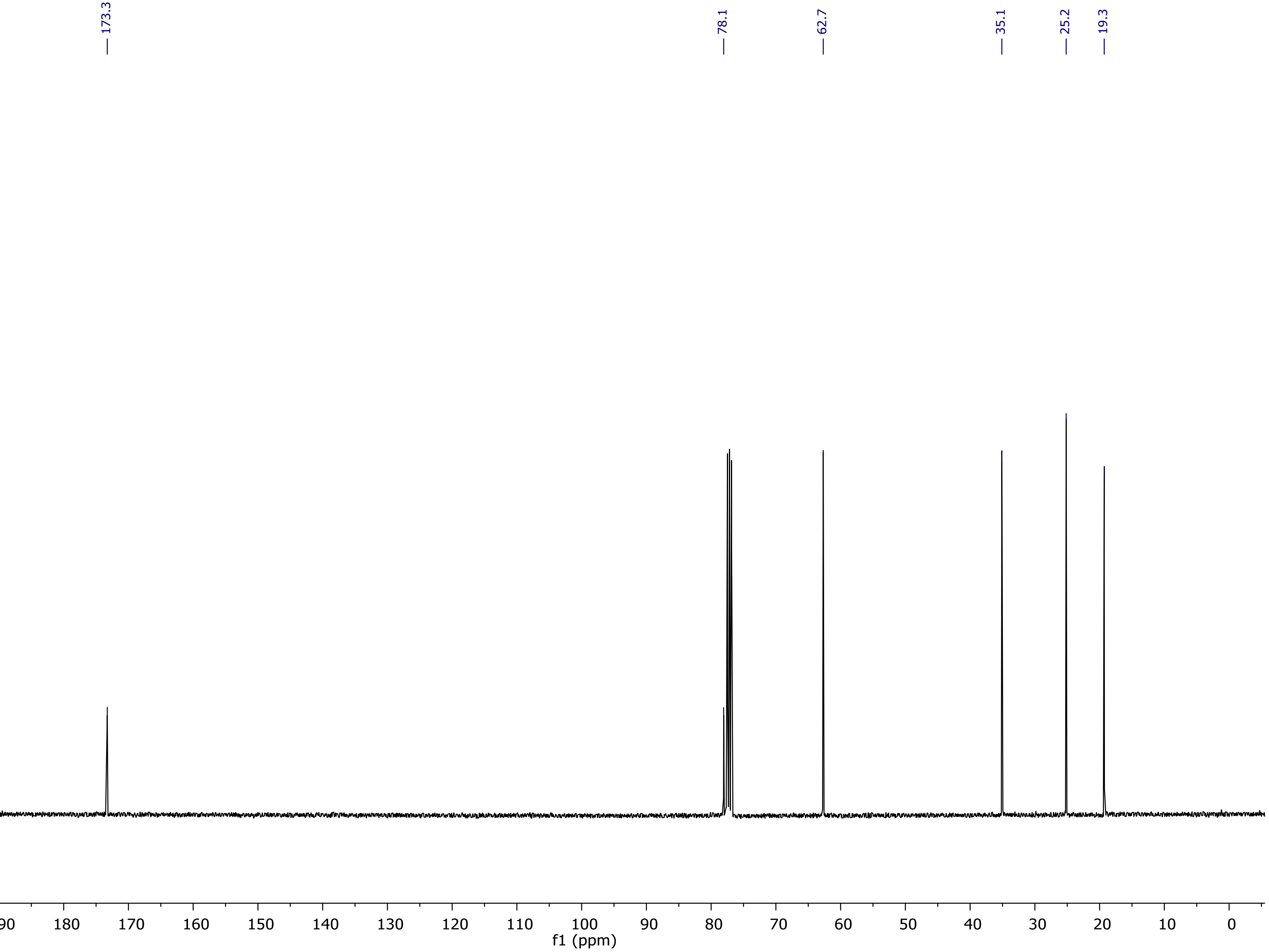

^{1}H NMR of 1,6-Dioxacyclododec-9-yne-2,5-dione (S15), $CDCl_3$, 23 ºC

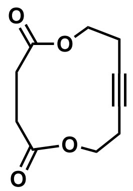

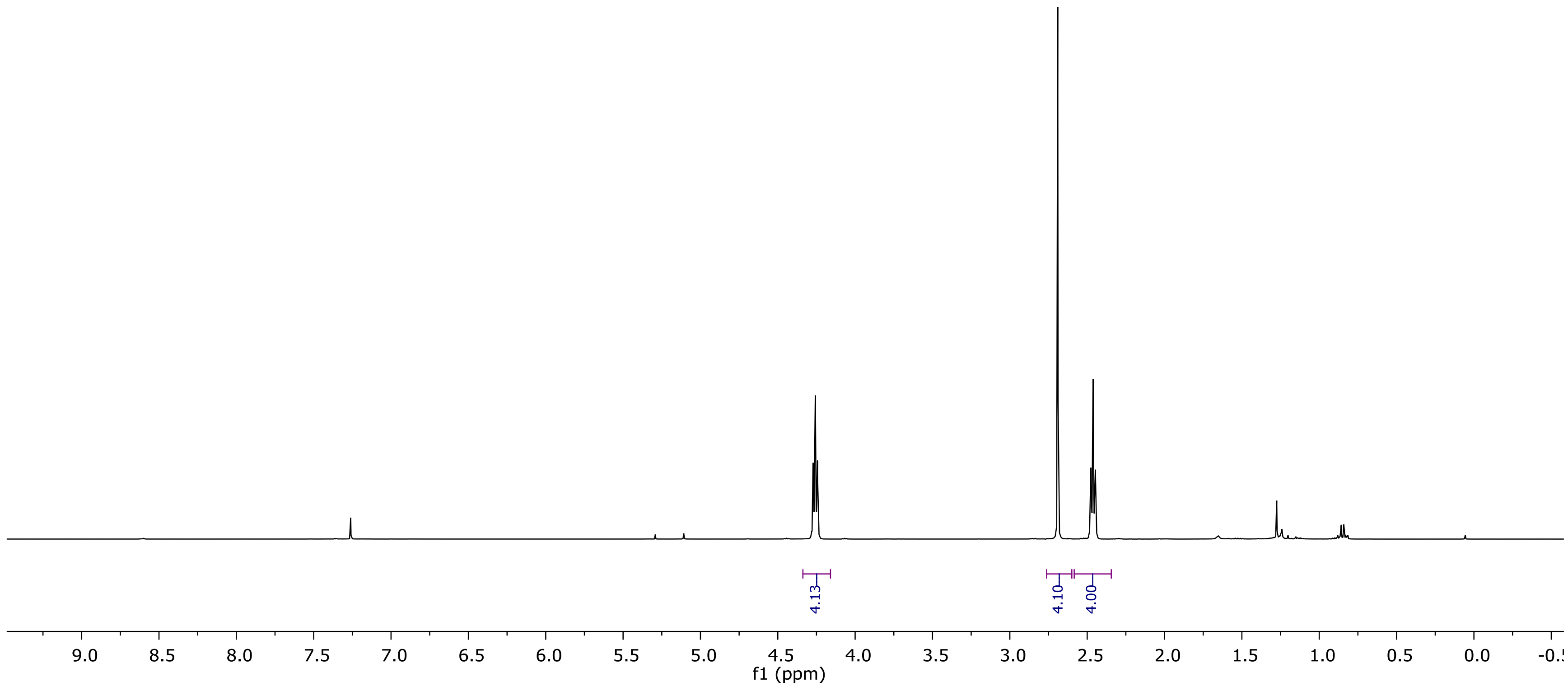

^{13}C NMR of 1,6-Dioxacyclododec-9-yne-2,5-dione (S15), $CDCl_3$, 23 ºC

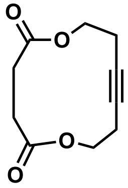

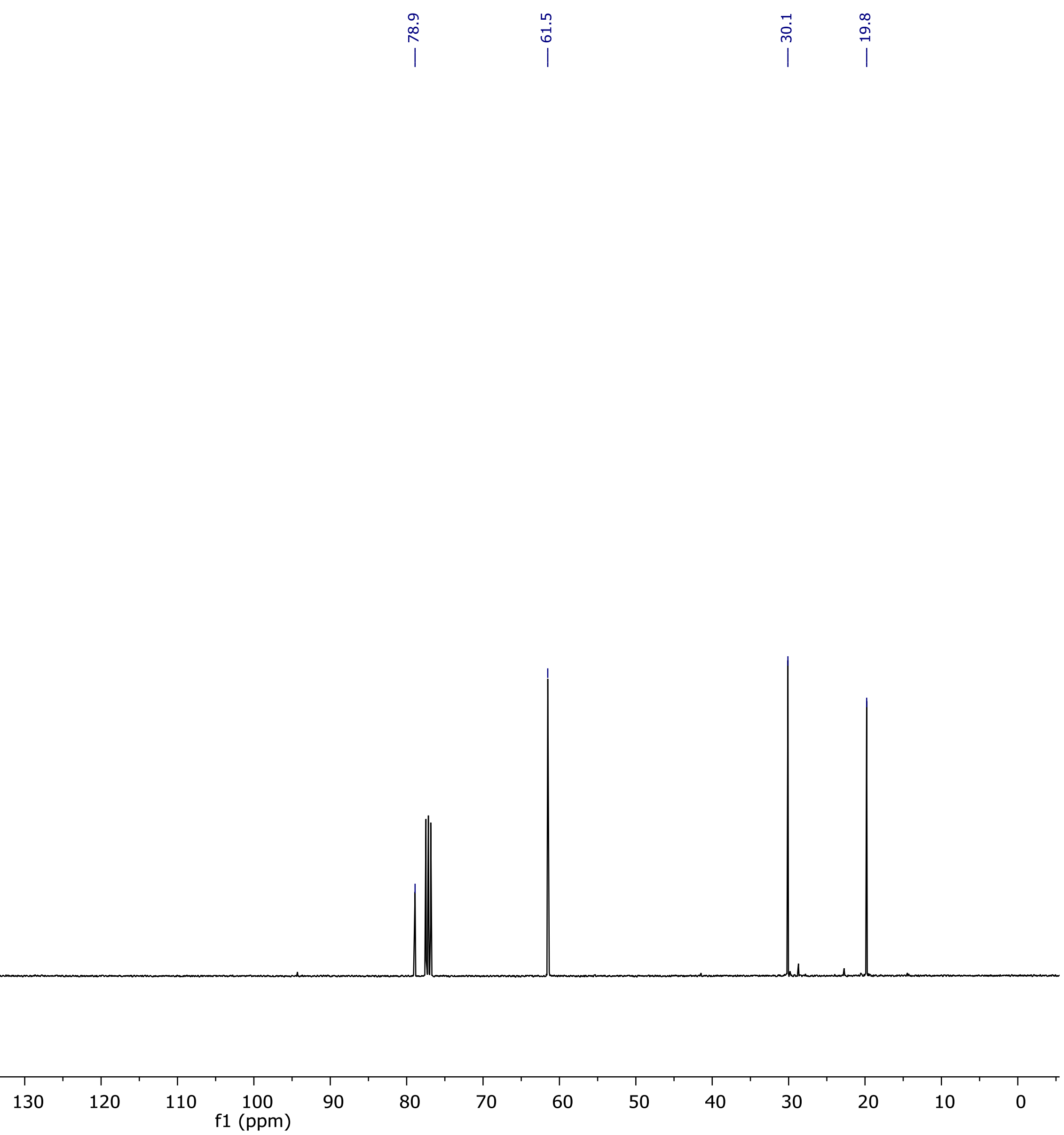

^{1}H NMR of Compound S16, $CDCl_3$, 23 ºC

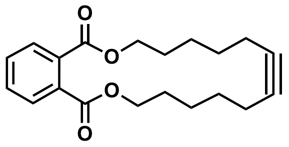

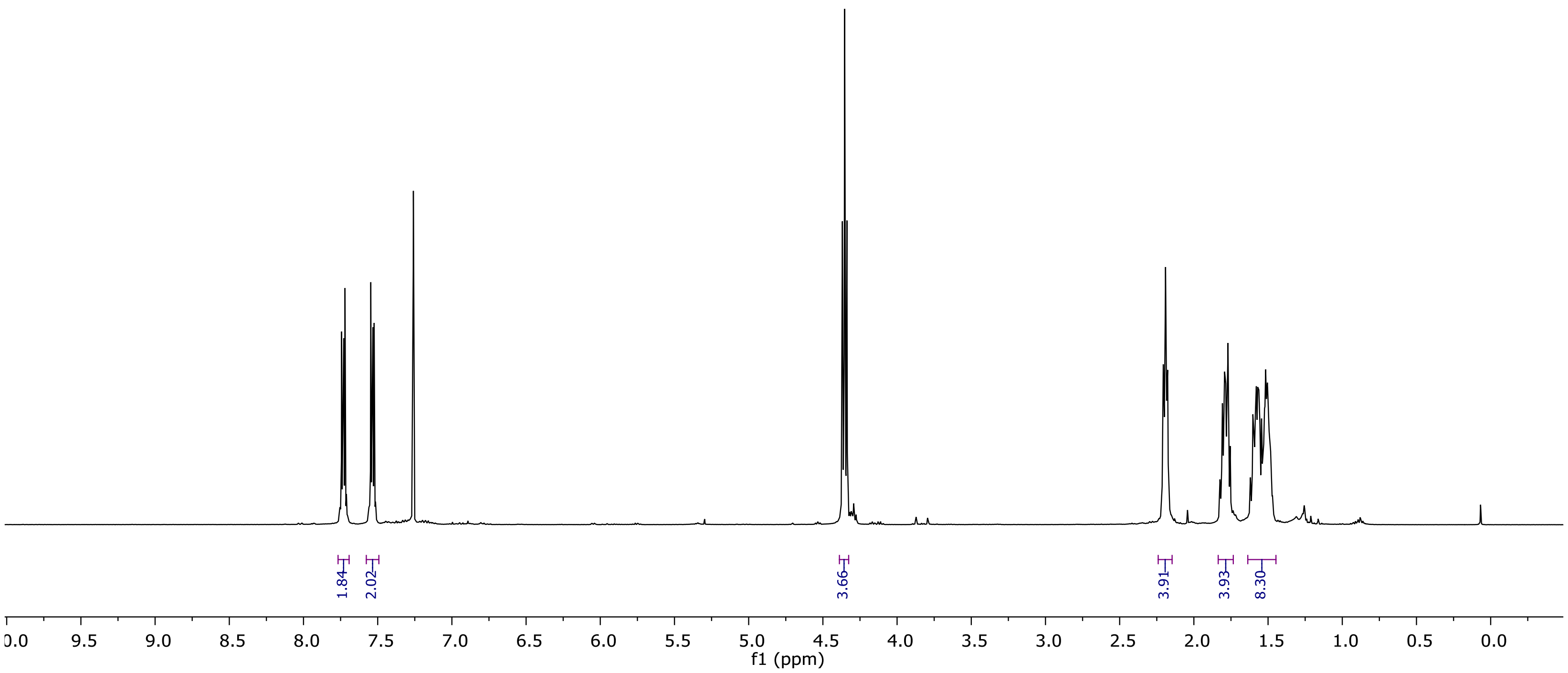

^{13}C NMR of Compound S16, $CDCl_3$, 23 ^{o}C

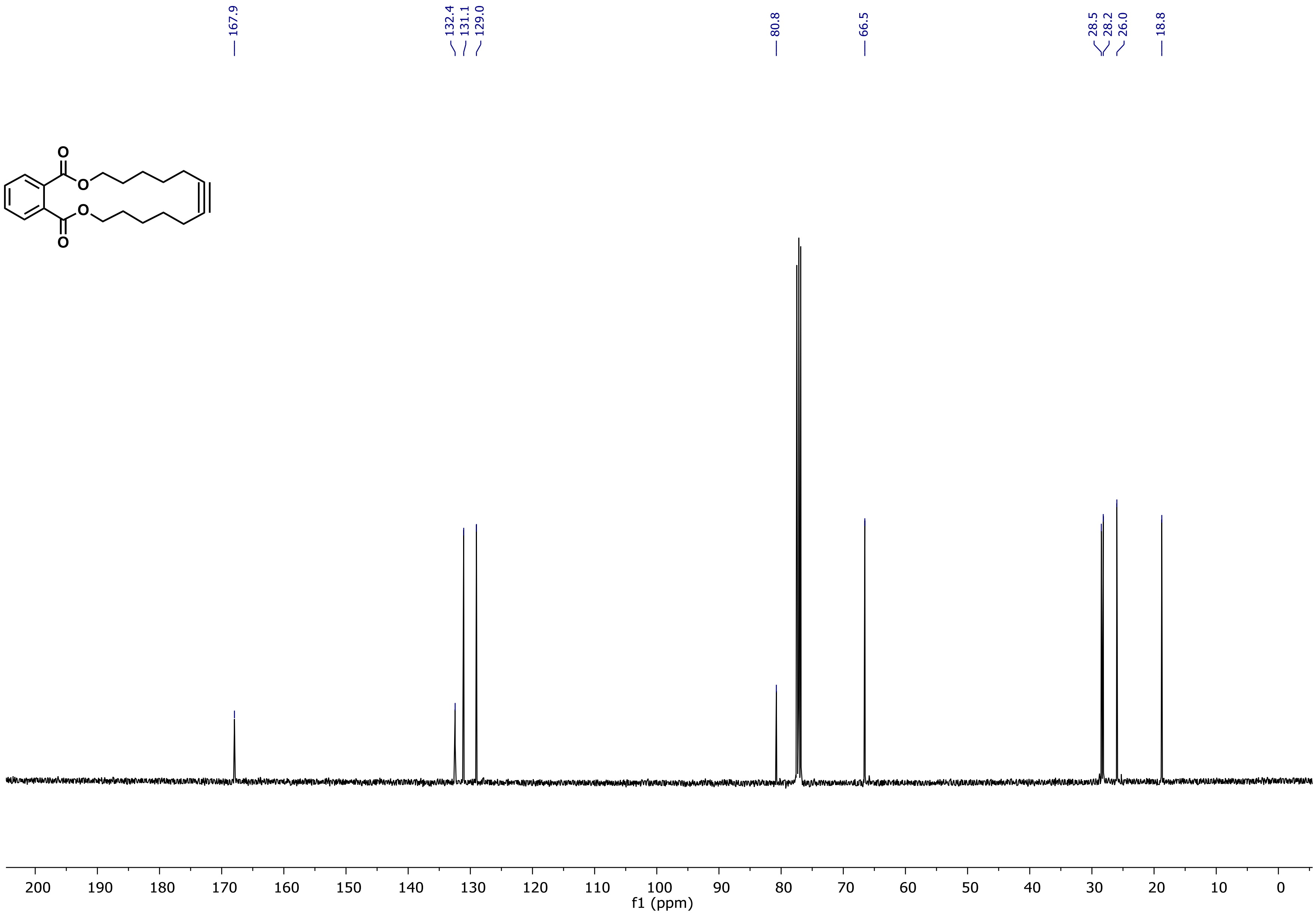

^{1}H NMR of compound S17, $CDCl_3$, 23 ^{o}C

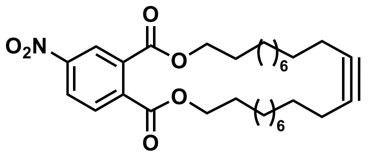

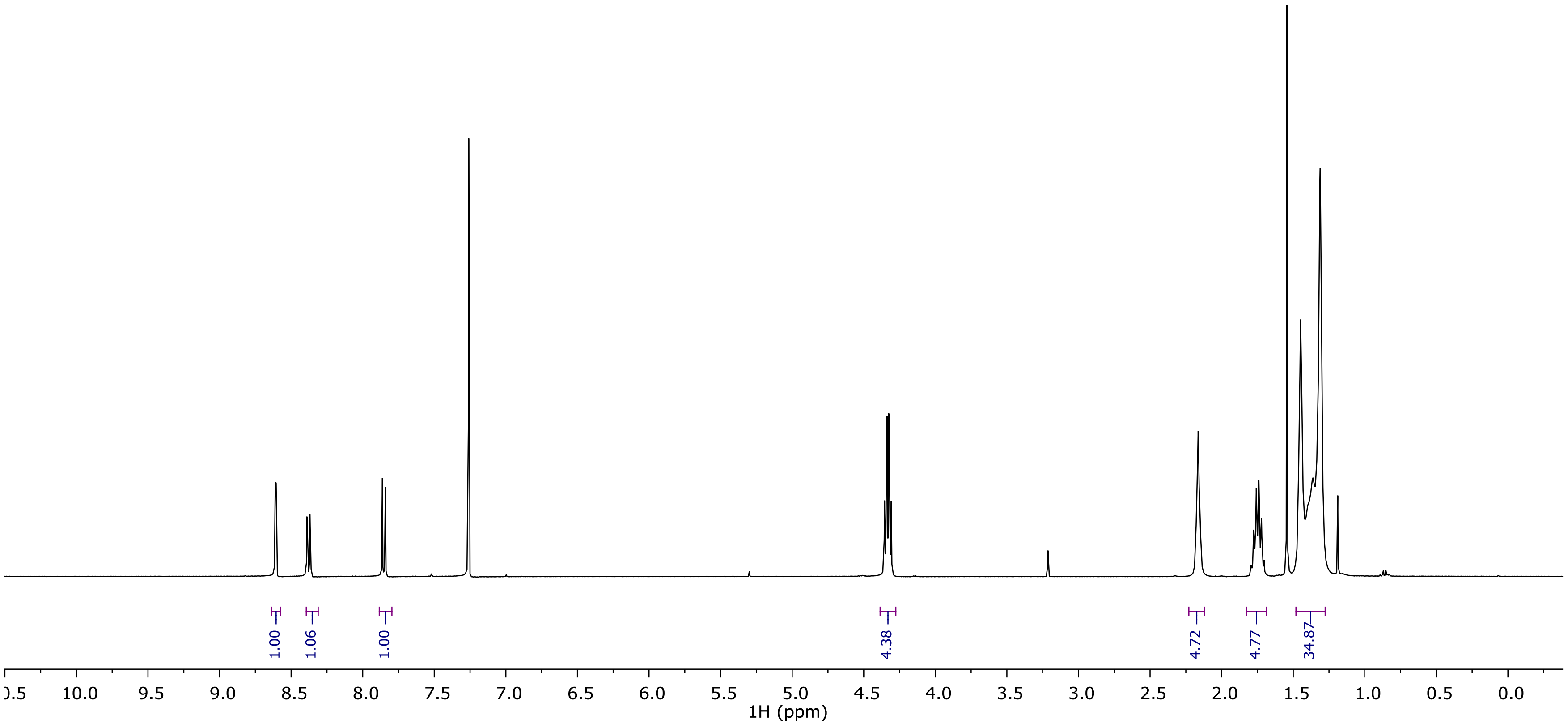

^{1}H NMR of compound S17, $CDCl_3$, 23 °C

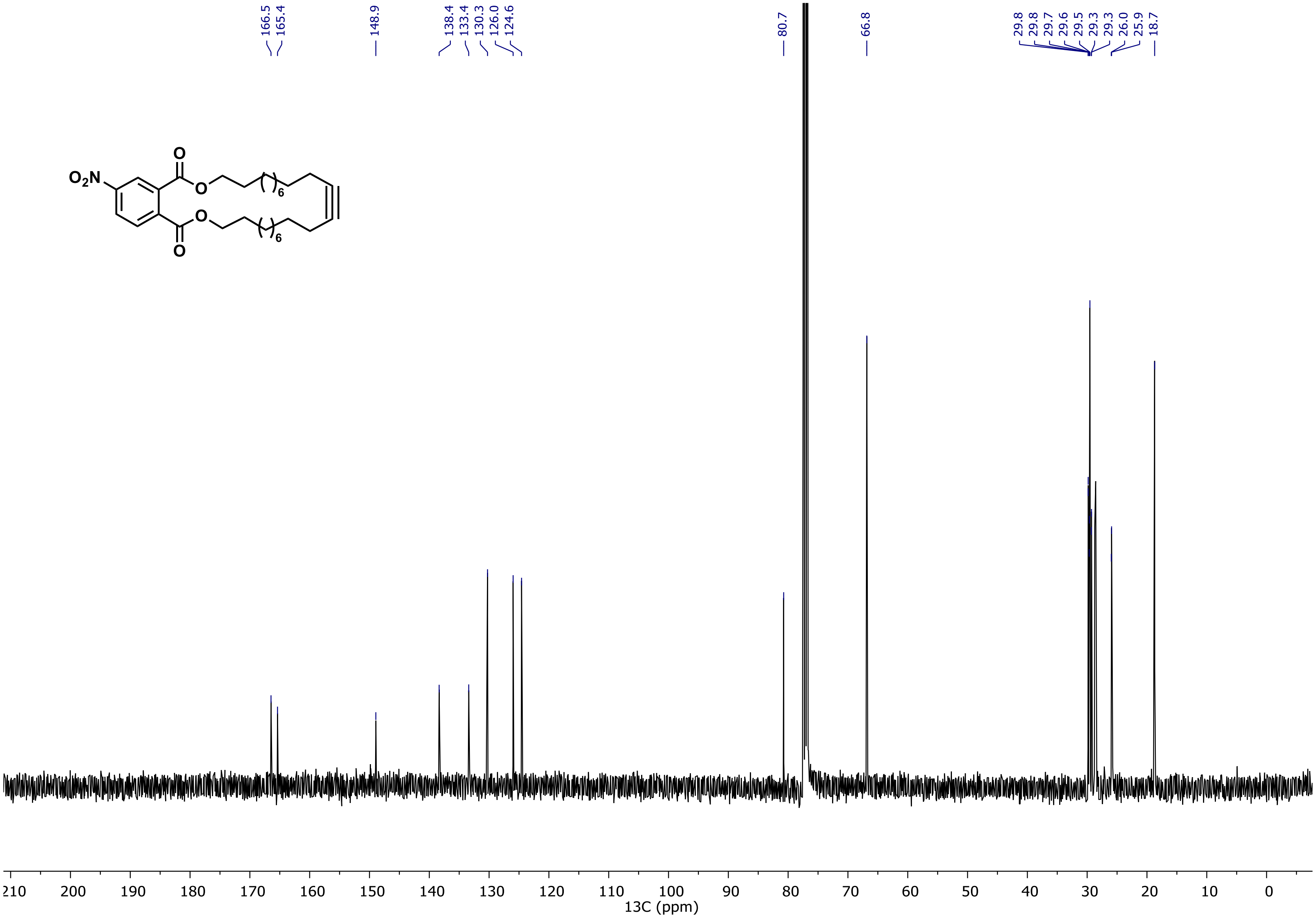

^{1}H NMR of Compound S18, $CDCl_3$, 23 ºC

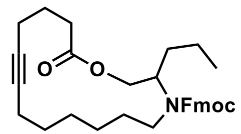

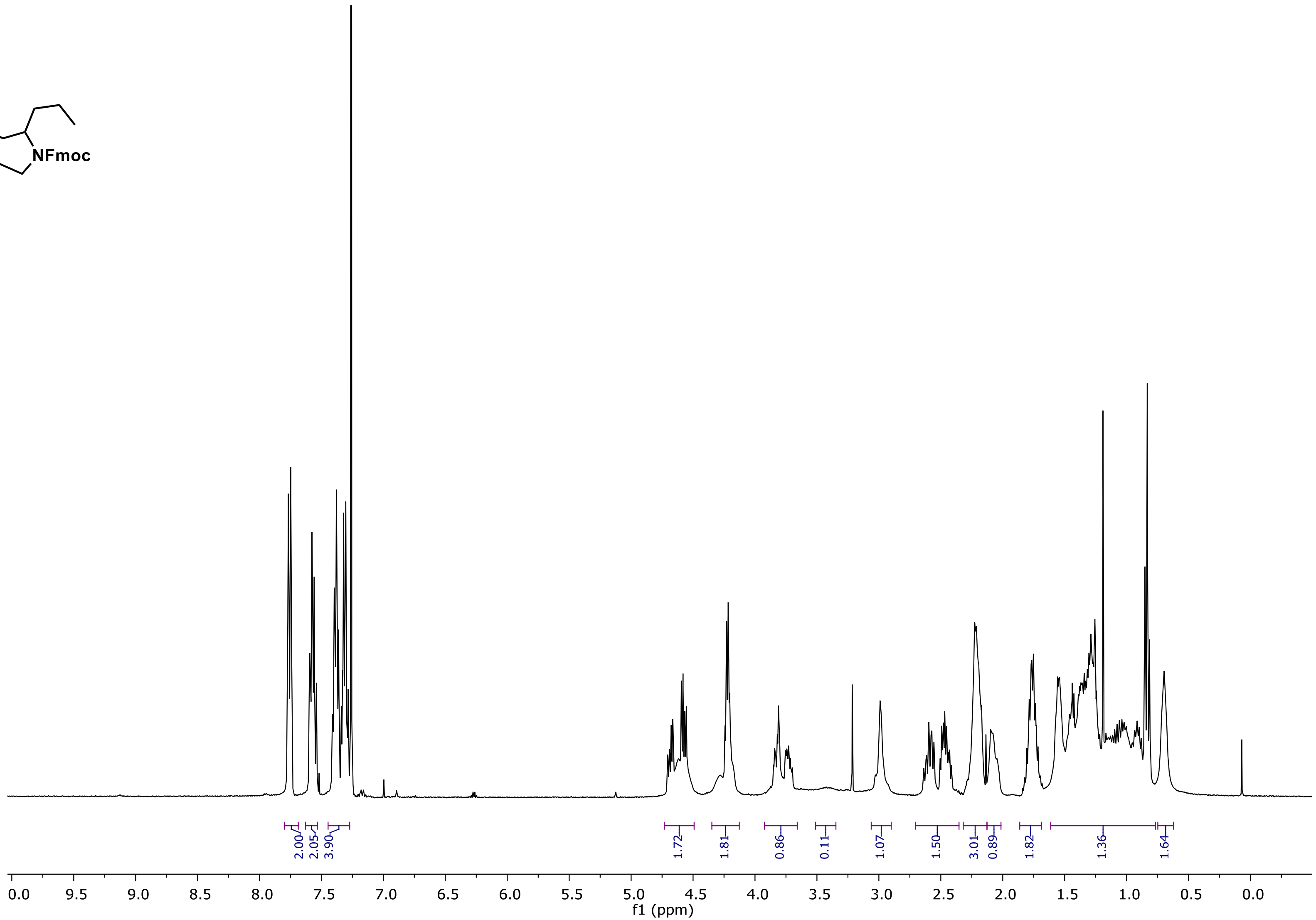

^{13}C NMR of compound S18, $CDCl_3$, 23 ºC

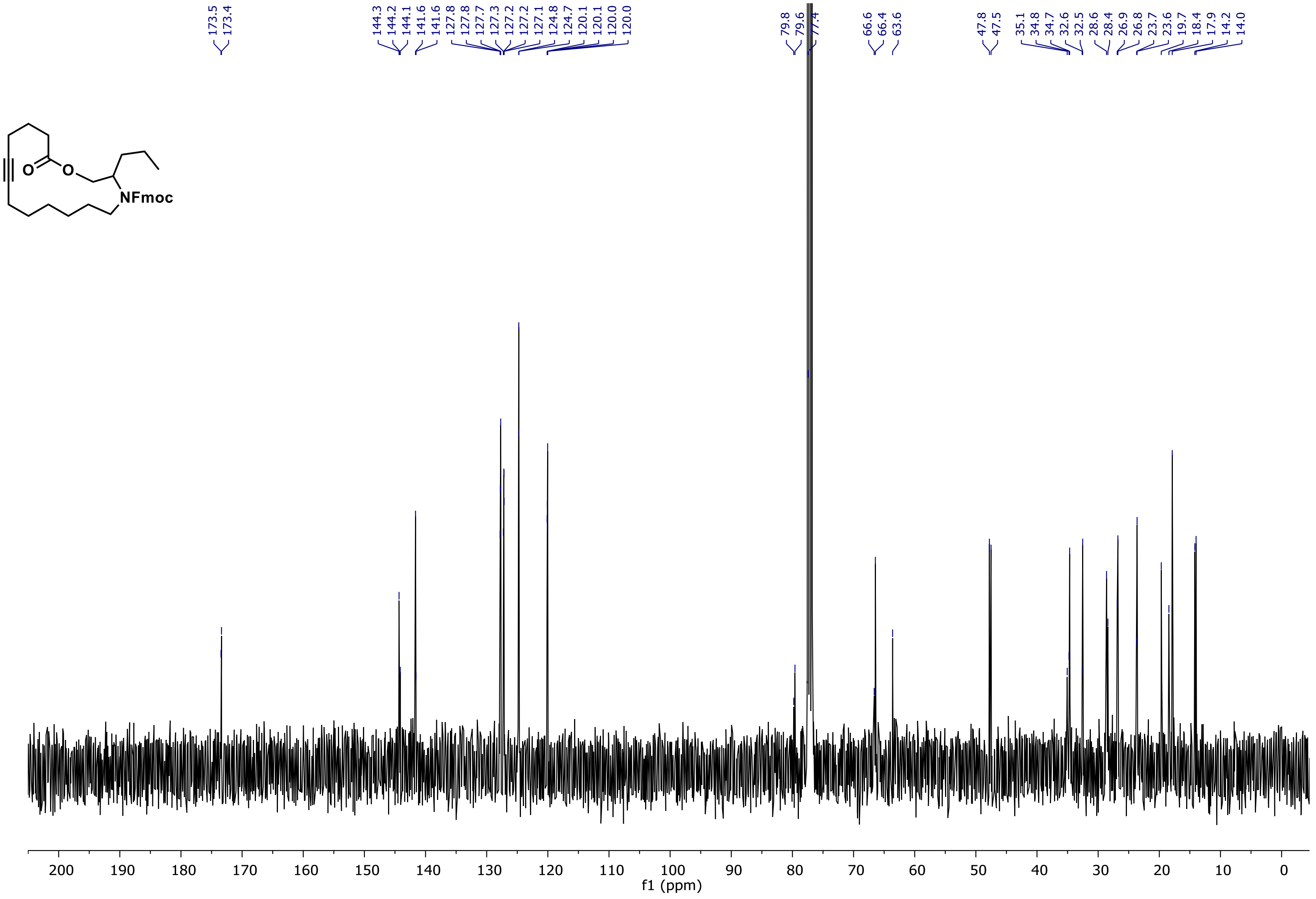

^{1}H NMR of compound S19, $CDCl_3$, 23 ºC

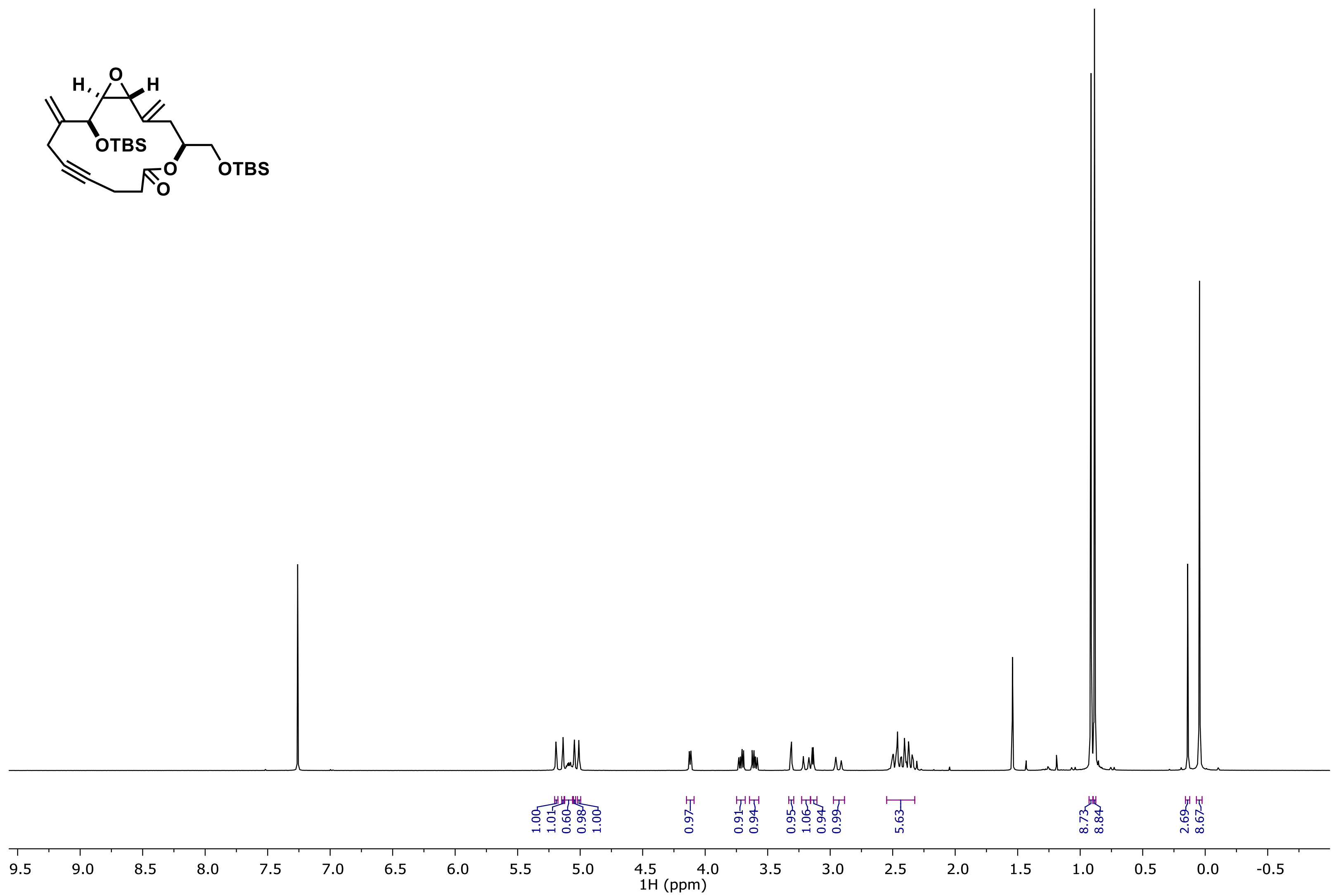

^{13}C NMR of compound S19, $CDCl_3$, 23 ºC

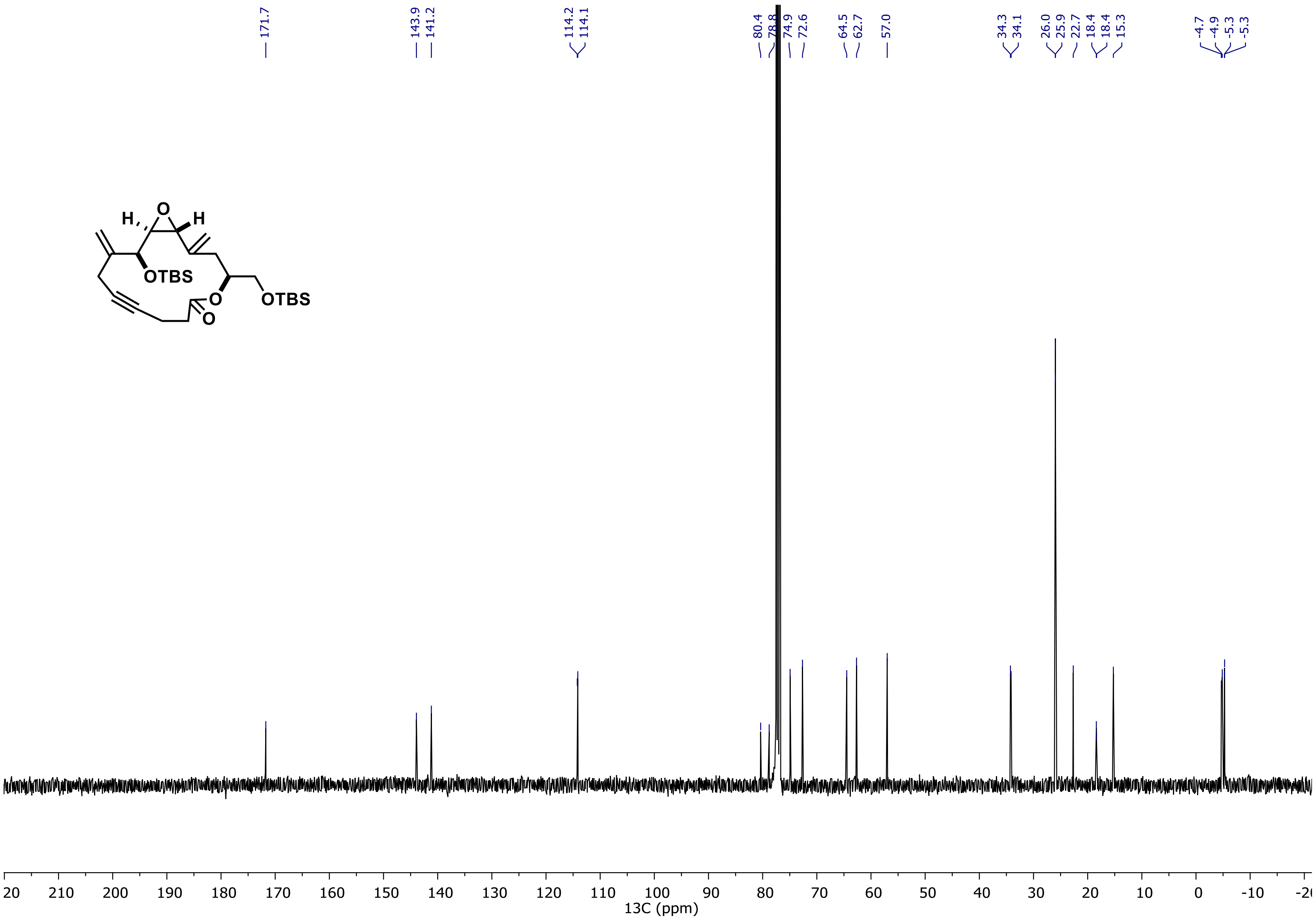

^{1}H NMR of compound S20, $CDCl_3$, 23 ºC

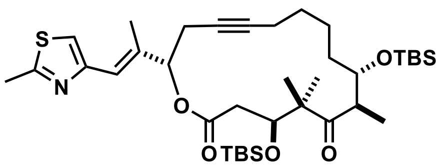

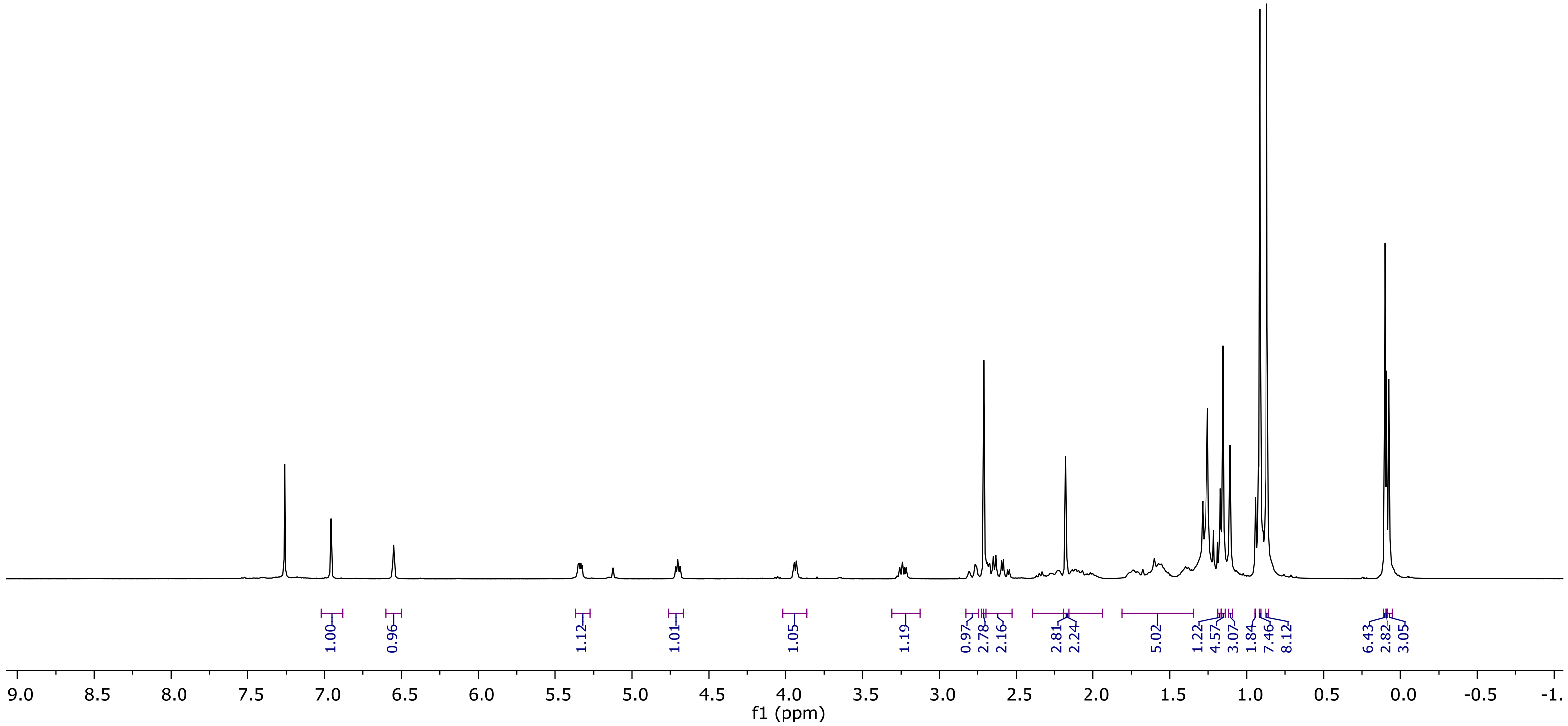

^{13}C NMR of compound S20, $CDCl_3$, 23 ºC

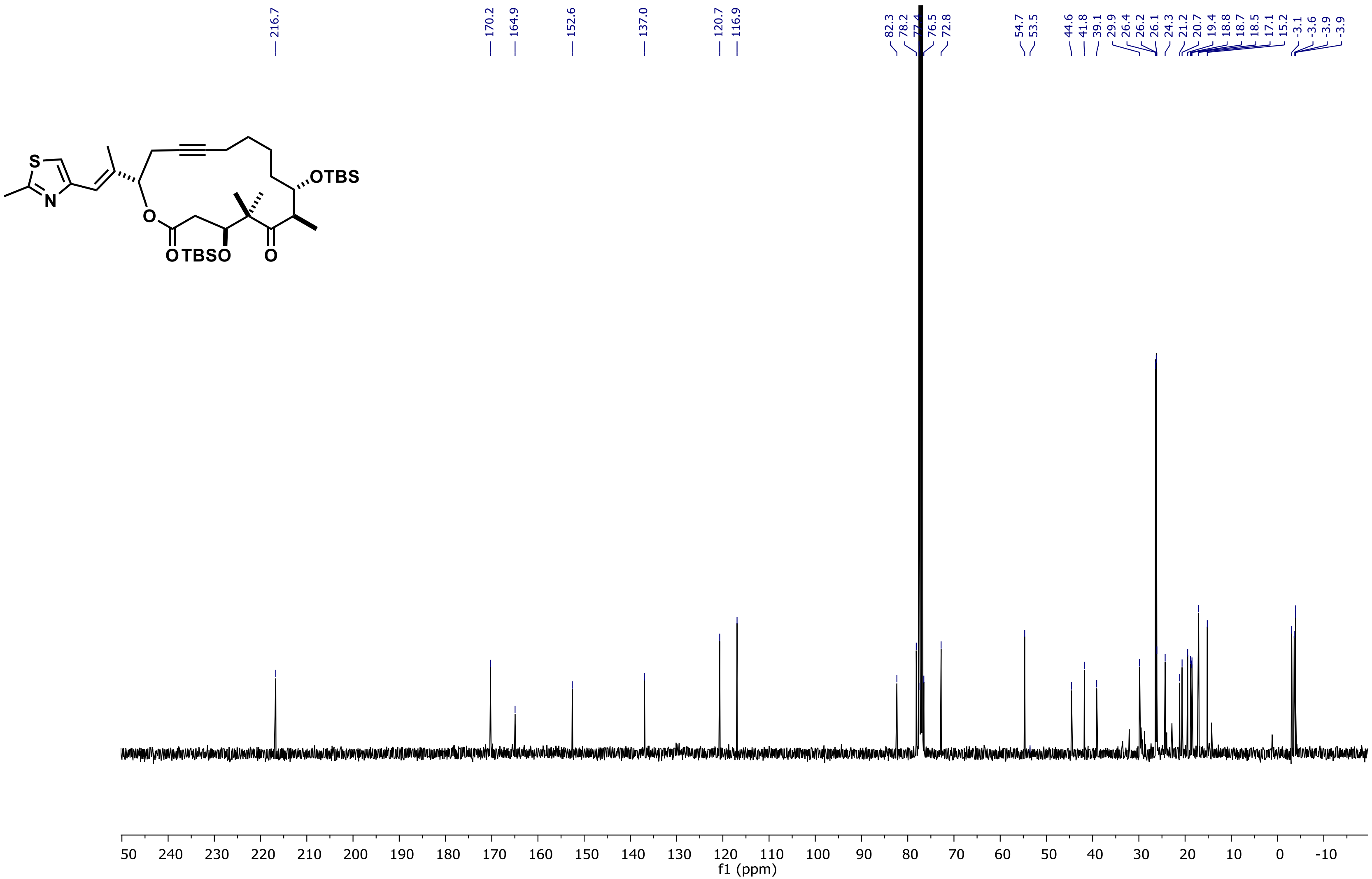

^{1}H NMR of compound S21, CDCl$_3$, 23°C

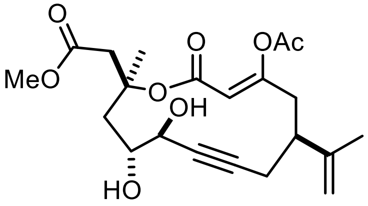

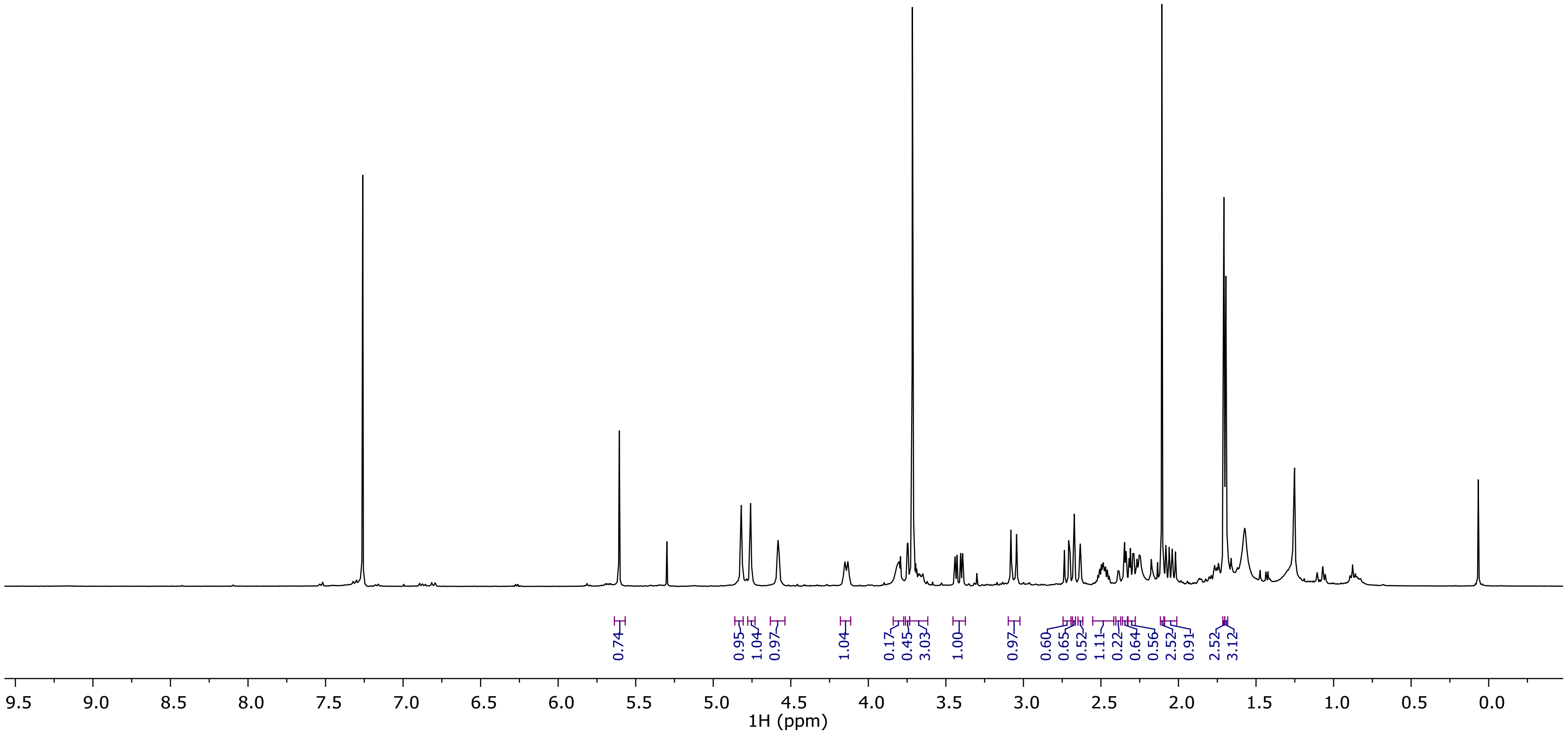

^{13}C NMR of compound S21, CDCl3, 23°C

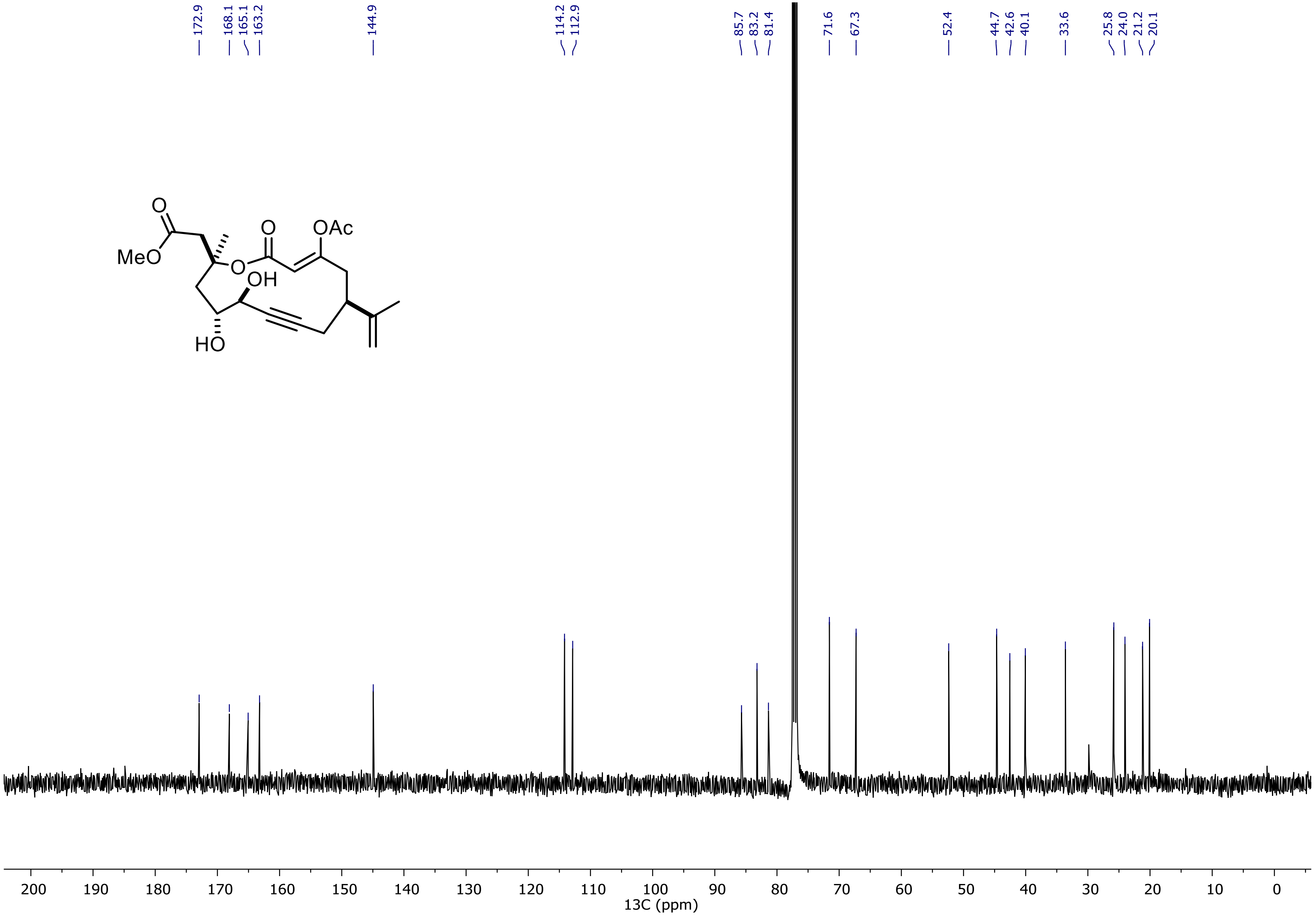

Supplement: Supplementary file 1 — Supplementary [file ANIE-58-15690-s001.pdf]
